# Periodic Table of Immunomodulatory Elements and Derived Two‐Dimensional Biomaterials

**DOI:** 10.1002/advs.202406324

**Published:** 2025-01-03

**Authors:** Alireza Rafieerad, Leena Regi Saleth, Soofia Khanahmadi, Ahmad Amiri, Keshav Narayan Alagarsamy, Sanjiv Dhingra

**Affiliations:** ^1^ Institute of Cardiovascular Sciences St. Boniface Hospital Albrechtsen Research Centre Biomedical Engineering Program Department of Physiology and Pathophysiology Rady Faculty of Health Sciences University of Manitoba Winnipeg Manitoba R2H2A6 Canada; ^2^ Institute for Molecular Biosciences Johann Wolfgang Goethe Universität 60438 Frankfurt am Main Germany; ^3^ Russell School of Chemical Engineering The University of Tulsa Tulsa OK 74104 USA

**Keywords:** 2D biomaterials, cancer therapy, immunoengineering, immunomodulatory periodic elements, nanomedicine, theranostic

## Abstract

Periodic table of chemical elements serves as the foundation of material chemistry, impacting human health in many different ways. It contributes to the creation, growth, and manipulation of functional metallic, ceramic, metalloid, polymeric, and carbon‐based materials on and near an atomic scale. Recent nanotechnology advancements have revolutionized the field of biomedical engineering to tackle longstanding clinical challenges. The use of nano‐biomaterials has gained traction in medicine, specifically in the areas of nano‐immunoengineering to treat inflammatory and infectious diseases. Two‐dimensional (2D) nanomaterials have been found to possess high bioactive surface area and compatibility with human and mammalian cells at controlled doses. Furthermore, these biomaterials have intrinsic immunomodulatory properties, which is crucial for their application in immuno‐nanomedicine. While significant progress has been made in understanding their bioactivity and biocompatibility, the exact immunomodulatory responses and mechanisms of these materials are still being explored. Current work outlines an innovative “immunomodulatory periodic table of elements” beyond the periodic table of life, medicine, and microbial genomics and comprehensively reviews the role of each element in designing immunoengineered 2D biomaterials in a group‐wise manner. It recapitulates the most recent advances in immunomodulatory nanomaterials, paving the way for the development of new mono, hybrid, composite, and hetero‐structured biomaterials.

## Graphical abstract

Two‐dimensional (2D) nanomaterials, possessing unique physiochemical properties, revolutionize the field of biomedical engineering to address critical diagnostic and therapeutic challenges. This review unveils the periodic table of 2D immunomodulatory elements, assessing synthesis, properties, immunomodulatory mechanisms and biocompatibility. A comprehensive group‐wise, element‐by‐element roadmap of nano‐enabled immunomodulatory 2D materials is provided, discussing well‐explored and emerging 2D elements, while noting those without current application in immunoengineering, drug delivery, cancer therapy and regenerative medicine.

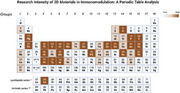

## 1 Introduction

The periodic table of chemical elements has played a significant role in advancing human health and civilization by facilitating the development of functional tools, products, and material‐based technologies. These advancements have led to the ongoing evolution of various science and engineering fields.^[^ ^1^, ^2^, ^3^, ^4^ ^]^ From a biological point of view, periodic chemical elements have previously been categorized into the elements of life,^[^ ^5^ ^]^ medicine,^[^ ^6^ ^]^ and microbial genomics.^[^ ^7^ ^]^ More recently, the emergence of inorganic biomaterials with antimicrobial and therapeutic properties has demonstrated the potential of these elements for immunoengineering applications.^[^ ^6^, ^8^ ^]^ This knowledge has further contributed to a thorough understanding of the biological properties of each element in the fabrication of chemical complexes, biomaterials and devices.

**Figure 1:**
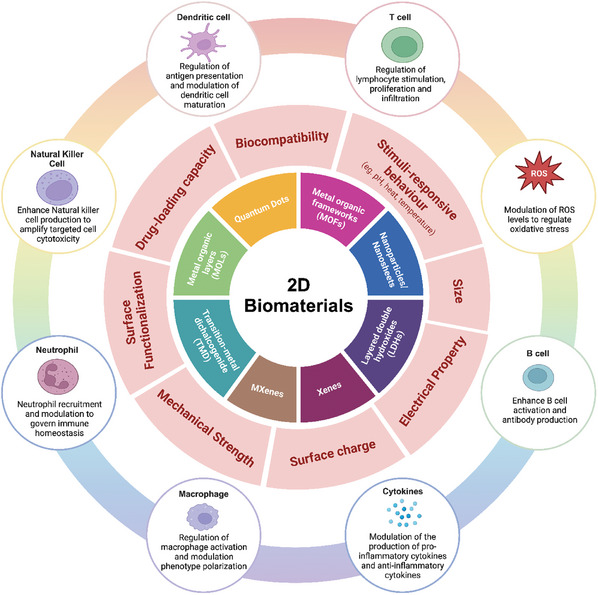
A comprehensive, multilayered schematic illustration of 2D biomaterials and their immunomodulatory mechanisms, organized in a concentric circular format. The inner circle represents the different classes of two‐dimensional biomaterials derived from the elements of the periodic table. The middle circle outlines critical physicochemical factors, such as size, surface charge, biocompatibility, stimuli responsiveness, surface functionalization, and electrical, mechanical and drug loading properties, that influence the immune response and therapeutic efficacy of these nanomaterials. The outer circle depicts diverse immunomodulatory mechanisms triggered by the interaction between 2D biomaterials and immune cells. This integrated representation demonstrates how the inherent properties of elements from the periodic table can be harnessed to engineer 2D nanoforms with specific physicochemical characteristics, directly dictating their immunomodulatory regulation in disease treatment and management. For example, owing to their unique physiochemical properties, MXenes modulate both immunosuppressive and immune tolerance pathways. Its excellent photothermal ability harnesses its immunosuppressive behavior in the tumor microenvironment, while its immune tolerance aids in the prevention of allograft rejection and supports various tissue regeneration applications. This multifunctionality demonstrates the versatility of 2D nanomaterials derived from the periodic table of elements, underscoring their potential in diverse therapeutics through tailored immunomodulation.

Accordingly, several biomaterial‐based strategies have been introduced and implemented in different medical sectors, including detection, tracking, bioimaging, targeted delivery, regenerative medicine, and cancer therapy. Two‐dimensional (2D) biomaterials are a class of nanomaterials consisting of single to few layers of atoms, typically with thicknesses in the nanometer range. This unique two‐dimensional structure, along with its phase, crystallinity and size, endows these biomaterials with superior physiochemical properties. These attributes make them outstanding candidates for a wide range of applications, including energy storage and electronic, environmental, electrochemical, biomedical and biosensing. These distinctive properties of 2D nanomaterials distinguish them from their three‐dimensional (3D) counterparts, making 2D nanomaterials highly versatile, multifunctional, ultrathin, flexible biomaterials.^[^ ^9^, ^10^ ^]^ Following the discovery of graphene's potential in biomedical applications, researchers have broadened their investigations to explore other 2D nanoforms derived from a wide array of elements across the periodic table. In addition to their well‐explored biomedical applications, the ability of 2D nanomaterials to modulate immune responses has become a significant tool in the development of immunoengineering approaches to target inflammatory and infectious diseases. This area became even more important after the outbreak of COVID‐19. Several research teams are investigating the potential of biomaterial‐based strategies for treating infectious and inflammatory diseases. Recently, the field has undergone significant growth, and several materials have been reported to have intrinsic immunomodulatory properties due to the presence of certain elements in their composition.^[^ ^9^, ^11^, ^12^, ^13^, ^14^ ^]^ Biomaterials, owing to their size and shape, are reported to interact with the host immune system through different mechanisms (**Figure** **1**).^[^ ^9^ ^]^ In summary, the high bioactive surface area and biocompatibility of 2D biomaterials have shown promise in exhibiting several key immunomodulatory mechanisms. The complex interplay of these physiochemical factors and immunomodulatory mechanisms drives the interaction of 2D biomaterials with different types of immune cells, such as macrophages, dendritic cells and T cells; modulates cytokine production; influences antigen presentation; alters immune cell signaling pathways; and regulates inflammatory responses.^[^ ^15^ ^]^ Specifically, in cancer and drug delivery applications, these biomaterials induce immune cell activation by altering the tumor microenvironment through the generation of reactive oxygen species and induce tumor cell death via their photothermal, photodynamic or sonodynamic therapeutic potential. Conversely, in the case of inflammatory and infectious diseases, these 2D nanomaterials promote immune tolerance by suppressing the activation and proliferation of immune cells, aiding in understanding the potential of 2D nanomaterials in immunotherapy. The chemical composition of a material also plays a very important role in defining its properties and mechanisms of action. Several in vitro and in vivo studies have reported the role of the chemical composition of biomaterials in their immunomodulatory behavior.^[^ ^11^, ^12^, ^13^, ^14^, ^16^ ^]^ Despite the advances in nanoimmunoengineering and the importance of the field as such, there has been no report on the compilation of a review on the classification of immunomodulatory elements beyond the periodic table of life, medicine, and microbial genomics.^[^ ^6^, ^7^ ^]^

Therefore, in the current review, we classify and design innovative “immunomodulatory periodic elements” and derive 2D biomaterials as a comprehensive reference for researchers in the field. We have compiled a group‐by‐group and element‐by‐element journey through the periodic table of chemical elements in a systematic manner. We have discussed the current advances in the synthesis, properties, and biomedical applications of these materials. We have discussed different methods of preparation, such as self‐assembly, exfoliation, epitaxy, thin‐film coating, colloidal accumulation, and matrix reinforcement. First, groups 1 to 2 (hydrogen and alkali metals and alkaline earth metals) are discussed, followed by groups 3 to 12 (transition metals, lanthanides and actinides). Typically, Group 1 and Group 2 elements exist in conventional 2D forms such as nanoparticles and nanosheets. The elements in groups 3 to 12 are the widely used 2D forms of nanomaterials. The inclusion of transition metals in these nanocomposites imparts unique properties, making them suitable for a diverse array of applications. They are specialized immunoengineered nanostructures such as Xenes, MXenes, transition‐metal dichalcogenides (TMDs), transition metal oxides (TMOs), metal‒organic frameworks (MOFs), metal‒organic layers, and layered double hydroxides (LDHs). As illustrated in Figure 1, these transition metal‐based 2D nanostructures exhibit a wide range of immunomodulatory properties, aiding in cancer therapy, diagnostics and the management of inflammatory diseases. Next, groups 13 to 17, comprising p‐block elements, mostly metalloids, posttransition metals, and reactive nonmetals, are discussed. Some of the elements in these groups have very well‐known and extensively researched 2D forms such as graphene and black phosphorus, as well as monoelemental nanomaterials. Finally, we conclude this unique roadmap of immunomodulatory elements by describing the role of group‐18 noble gases in the synthesis and functionalization of 2D biomaterials.

**Figure 2:**
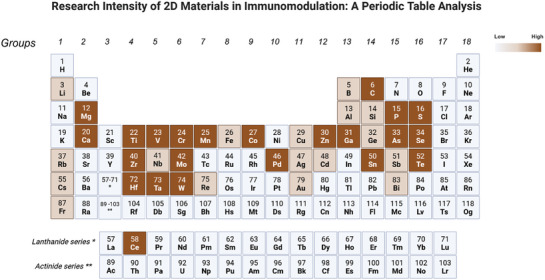
Research intensity of 2D materials in immunomodulation: a periodic table analysis. Elements are arranged by increasing the atomic size (right to left, top to bottom, groupwise). Dark brown indicates elements with extensive studies, light brown indicates moderate‐ and early‐stage studies, and gray indicates no reported usage in immunomodulation studies.

Because of the potential of immunomodulatory biomaterials in the field of biomedical applications, efforts toward clinical translation and regulatory approvals will soon be underway. Therefore, to expand the scope of this review article, we have included a separate section to summarize the in vitro and in vivo biocompatibilities of 2D nanomaterials.^[^ ^17^, ^18^, ^19^, ^20^, ^21^, ^22^, ^23^ ^]^ An overview of the content covered in this report is described in **Figure** **2**. We believe that this review will help experts in the field gain the comprehensive knowledge required to optimize and fabricate new mono‐, hybrid‐, and composite‐based immunomodulatory biomaterials for nanomedicine applications. (**Table** **1**)

**Table 1:** List of immunomodulatory 2D nanomaterial composites derived from periodic table elements.

Element	Immunomodulatory 2D nanomaterial composites	Chemical formula
Lithium (Li)	Lithium nanosheets Lithium‐doped‐hydroxyapatite	Li‐NSs Li‐HAP
Rubidium (Rb)	Rubidium‐hydroxyapatite	Rb‐HAP
Cesium (Cs)	Nanocesium	nCs
Magnesium (Mg)	Magnesium oxide Magnesium hydroxide nanosheets Magnesium phosphate nanosheets Magnesium‐boron nanosheets in polyvinylpyrrolidone	MgO MgOH‐NS Mg_3_(PO_4_)_2_‐NS MgB‐NS‐PVP
Calcium (Ca)	Nano calcium Calcium Phosphate Calcium fluoride	Ca‐N Ca_3_(PO_4_)_2_ CaF_2_
Cerium (Ce)	Cerium nitrate Nano Ceria (Cerium oxide nanoparticles) Cerium‐hydroxyapatite Polyethylene glycol‐treated (PEGylated) cerium oxide nanoparticles	Ce(NO_3_)_3_ CeO_2_‐NPs Ce‐HAP CeNPs‐PEG
Titanium (Ti)	Titanium carbide MXene Titanium carbide MXene‐doxorubicin Titanium carbide MXene‐ Manganese oxide Titanium Oxide	Ti_3_C_2_TX T_3_C_2_‐DOX Ti_3_C_2_‐MnO_x_ TiO_2_
Zirconium (Zr)	Zirconium based 2D metal‐organic frameworks	Zr‐MOFs
Hafnium (Hf)	Hafnium oxide Hafnium based two‐dimensional metal organic layers Hafnium nanoparticles Tannic acid coated hafnium disulfide nanosheets Hafnium‐doped Carbon Dots	HfO_2_ HfO_2_‐2D‐MOLs Hf‐NPs HfS_2_‐TA‐NSs Hf‐CDs
Vanadium (V)	Vanadium oxide Vanadium oxide nanoparticles Vanadium pentoxide Vanadium pentoxide nanoparticles Vanadium carbide MXenes	V_2_O_3_ V_2_O_3_‐NPs V_2_O_5_ V_2_O_5_‐NPs V_2_CT_x_
Niobium (Nb)	Niobium carbide MXenes	Nb_2_CT_x_
Tantalum (Ta)	Tantalum carbide MXenes Iron‐oxide nanoparticles functionalized Tantalum carbide MXenes Manganese oxide nanoparticles functionalized Tantalum carbide MXenes Tantalum carbide MXene‐tantalum oxide Tantalum carbide MXene Quantum Dots	Ta_4_C_3_T_x_ Ta_4_C_3_‐IONPs Ta_4_C_3_T_x_ ‐MnO_x_ TTO Ta_4_C_3_T*_x_* MQDs
Chromium (Cr)	Nano chromium picolinate Chromium‐based MXenes Chromium nanoparticles	nCrPic Cr_2_CT_x_ CrNPs
Molybdenum (Mo)	Molybdenum carbide MXenes Molybdenum carbide MXenes surface modified with polyvinyl alcohol Molybdenum disulfide nanosheets PEGylated Molybdenum disulfide nanosheets Copper oxide Molybdenum disulfide composites	Mo_2_CT_x_ Mo_2_CT_x_‐PVA MoS_2_‐NSs PEG‐MoS_2_‐NSs CuO‐MoS_2_
Tungsten (W)	Tungsten oxide Tungsten oxide nanosheets PEGlylated iron tungsten oxide nanosheets Tungsten oxide decorated gold nanoparticle hybrids Tungsten disulfide Poly(vinyl pyrrolidone)‐tungsten disulphide nanosheets Tungsten disulphide quantum dots Erbium doped tungsten selenide	WOx WO‐NSs FeWO‐PEG‐NSs AuNP‐WO_x_ WS_2_ PVP/WS_2_‐NSs WS_2_QDs Er‐WSe_2_
Manganese (Mn)	2D manganese dioxide Manganese based metal‐organic frameworks Mn was doped with cobalt molybdenum double layered hydroxide	MnO_2_‐2D Mn‐MOFs Mn‐CoMo‐LDH
Rhenium (Re)	Rhenium disulfide nanosheets	ReS_2_‐NSs
Iron (Fe)	Iron Oxides	Fe_2_O_3_
Cobalt (Co)	Cobalt hydroxide nanosheets Iron, cobalt, and polyethylene glycol composite Cobalt tungsten layered double hydroxide Manganese doped cobalt molybdenum layered double hydroxide	Co(OH)_2_‐NSs FeCO‐PEG CoW‐LDH Mn‐CoMo‐LDH
Palladium (Pd)	Palladium nanosheets	Pd‐NSs
Copper (Cu)	Copper Layered double hydroxide Gadolinium copper layered double hydroxide	Cu‐LDH GdCu‐LDH
Silver (Ag)	Silver nanosheets Silver nanoparticles	Ag‐NSs Ag‐NPs
Gold (Au)	Gold nanostructures Gold nanoparticles/nanogold	Au‐NSs Au‐NPs
Zinc (Zn)	Nanozinc Zn based metal‐organic frameworks Zn based layered double hydroxide	nZn Zn‐MOFs Zn‐LDHs
Boron (B)	PEGylated Borophene nanosheets Borophene nanosheets	PEG‐B‐NSs B‐NSs
Aluminum (Al)	Aluminum hydroxide nanosheets	Aloohene
Gallium (Ga)	Gallium nanoparticles Nanocomposite of Graphene oxide – gallium nanoparticles Gallium‐based gelatin nanoparticles loaded with quercetin Gallium‐based metal‐organic frameworks	Ga‐NP GO‐GaNPs QCT@GNPs‐Ga Ga‐MOFs
Carbon (C)	Graphene oxide Graphene oxide nanosheets Carbon dots Carbon nanotubes Curdlan‐decorated fullerene nanoparticles	GO GO‐NSs CD CNTs Cur‐F‐NPs
Silicon (Si)	Silica nanoparticles Silicene nanosheets	SiO_2_‐NPs nSi‐NSs
Germanium (Ge)	2D germanene nanosheets	nGe‐NSs
Tin (Sn)	Tin nanoparticles Cerium oxie/Tin oxide Nanoparticles Tin oxide nanoparticles Indium‐tin oxide	Sn‐NPs CeO_2_/SnO_2_‐NPs SnO_2_‐NPs ITO
Phosphorus (P)	Black phosphorus Black Phosphorus Nanosheets Carbon dot‐passivated BP nanosheets Phosphorene Calcium phosphate nanoparticles	BP BP‐NSs CD‐BP‐NSs nP CaP‐NPs
Arsenic (As)	Arsenic nanomaterials Arsenene nanosheets	As‐NMs nAs‐NSs
Antimony (Sb)	Antimony oxide nanoparticles Antimonene Nanosheets	Sb_2_O_3_‐NPs nSb‐NSs
Bismuth (Bi)	Bismuth nanoparticles Bismuthene nanosheets	Bi‐NPs nBi‐NSs
Sulfur (S)	Sulfur nanoparticles Nanoselium sulfur composite Sulfurene	S‐NPs nSeS nS
Selenium (Se)	Selenium nanoparticles Selenium nanoparticles with mannose‐rich oligosaccharides	Se‐NPs Se‐NP‐MRO
Tellurium (Te)	Tullurene Tellurene nanosheets modified with polyethylene glycol Tellurium nanoparticles Tellurium nanowires	nTe nTeNS‐PEG Te‐NPs Te‐NWs

## 2 Periodic Immunomodulatory Table Element‐Derived 2D Biomaterials

### 2.1 Biocompatibility Assessment and Safety Patterns of 2D Biomaterials

Low‐dimensional biomaterials, especially layered 2D nanomaterials, are critical in developing future technologies for the biomedical and medical fields because of their greater biocompatibility than conventional bulk materials. Studies have investigated the biocompatibility, efficacy and safety of these nanomaterials, providing insights into their molecular and cellular interactions with biological moieties to assess their biocompatibility and nanotoxicological profiles both in vitro and in vivo. The bioactivity of these atomically thin‐structured biomaterials is enhanced by the presence of abundant functional groups on their surface, leading to improved biological interactions. Numerous studies have investigated the biocompatibility and dose optimization of modern nanomaterials, as different forms of nanomaterials interact with biological systems through complex and distinct mechanisms. The subsequent sections delve into these investigations, as they are crucial in ensuring the safe and effective use of immunodoluatory nanomaterials in biomedicine and healthcare. Nevertheless, advancements in the bioengineering of 2D nanomaterials have focused on manipulation of their microstructure, biodegradation, and dose optimization. In particular, bioactive nanomaterials exhibit negligible toxicity at controlled doses at both the cellular and tissue/organ levels.^[^ ^24^ ^]^ Despite advancements in the bioengineering of 2D nanomaterial immunomodulatory nanomaterials, assessing their safety efficacy is critical. of these materials. Current findings are in their infancy and require further investigations into long‐term immunological effects. Therefore, in later sections, we discuss strategies such as combining 2D nanomaterial composites with biodegradable polymers, functionalizing them with surface modifications, engineering them as smart delivery platforms, maximizing their therapeutic benefits while minimizing potential adverse effects, and translating the unique properties of 2D materials into clinically relevant applications. **Table** **2** provides a concise summary of the biocompatibility of commonly used 2D layered biomaterials.

**Table 2:** Representative biocompatibilities of 2D layered nanomaterials and derived composites.

2D Bio‐Nanomaterial	Dose & Time	In vitro/vivo Model	Biological Applications	Biocompatibility & Degradations	Refs
Antimonene Nanosheets‐derived quantum dots	Up to 200 µg mL^‒1^ for 24 hours	Cells HEK 293, HeLa, PC3, MCF7, A549	Photo‐thermal therapy	No visible cytotoxicity to cells. cells viability of approximately 80 per cent	[25]
As_2_Se_3_ (Arsenic nanosheets)	Up to 8 µg/ml without light irradiation	4T1 cells	Photo‐thermal therapy	As_2_Se_3_ showed no visible cytotoxicity in the dark; however, it became toxic at a concentration of 0.5 µg/ml upon irradiation.	[26]
Black phosphorous nanosheets	Up to 200 µg mL^‒1^ in 4 T1 for 24 hours	Cell HeLa, L929, and A549		No obvious cytotoxicity to cells, cells viability of approximately 95 percent. Degradation during 24 h in PBS	[27]
Boron nanosheets	Up to 500 µg mL^‒1^ for 48 hours	Cells HeLa, PC3, MCF7, and A54	Multimodal imaging‐guided cancer therapy	No particular cytotoxicity to cells. Cells viability of 90% and good drug‐loading capacity	[28]
Graphene oxide nanosheets	Up to 100 µg mL^‒1^ for 48 hours	MDA‐MB‐231 cells	Photothermal therapy	No visible cytotoxicity to cells. cells viability of approximately 80 per cent	[29]
Graphitic carbon nitride nanosheet‐MOF	Up to 100 µg mL^‒1^ for 24 hours	Cells A549	Photo‐thermal therapy	No visible cytotoxicity to cells. 90% cell viability	[30]
Layered double hydroxides	Up to 500 µg mL^‒1^ for 24 hours	Cell HeLa, U87 mg, KB and HepG2		No visible cytotoxicity to cells. cells viability of approximately 90 per cent	[31]
Manganese dioxide	Up to 200 µg mL^‒1^ for 24 hours	Cells MDA‐MB‐23	Bioimaging and oxygen elevated therapy	No significant cytotoxicity to cells. cells viability of approximately 80 per cent	[32]
Molybdenum disulfide nanosheets	Up to 100 µg mL^‒1^ for 24–50 hr	Cells (RAMEC and PC12)	Biosensors neurotransmitter & glucose	No obvious toxicity with higher cell viability of 75%	[33]
Nb_2_CT_x_ nanosheets	10 to 200 µg mL^‒1^ for 24 hours	Murine breast cancer cell lines (4T1and U87)	Photo‐thermal therapy	No significant toxicity to the tested cells dose up to at 200 µg mL^‒1^ with laser intervention	[34, 35]
Polyvinyl pyrrolidone‐coated Fe_3_S_4_ nanosheets	Up to 200 µg mL^‒1^ for 24 hours	Cell lines HeLa	Bioimaging photo thermal/chemodynamic therapy	No significant cytotoxicity to cells. cells viability of approximately 80 per cent	[36]
Ti_3_C_2_T_x_ MXene nanosheets	100 and 500 µg mL^‒1^ for seven days	Human umbilical vein endothelial cells	Metabolomics approach to evaluate the toxicity of material and their cell interactions	No significant differences were observed between the viability of cells in control & Ti_3_C_2_T_x_ groups.	[37, 38]
Ti_3_C_2_T_x_ MXene nanocomposite	0 to 100 µg mL^‒1^ for seven days	Human mesenchymal stem cells	Bone tissue engineering	Ti_3_C_2_T_x_ at the dose > 50 µg mL^‒1^ showed cytotoxicity to tested cells; However, at lower dose than 50 µg mL^‒1^, no significant toxicity observed.	[39]
Ti_3_C_2_T_x_ nanosheets	0.0 to 25 µg mL^‒1^ for 24 hours	Neural stem cells and derived differentiated cells	Neural tissue engineering	Ti_3_C_2_T_x_ at the concentration > 25 µg mL^‒1^ showed cytotoxicity to tested cells; However, at higher dose than < 12.5 µg mL^‒1^, no significant toxicity was observed.	[40]
Multi, single, mono‐layered Ti_3_C_2_T_x_ MXene nanoflakes, and TiC, Ti_2_AlC, Ti_3_AlC_2_ bulky MAX phases	10 to 400 µg mL^‒1^ for 24 and 48 hours	Human fibroblast lines (MSU1.1)	Bioinstrumentation	A concentration‐dependent cytotoxicity was observed in the tested MAX phases. 2D MXenes showed no significant toxicity compared to control groups	[41]
Ti_3_C_2_T_x_ nanocomposite	25 to 200 µg mL^‒1^ µg/mL for 96 hr postfertilization	Zebrafish embryo model	Biomedical applications	Ti_3_C_2_T_x_ at the concentrations of 50–100 µg mL^‒1^ showed no significant toxicity, teratogenic, and neurotoxic effects in neuro‐muscular activity	[42]
2D MXene Ti_3_C_2_T_x_	0.0 to 200 µg mL^‒1^ for 4 hours	Vero E6 cells	Anti‐viral & Immunomodulatory effect	No significant toxicity toward the tested cells up to 200 µg mL^‒1^	[43]
V_2_C MXenzyme	0.0 to 400 µg mL^‒1^ for 24 24 hours	Cell lines (L929 and PC12)	ROS/scavenger for treatment of neurodegenerative diseases	No visible cytotoxicity to cells was detected even at 200 µg mL^‒1^	[44]

### 2.2 Group‐1 to Group‐2 Elements and Reported Immunoengineering Applications

The group‐1 elements consisted of hydrogen (H) and alkali metals, including lithium (Li), sodium (Na), potassium (K), rubidium (Rb), cesium (Cs), and francium (Fr). Among these alkali elements, sodium and potassium are among the most abundant elements, ranking 6^th^ and 8^th,^ respectively, in the Earth's crust. While hydrogen is abundant in the universe, it is less prevalent in the Earth's crust, ranking 10^th^. In contrast, lithium, rubidium and cesium are relatively rarer in nature. Francium is quite unstable and only exists in trace amounts with high levels of radioactivity. Despite this contrast, the elements of group 1 are extensively involved in the composition of several important materials and compounds in our environment and life. Potassium‐based nanomaterials are mostly considered for targeted delivery, antimicrobial properties, antioxidant activities and fertilizers in phytobiology and agricultural fields. However, to the best of our knowledge, no significant studies have reported the immunomodulatory properties of 2D potassium nanosheets for immunoengineering, immunotherapy, anticancer, and other regenerative medicine applications. Therefore, the current section is intended to review the immunomodulatory profiles of the periodic elements of the other group 1‐derived 2D materials.

#### 2.2.1 2D Lithium‐Based Nanocomposites

Lithium, the lightest solid element in the periodic table, exhibits unique physical and optoelectronic properties in its 2D forms, which is responsible for its biomedical applications.^[^ ^23^ ^]^ The biomedical applications of lithium are mostly reported for its chemical salt components, such as lithium carbonate, citrate, and sulfate, as mood‐stabilizing drugs for the treatment of bipolar disorders and depression diseases.^[^ ^45^ ^]^ In addition, energy storage bioelectronics consisting of conventional and nanolithium‐ion batteries are utilized for implantable devices such as drug pumps, neurostimulators, and cardiac pacemakers.^[^ ^46^ ^]^

Owing to the high reactivity of alkali metals, conventional wet chemical methods are usually unsuitable for lithium‐based nanomaterials that require specific synthesis equipment. Recently, the first evidence of the electrochemical growth of free‐standing lithium nanosheets was reported,^[^ ^47^ ^]^ where ultrathin lithium nanosheets (Li‐NSs) with an average thickness of approximately 10 nm were used. Compared with plasmonic heavy metals, the obtained layered materials had improved biocompatibility and biological properties with low dielectric losses, suitable plasmon absorption, and surface plasmon resonance in the visible wavelength range. Therefore, Li‐NSs have the ability to be used as biosensors and in bioimaging applications for the diagnosis and treatment of different inflammatory degenerative diseases.^[^ ^48^ ^]^

Furthermore, the potential of lithium‐hydroxyapatite (Li‐HAP) as a scaffold material was recently investigated for bone tissue engineering applications.^[^ ^49^ ^]^ Wang et al reported the synthesis of a lithium‐silicate‐chitosan‐sodium alginate nanocomposite via a typical vacuum freeze‒drying methodology with solid porosity, morphological plasticity, controlled swelling ability and biodegradability. Similarly, the development of porous Li‐HAP nanocomposites has been reported for the treatment of glucocorticoid‐induced osteonecrosis of the femoral head (GIONFH).^[^ ^50^ ^]^ The designed composite scaffold aided in upregulating the wingless‐related integration site (Wnt)/beta‐catenin (β‐catenin) and hypoxia‐inducible factor 1‐alpha (HIF‐1α)/vascular endothelial growth factor (VEGF) pathways, which are vital for repairing GIONFH. This work emphasized the use of this nanolithium material to effectively increase the bone density, promoting the osteogenic differentiation process of bone morrow‐derived mesenchymal stem cells (BM‐MSCs) and simultaneously improving osteogenic angiogenesis and the regeneration process. The Wnt and GSK‐3β pathways modulate the production of inflammatory cytokines during inflammation and are identified as targets for inflammation‐mediated diseases. The regulatory effects of lithium‐based scaffolds on the Wnt/GSK‐3β signaling pathway reported in this study suggest the use of lithium‐based nanomaterials in future inflammatory disease therapeutics.

In addition, nanolium‐based materials hold promise for the treatment of specific neurodegenerative diseases. Lithium‐based drugs can be widely distributed in the central nervous system and effectively interact with neurotransmitters and receptors to decrease the release of norepinephrine and increase serotonin production in the body.^[^ ^51^, ^52^ ^]^ Serotonin recruits innate immune cells and plays an important role in regulating inflammation within the central nervous system and periphery. It is a key regulator of mood, anxiety and immune responses related to major disruptive disorders. Owing to the interrelationship between serotonin and immune cell signaling, these factors can be effective treatments for neuroinflammation‐related disorders. Lithium‐based drugs show potential in the treatment of neurodegenerative diseases by increasing serotonin production, suggesting their immunomodulatory role in neuroinflammation. These desirable properties impact the immunomodulatory properties of these compounds by decreasing their immunosuppressive and bacterial growth‐promoting effects.^[^ ^53^ ^]^ To date, immunoengineering applications of 2D lithium nanosheets face challenges associated with large‐scale production, stability and clearance after delivery. Thus, considering the therapeutic effects of nontoxic nanolium compounds from the above‐discussed reports, these compounds can be considered potential candidates for future immunoengineering applications.

#### 2.2.2 2D Sodium‐Based Nanocomposites

Sodium‐based compounds are required in specific amounts for the body to maintain the balance of the physical fluid system and to assist in muscle and nerve functions. Sodium‐based nanoforms have been widely studied for their antibacterial properties, and injectable sodium‐containing composites formed through a typical gelation process have been reported for antibacterial applications.^[^ ^54^ ^]^ The designed scaffolds exhibited favorable physicochemical properties and were designed to combat bacterial infections, suggesting that the nanoenabled innate functionality attracts immune cells and postactivation of immunomodulatory responses. Elsewhere, a sustainable application of nanosodium‐containing filters was reported for the extraction of bacterial organisms from polluted water in different soil samples.^[^ ^55^ ^]^ This study paved the way for the use of nanosodium to overcome the current challenges of low availability of drinking water resources, human health problems and inflammatory diseases that can be caused by polluted water.^[^ ^56^, ^57^, ^58^ ^]^

Bioelectronic medicine is an emerging field of modern medicine used to develop treatment strategies for various neurophysiological and immunological challenges. Furthermore, sodium nanocompounds have been reported for next‐generation biomedical, wearable electronics and lithium‐based hybrid batteries,^[^ ^59^ ^]^ and ultrasonic crystallized cathodes have been used for metal‐ion batteries.^[^ ^60^ ^]^ The electrochemical properties of this material resulted in favorable energy storage capacity, with enhanced sodiation/desodiation properties for next‐generation sodium‐ion‐based bioelectronics for modern medicine to treat infections and inflammatory disorders.

#### 2.2.3 2D Rubidium‐Based Nanocomposites

Rubidium, the sixteenth most abundant element in the Earth's crust, is the first alkali metal in Group 1 and has a density higher than that of water. Progress in the recent development of rubidium‐based nanocompounds is rare, and their composite applications are mostly reported in optoelectronics because of their enhanced thermal stability.^[^ ^61^ ^]^ With photoluminescence (PL) emission peaks at approximately 490 nm, nanorubidium significantly enhanced the PL quantum yield (approximately 100%), suggesting a new design of quantum‐confined crystalline materials for future applications in air purification and water sterilization.

**Figure 3:**
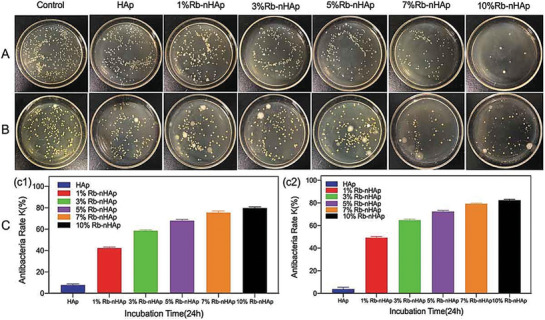
The antibacterial activity of the rubidium‐hydroxyapatite nanocomposite (Rb‐nHAP) was compared with that of pristine hydroxyapatite by bacterial colony tests on surface samples after 24 hours against (A, C1) *Escherichia coli* and (B, C2) *Staphylococcus aureus* (Reproduced with permission.^[^ ^62^ ^]^ Copyright 2020, Taylor & Francis Group).

The development of nanorod‐like rubidium‐hydroxyapatite as a biocompatible and antibacterial implant material was reported for bone tissue engineering applications.^[^ ^62^ ^]^ The incorporation of rubidium into the as‐designed matrix effectively enhanced the proliferation and differentiation capability of human bone cells and showed suitable inhibition properties against both gram‐negative *Escherichia coli* and gram‐positive *Staphylococcus aureus* bacteria (see **Figure** **3**). The applied treatment significantly promoted the proliferation, ALP activity, and early differentiation of the cultured MG‐63 cells into the targeted bone cells, suggesting the multiple functionalities of the rubidium‐based material for antimicrobial therapy applications.

#### 2.2.4 2D Cesium‐Based Nanocomposites

**Figure 4:**
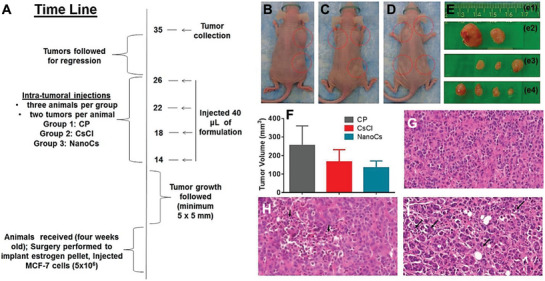
In vivo tumor regression analysis after treatment with cesium‐based biomaterials in mouse xenograft models. The composites were injected into tumors that developed from MCF‐7 cells. (A) Timeline of the experiment performed on animals treated with (B) control; (C) cesium chloride, (D) cesium, and (E) tumor volume images after excision post euthanasia from treated and control animals. (F) Tumor volume regression analysis of the animals. One‐way ANOVA revealed a reduction in the tumor size of the control and nanoccaecal samples (*p *= 0.054). H&E‐stained tumor images after microtomy of waxed tumors from the (G) control, (H) cesium chloride, and (I) cesium‐treated animals. The arrows indicate the approximate locations of the apoptotic‐cell populations with fragmented nuclei (Reproduced with permission.^[^ ^64^ ^]^ Copyright 2016, American Chemical Society Publications).

Cesium is a silvery‐white to silvery‐gold alkali metallic element and is the most electropositive element of the group‐1 family. While the synthesis and application of nanosized crystals for perovskite solar cells (PSCs) has recently been reported, their biological and biomedical applications are largely limited due to their material processability.^[^ ^63^ ^]^ However, in a recent study by Daza et al., the anticancer application of nanocesium particles through the regulation of cesium‐induced metabolic interference was reported. The results suggested that the internalization of cesium cations from this nanocesium compound could limit the glucose internalization process, effectively increasing the intracellular pH to support anticancer properties and subsequent induction of apoptosis in the cells (see **Figure** **4**). The applied treatment consequently resulted in remarkable mitochondrial generation of reactive oxygen species (ROS), resulting in autophagy activation and significant programmed cell death.^[^ ^64^ ^]^ Autophagy and apoptosis are homeostatic mechanisms that regulate inflammation, and their modulation could lead to the development of new therapeutics to treat inflammatory diseases. These findings pave the way for the use of nanocesium‐based biomaterials for future immunoengineering and regenerative nanomedicine, but further research is needed to assess their long‐term safety and therapeutic efficacy.

#### 2.2.5 2D Francium‐Based Nanocomposites

Francium, which is the least electronegative of all the periodic table elements, is estimated to contain less than 30 grams of francium in the Earth's crust at any time. Owing to the rarity and poorly understood physicochemical properties of francium, its biomedical and therapeutic applications as bulk solid crystals or colloids are highly limited. Safety concerns, owing to liability to autoignition in air and severely limited availability, further restrict its use.^[^ ^65^ ^]^ However, a recent study reported the use of francium nanomaterials for the treatment of gum cancer under synchrotron radiation.^[^ ^66^ ^]^ The authors investigated the thermoplasmonic characteristics of simulations of various francium‐based nanostructures that aided in the interaction of nanofrancium with synchrotron radiation emission as a function of beam energy via the finite element method (FEM). Francium nanorods have demonstrated suitable thermoplasmonic characteristics for application in optothermal human cancer treatment by selectively ablating tumor tissues without damaging adjacent healthy tissues. Studies have shown that the high temperature caused at the tumor site by photothermal therapies can cause inflammation at that site, limiting the efficacy of heat‐assisted cancer treatment. For francium nanomaterials to be used in immunoengineering applications, further research is needed to address the limitations posed by inflammation in optothermal treatments and develop inhibition strategies.

Thus, group 1 elements such as lithium, sodium, rubidium, cesium and francium show promise in biomedical applications as 2D nanomaterials from the above‐discussed studies. The adaptation of specialized synthesis techniques to overcome their high reactivity enables the creation of nanomaterials with unique optoelectronic and plasmonic properties for potential immunomodulatory applications.

The group 2 alkaline earth metal elements include beryllium (Be), magnesium (Mg), calcium (Ca), strontium (Sr), barium (Ba), and radium (Ra), which easily lose electrons to become cations, resulting in most of their derived compounds being colorless ionic salts. In addition to radium, elements of this group tend to react with hydrogen, oxygen, and halogens to create metallic hydrides, oxides, and halides, respectively. Although Be‐based nanoforms have suitable physical properties for laser and optoelectronic applications, no obvious studies have been reported on the immunomodulatory effects of these element‐derived layered materials. Beryllium oxide nanofluids and nanostructures synthesized through a facile polymer‒gel method for use in gamma radiation dosimetry^[^ ^67^ ^]^ and as surface‐modified beryllium‐based nanoparticles with polymers possess enhanced stability and dispersibility properties for heat and energy transfer applications.^[^ ^68^ ^]^ Among the group 2 elements, 2D nanoforms of Mg and Ca have been well explored for their antibacterial, immunomodulatory and tissue regeneration potential, especially in hard tissue engineering applications. The ability of the Mg and Ca nanoforms to mimic biological environmental constituents enhances their ability to be used as hard tissue implants without triggering immunogenic responses upon implantation. The following discussions highlight immunoengineered syntheses and applications of these nanoforms, emphasizing their unique biomedical properties.

#### 2.2.6 2D Magnesium‐Based Nanocomposites

Nano magnesium (Mg) materials have gained prominence over beryllium‐based nanocompounds because of the increased availability of Mg resources for a wide range of catalysis, energy storage, and plasmonic and biomedical applications. In particular, the incorporation of low‐dimensional Mg materials into functional composite devices has significantly enhanced their physical and optical properties.^[^ ^69^ ^]^ From a biological perspective, various synthesis methods produce magnesium and magnesium oxide (MgO) nanomaterials with large surface areas, expanding their applications in antimicrobial therapy, biosensors, bioimaging, implant coating, immunomodulation and tissue engineering.^[^ ^70^, ^71^, ^72^, ^73^, ^74^, ^75^ ^]^

**Figure 5:**
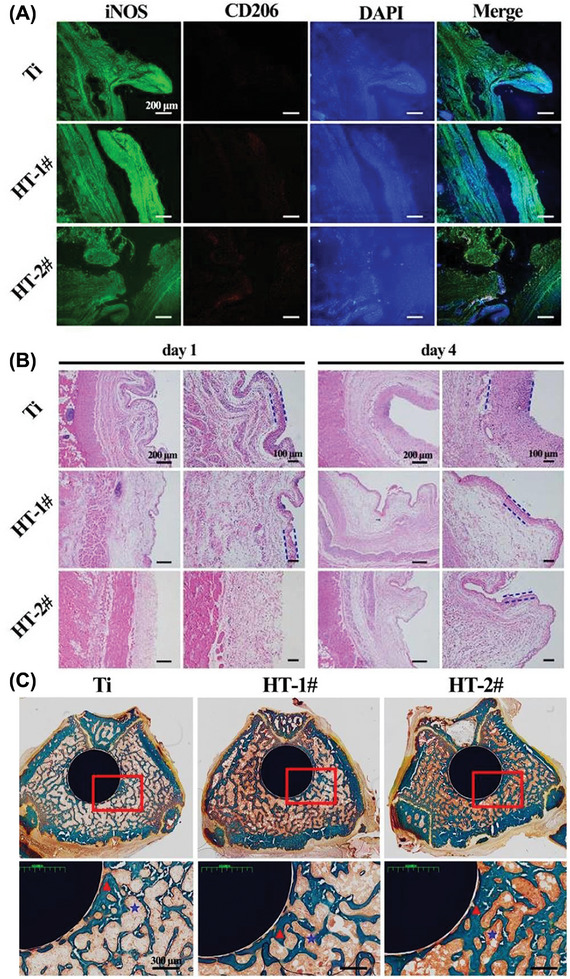
Immunomodulatory characterization of magnesium hydroxide‐modified titanium implants in a tested mouse model. (A) Immunofluorescence imaging of inducible nitric oxide synthase (iNOS) and the mannose receptor cluster of differentiation 206 (CD206) in tissues adjacent to samples. (B) H&E staining; the blue lines indicate the thickness of the fibrous layers. (C) Representative methamphetamine blue‐stained images of the femur bone after implantation for eight weeks (Reproduced with permission.^[^ ^71^ ^]^ Copyright 2022, Oxford University Press).

An exploration of the immunomodulatory effects of magnesium‐based composites in vitro and in vivo^[^ ^71^ ^]^ revealed that magnesium hydroxide (MgOH) nanosheets synthesized as highly bioactive films can be used to coat titanium (Ti)‐based implants. The nanotopography and sustainable Mg ion release from the MgOH biofilm surface are crucial for modulating the immunomodulatory functions of implants by regulating the polarization of macrophages to the macrophage‐2 (M2) phenotype and promoting tissue healing. Macrophage‐conditioned culture media collected from the nano‐MgOH‐coated Ti samples treated with BMSCs from human umbilical vein endothelial cells (HUVECs) showed superior osteogenic and angiogenic activities. As shown in **Figure** **5**, the reinforcement of the bare Ti implant with nano‐MgOH films effectively reduced local inflammation by reducing inflammatory cell infiltration and creating thinner fibrous layers in the tested animals than in the control groups did (Figure 5 panels A, B), with an increase in closely adhered bone formation on the modified implants and surrounding regions (Figure 5 panel C), suggesting the promotion of osteointegration. This study revealed that nano‐MgOH coated on Ti implants is a promising candidate for immunomodulatory orthopedic applications.

Nanomagnesium has gained attention for its ability to sustain localized surface plasmon resonances across different ultraviolet, visible, and near‐infrared wavelengths. These plasmonic properties can be tuned by manipulating their size and shape, which is beneficial for their application in fluorescence‐based biomonitoring and/or light‐activated cancer therapy.^[^ ^76^ ^]^ Truskewycz et al. developed fluorescent MgOH‐based nanosheets (NSs) with tailored bioactivity properties for antimicrobial wound dressings and pH‐tracking therapeutic applications.^[^ ^77^ ^]^ The authors tested these nanosheets against different bacterial species on solid surfaces via the typical alkaline precipitation method. The autofluorescence property of the MgOH‐NSs was attributed to their hydroxide constituents, which led to pH‐responsive behavior that allowed them to heal. In addition, strong fluorescence signals are emitted to track the degradation rate and antimicrobial functionality of the material. In addition to their antimicrobial and plasmonic properties, MgOH‐NSs possess excellent anti‐inflammatory properties by reducing the production of inflammatory macrophages and enhancing chronic wound healing. The destruction of microorganisms increases inflammation and the anti‐inflammatory property of Mg, reducing inflammation at the wounded site and creating an acidic environment. This change in pH causes the mineralization of MgOH to Mg^2+^, which contributes to the proliferation of keratinocytes and the migration of fibroblasts to improve their health. Furthermore, its pH‐sensitive profile could be used effectively as a pH‐responsive probe for detecting wound acidification and accelerating the wound healing process upon skin injury. Thus, when MgOH‐NSs are integrated into electrospun wound dressings, they promote keratinocyte proliferation and fibroblast migration for healing and visually indicate health by indicating an acidic environment. In addition, the planar 2D morphology of MgOH‐NSs provides a high specific surface area and alkaline environment to enhance interactions with microorganisms to improve antimicrobial properties.

**Figure 6:**
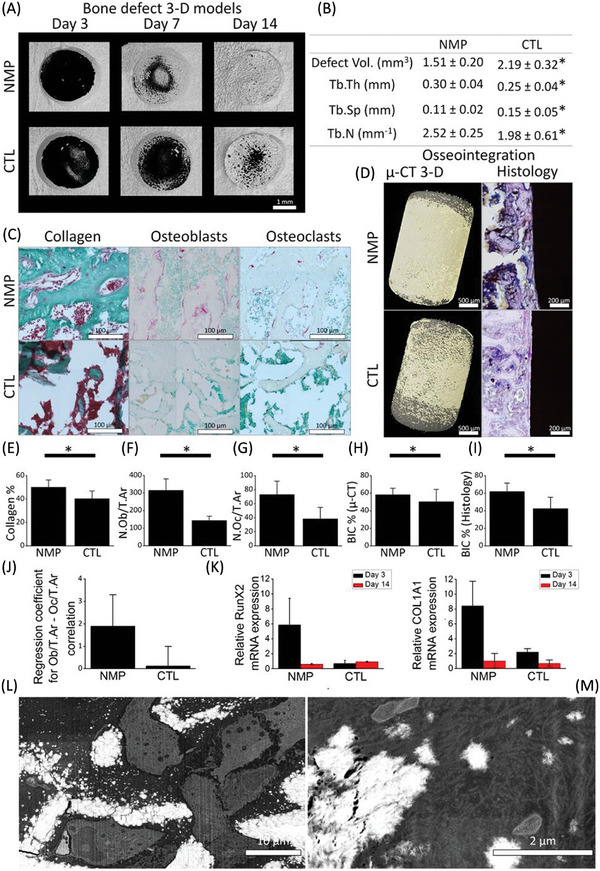
Demonstration of the effect of nanocrystalline magnesium phosphate (NMP) on accelerating bone healing and implant osseointegration processes. (A, B) 3D computerized microtomography models and corresponding analysis of the bone defects at days 3, 7 and 14 revealed a remarkable improvement in bone healing among the NMP‐treated defects. (C) μ‐CT analyses revealed a smaller defect volume, less trabecular separation, greater trabecular thickness and greater number of trabeculae in the NMP‐treated samples than in the control samples. (D) Masson's trichrome staining revealed more collagen formation (E), alkaline phosphatase staining revealed more osteoblasts (F), and tartrate‐resistant acid phosphatase staining revealed more osteoclasts (G) in the NMP‐treated defects. (D, H, I) Computerized microtomograph models and coronal histological sections of titanium‐based implants show more bone in contact with the artificial substrate than do magnesium phosphate‐based nanocomposite‐coated samples. (J) Regression coefficients for the NMP samples. (K) qRT‒PCR analysis revealed that the expression of RunX2 and COL1A1 was upregulated in the nanotreated samples compared with the control samples on day 3. However, no significant differences were observed on day 14. FIB images depicting the bone matrix (L) and the collagen fibers (M) undergoing mineralization by osteoblasts in the treated samples on day 7. Data analyses are assessed by two‐sample‐student calculations (t tests) and accepted as statistically significant (*) at *p* values of less than 0.05 (Reproduced with permission.^[^ ^78^ ^]^ Copyright 2016, American Chemical Society Publications).

Mg‐based nanomaterials are gaining importance in tissue engineering and regenerative medicine applications. A study by Laurenti et al. described the synthesis of a new crystalline complex nanomaterial containing magnesium phosphate nanosheets (Mg_3_(PO_4_)_2_‐NS) for bone regeneration.^[^ ^78^ ^]^ Bone comprises 50% of the body's magnesium and plays a role in mineralization, leading to enhanced calcification, osteoblast proliferation and differentiation, which was exploited in this study to develop a 2D nanomaterial‐incorporated hydrogel, which offers an innovative approach to bone tissue engineering, replacing conventional bioceramics. The incorporation of sodium (Na) in Mg_3_(PO_4_)_2_‐NS improved the crystallization and 2D planar morphology of the matrix. The resulting biomaterial possesses good processability for incorporation into polymeric hydrogels, good biocompatibility, injectability for biomedical tissue engineering applications, and desirable biological properties to accelerate osteogenesis and osseointegration processes in vitro and in vivo. The Mg_3_(PO_4_)_2_‐NS hydrogels showed enhanced biocompatibility with human fibroblasts and enhanced bioactivity through upregulated expression of bone formation‐associated genes, including collagen type I alpha 1 (COL1A1), runt‐related transcription factor 2 (RunX2), alkaline phosphatase (ALP), osteocalcin (OCN), and osteopontin (OPN), in mouse bone marrow cells (see **Figure** **6**). Elsewhere, Turan et al. reported the study of different nanostructured ceramics such as MgO and niobium oxide (Nb_2_O_5_) and how tailoring their physical, mechanical and bioactivity properties aids in hard tissue repair. Hydroxyapatite is the traditional ceramic used for hard tissue engineering, and incorporating nanostructures into these materials has been reported to increase surface properties. Hence, this study discusses the importance of developing nanostructured hydroxyapatite (HAP) containing metallic oxide nanoparticles, MgO and Nb_2_O_5_ to enhance the surface properties of HAP. Thus, the improved bone implant substrates showed greater mechanical durability while retaining their bioactivity and without inducing any immunologic response.^[^ ^79^ ^]^

Furthermore, a new design of 2D magnesium‐based nanosheets was reported by Fan et al. as a hydrogen‐releasing and acid‐responsive prodrug for synergistic gastric cancer chemotherapy.^[^ ^80^ ^]^ Briefly, the synthesized biocomposites were used for hydrogeno‐chemotherapy via the encapsulation of magnesium‐boron (MgB) nanosheets (NSs) in polyvinylpyrrolidone (PVP) pills and were administered intravenously with doxorubicin. The bioengineered pills demonstrated high compatibility, stability in biological environments, sustained gastric acid responsiveness and suitable bioactivity properties. MgB‐NS‐PVP acts as a hydrogen prodrug and combines with doxorubicin, and the chemotherapeutic drug creates a synergistic hydrogeno‐chemotherapeutic agent. This specific hydrogeno‐chemotherapy treatment markedly reduced the side effects on normal cells; prolonged the viability of gastric cells; selectively inhibited the aerobic respiration of gastric cancer cells; and activated aerobic respiration in normal cells, such as mesenchymal stem cells and hepatic, splenic, and cardiac cells with high proliferation, to ensure the protective role of the nanomaterial in gastric cancer.

Together, these findings support the future of layered magnesium‐based nanomaterials in potential biomedical, immunomodulatory, tissue engineering and chemotherapeutic applications.

#### 2.2.7 2D Calcium‐Based Nanocomposites

The structures of calcium (Ca) have been largely investigated for use in biomedical applications such as dental implants, bone tissue engineering and immunomodulation‐based applications. Calcium ions (Ca^2+^) and phosphorous ions (PO_4_^3−^) are present in the body, are important constituents of the bone matrix and present themselves as calcium phosphate (Ca_3_(PO_4_)_2_. Ca^2+^ induces bone formation, stimulates osteoblasts toward bone maturation and regulates osteoclast resorption. PO_4_^3−^ regulates osteoblast differentiation and bone tissue formation. Thus, synthetic Ca_3_(PO_4_)_2_ is employed in research because of its prolonged bone regenerative characteristics, mimicking the natural role of Ca^2+^ and PO_4_^3−^ in bone metabolism for bone repair. Owing to its enhanced bioactivity and antimicrobial properties, nanoCa_3_(PO_4_)_2_ has been studied for bone‐like implant applications.^[^ ^81^ ^]^ Saglam et al. reported that antibiotics containing nanomaterials offer suitable antimicrobial and biodegradation properties to enhance the osseointegration process and prevent implant failure. The authors proposed that calcium‐based nanomaterials form bioactive films on implant surfaces, preventing bacterial colonization and regulating anti‐inflammatory responses locally and systemically. In fact, multifunctional HAP‐ in the form of nano Ca_3_(PO_4_)_2_ coatings enables desirable osteoconductive and drug delivery properties, promoting bone healing and implant durability. Furthermore, these bioactive nanomaterials intrinsically dissociate into ions and enhance their cellular internalization, delivery of therapeutic molecules and regulation of immunomodulatory gene expression to significantly reduce the risk of inflammation and implant rejection. In addition, the surface texture of these nanomaterials critically influences the crosstalk between host inflammatory cells and regenerative cells, stimulating osteoinduction. Thus, nanocalcium‐based substrates modulate the inflammatory response and improve the potential for bone regeneration.

Mitwalli et al recently developed nanocalcium‐fluoride (CaF_2_) as a bioactive dental composite with improved remineralization and antibacterial properties.^[^ ^82^ ^]^ At the onset of tooth caries, the success rate of tooth caries restoration is limited because acidic biofilms cause inflammation and increased susceptibility to recurrent bacterial infections, limiting restoration success. Repeated treatments to address this issue lead to the loss of tooth structure. Understanding this crucial situation, formulating nanobased dental composites with improved antibacterial properties to target biofilm formation to inhibit enamel demineralization and offer long‐term protection against recurrent caries is vital. The long‐term release of F and Ca from the CaF_2_ nanocomposite enhanced remineralization and prevented demineralization by suppressing bacterial growth. Together, these findings highlight the potential of calcium‐based nanocompounds as antimicrobial, antibacterial, immunomodulatory, and hard tissue engineering candidates for regenerative medicine.

### 2.3 Group 3 to Group 12 Elements and Reported Immunoengineering Applications

Most of the elements in groups 3 to 12 are transition metal elements that are widely used as ultrasmall biomaterials in different nanoforms, ranging from nanoparticles to nanosheets to nanocomposites to quantum dots. These elements are formulated as 2D transition metal carbides and nitrides (MXenes), transition‐metal dichalcogenides (TMDs), transition‐metal oxides (TMOs), metal‒organic frameworks (MOFs), metal‒organic layers (MOLs) and layered double hydroxides (LDHs). The different forms of these two‐dimensional nanomaterials are comprehensively discussed below.

Briefly, layered MXene nanosheets are composed of transition metal carbides/nitrides that are exfoliated from their parent “MAX” phase. The “M” represents the transition metal elements from the periodic table, and most reported MXenes are from the metallic elements of group‐4 to gr. The “A” layer represents the elements from Groups 13, 14, and 15, and “X” represents its carbide or nitride. The general structural formula of MXenes is “M_(n+1)_X_n_T_x_”, where the T_x_ represents the surface terminations on the MXenes that are obtained due to the exfoliation process.^[^ ^83^, ^84^, ^85^ ^]^ Since its discovery in 2011, MXenes have been used in a wide variety of applications, and owing to their hydrophilicity and biocompatibility, MXenes have been applied in the biomedical and clinical fields. MXenes are used in tissue engineering, cancer therapy, such as chemotherapy, anticancer drug delivery, photodynamic/thermal therapy, radiation therapy, and diagnosis, as in magnetic resonance imaging (MRI), photoacoustic imaging (PAI), computed tomography (CT) imaging, biosensing, immunomodulators, and drug delivery applications.^[^ ^86^, ^87^, ^88^, ^89^, ^90^, ^91^ ^]^

TMDs are 2D nanomaterials that can be used as alternatives to graphene. The TMD structural formula is X‐M‐X, where M is the transition metal from groups 4–7 from the periodic table (Mn, Mo, Ta, W, Re) and X is the chalcogenide (S, Se, Te). The structural formula indicates that M is sandwiched between two chalcogenides X. The most widely explored TMD because of its immunomodulatory effects is molybdenum disulfide (MoS_2_). Other forms of TMDs, such as molybdenum diselenide (MoSe_2_), tungsten disulphide (WS_2_), tungsten diselenide (WSe_2_), and molybdenum ditelluride (MoTe_2_), are synthesized by similar exfoliation techniques. WSe_2_ and MoSe_2_ have been applied as photosensors, phototransistors, nanobiosensors and nanocarriers, whereas WS_2_ is used in light‒matter interaction applications because of its optoelectronic properties.^[^ ^92^, ^93^ ^]^

TMOs are among the most extensively studied 2D nanomaterials because of their remarkable chemical and physical properties. The unique nature of d‐electrons in transition metals results in multiple oxidation states for the cations, which strongly influences their electrical properties. As a result, TMOs possess enhanced electron mobility, ductility, stability and biodegradability, making them biocompatible for biomedical applications. TMOs have been shown to increase the level of ROS in the tumor microenvironment, facilitating their application in synergistic photodynamic, photothermal, magnetic, thermal, sonodynamic and cancer therapies.^[^ ^94^ ^]^ The oxidative properties of TMOs, such as titanium oxides, manganese dioxides, and zinc oxides, induce oxidative stress in tumor cells. This leads to the release of tumor‐associated antigens, which in turn activate dendritic cells and enhance antigen presentation, ultimately facilitating tumor cell elimination. TMOs such as iron oxides and tungsten oxides extend their immunomodulatory properties by recruiting macrophages and inducing macrophage polarization, thus leading to the inhibition of tumor growth in cancer immunotherapy applications.^[^ ^95^ ^]^

Another class of widely studied 2D nanomaterials is MOFs, which are known for their highly tunable structural and functional chemistry. MOFs are composed of metal‐containing nodes and organic linkers and are widely used in energy transfer, photocatalysis and biomedical applications.^[^ ^96^ ^]^ The photocatalytic properties of MOFs have been harnessed to develop nanoplatforms for photoimmunotherapy in cancer treatment.^[^ ^97^, ^98^ ^]^ Additionally, MOFs have also been utilized as substrates for the immobilization of cytokines to provide sustained cytokine presentation.^[^ ^99^ ^]^ Notably, MOFs exhibit dose‐dependent radiosensitization properties that can be utilized to modulate the ROS levels and DNA damage caused by radiation therapy in cancer treatment.^[^ ^100^ ^]^ MOLs are 2D counterparts of MOFs with thicknesses in the nanometer range. Compared with that of MOFs, the increased number of accessible active sites in MOLs has been found to be advantageous in facilitating easier chemical and surface modifications.^[^ ^101^ ^]^ This ability of MOLs enables enhanced tailoring of their molecular and material chemistry, making them suitable candidates for electro and photocatalysis‐based applications. Interestingly, in some instances of ration‐based cancer therapies, nanosized MOLs have shown superior performance compared with MOFs.^[^ ^102^ ^]^

Another promising class of versatile 2D nanomaterials with significant biomedical applications is LDH. The unique lamellar structure of these materials, consisting of positively charged metal hydroxide layers and intercalated anions, contributes to their highly tunable structural and functional properties, leading to desirable biomedical outcomes.^[^ ^103^ ^]^ Research shows that LDHs are immunoengineered, leveraging their unique properties to enhance their therapeutic efficacy in cancer therapy.^[^ ^104^ ^]^ For example, altering the crystalline structure of LDHs has significantly improved their sonodynamic cancer therapeutic potential by increasing the generation of reactive oxygen species to effectively destroy tumor cells.^[^ ^105^ ^]^ Furthermore, photosensitizer‐functionalized LDHs offer a targeted approach to treat cancer through a light‐activated mechanism involving singlet oxygen generation.^[^ ^106^ ^]^ By incorporating photoactive components, LDHs can be tailored to release therapeutic agents to activate immune responses upon light stimulation.^[^ ^107^, ^108^ ^]^

The following section discusses the reported immunomodulatory roles and mechanisms of these layered transition metal‐based biomaterials in a group‐by‐group and element‐by‐element order.

#### 2.3.1 Group 3 Elements and Reported Immunoengineering Applications

The group‐3 elements constitute the first group of transition elements and include scandium (Sc), yttrium (Y), and lanthanium (La), which are classified with lanthanide elements, and actinium (Ac), which is classified with actinoids. Elements of Group 3 have no biological role, and to the best of our knowledge, no obvious studies have reported the immunomodulatory effects of these aforementioned elements in the literature, except for cerium.

##### 2.3.1.1 Cerium‐Based Nanocomposites

**Figure 7:**
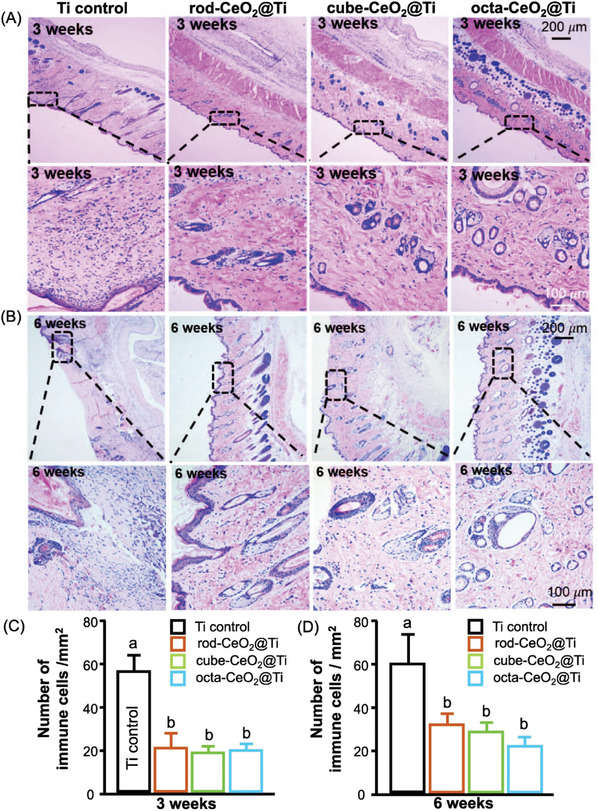
In vivo evaluation of inflammation in the tissues around titanium‐ and titanium surface‐modified implants with rods, cubes, and octa‐shaped nanoceria (CeO_2_@Ti). (A, B) H&E staining after 3 and 6 weeks in the experiments. (C, D) Quantification of immune cells in 72 areas in the surrounding tissues of 24 rats in each group (significantly different from each other: *p* < 0.05) (Reproduced with permission.^[^ ^116^ ^]^ Copyright 2019, Elsevier Ltd.).

**Figure 8:**
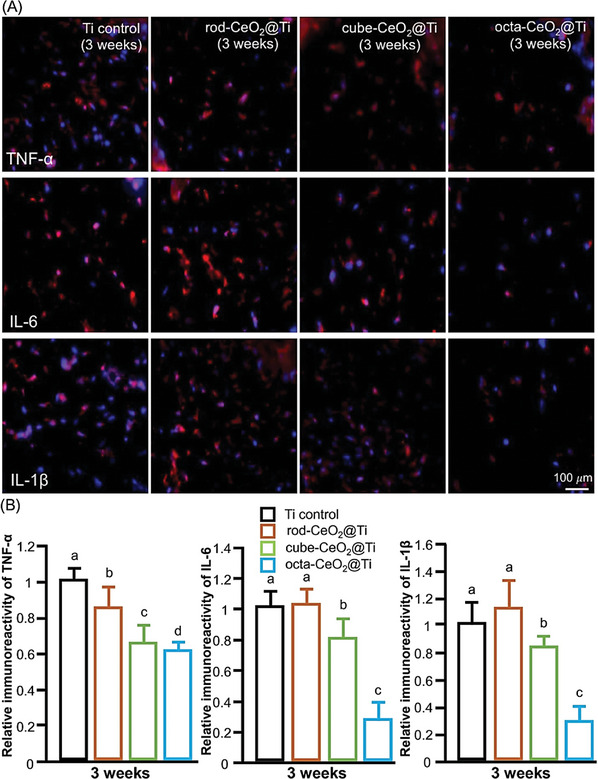
(A) Immunofluorescence H&E staining images of inflammatory cytokines, including IL‐6, IL‐1β, and tumor necrosis factor‐alpha (TNF‐α), in the areas surrounding the implanted titanium substrates and rods, cubes, and octa‐shaped CeO_2_‐coated titanium disks after 3 weeks. (B) Relative intensity of fluorescence detection of these inflammatory cytokines around the control and experimental implants after 3 weeks (significantly different from each group: *p* < 0.05) (Reproduced with permission.^[^ ^116^ ^]^ Copyright 2019, Elsevier Ltd.).

Among the 14 known lanthanoids, cerium (Ce)‐based 2D materials have been found to possess immunomodulatory properties. Cerium derivatives are widely used in wound healing remedies because of their antimicrobial properties, making them valuable in biological and clinical applications.^[^ ^109^ ^]^ For example, cerium nitrate is used as an antiseptic to treat deep burns and in combination with silver sulfadiazine as a clinically accepted wound treatment.^[^ ^110^, ^111^ ^]^ In addition to its antimicrobial effects, it also elicits immunomodulatory effects, preventing sepsis in patients suffering severe burn injuries. Preservation of T‐cell functions and regulation of interleukin 2 (IL‐2) production.^[^ ^112^ ^]^ The antimicrobial properties of cerium oxide are mediated through oxidative stress, especially in its low‐dimensional form, such as nanoceria/cerium oxide nanoparticles (CeO_2_‐NPs), and aid in wound healing applications.^[^ ^113^, ^114^ ^]^ As cerium has properties similar to those of calcium and in combination with its antimicrobial and antioxidant properties, it is used in bone tissue engineering. A recent study by Li et al reported the antibacterial and anti‐inflammatory applications of CeO_2_‐NPs for surface modification of titanium implants.^[^ ^115^, ^116^ ^]^ These results indicate that CeO_2_‐NP‐modified surfaces have greater anti‐inflammatory and ROS‐scavenging activities than control *surfaces do in vitro*. Their in vivo data revealed significant decreases in the mean mRNA expression of IL‐6, IL‐1b, and TNF‐a after treatment with different rods, cubes, and octa‐shaped CeO_2_ coatings. As illustrated in **Figures** **7** and **8**, histomorphometric analysis revealed a significant reduction in inflammation around CeO_2_‐_NP‐_modified implants compared with the control, and this reduction, estimated by measuring the number of inflammatory cells present, demonstrated the effectiveness of CeO_2_‐NPs in mitigating the inflammatory response.

Furthermore, cerium‐hydroxyapatite (Ce‐HAP)‐based nanomaterials are used as antimicrobial bone implants for directing bone marrow mesenchymal stem cells toward the osteogenic lineage and relieving inflammatory responses via ROS scavenging.^[^ ^117^ ^]^ When doped into different glass, collagen, and chitosan scaffolds, these CeO2 materials show good biocompatibility with BM‐MSCs, supporting their viability and proliferation for enhanced osteointegration in bone tissue engineering applications.

Owing to the ability of CeO_2_‐NPs to mimic redox enzymes, they can be used in immunomodulatory cancer therapy applications by extending their enzyme‐like activity in redox regulation.^[^ ^118^, ^119^, ^120^ ^]^ Their ability to cycle between the Ce^3+^ and Ce^4+^ oxidation states and the presence of oxygen vacancies on the CeO_2_ surface with a Ce core, which leads to enzymatic ROS scavenging, is the foundation for the use of CeO_2_‐NPs in cancer therapy.^[^ ^121^ ^]^ The antioxidant properties, good biocompatibility, ability to reduce drug resistance, and high chemical stability are other alluring properties of CeO_2_‐NPs for use in biomedical applications. Another attractive feature is their ability to exhibit differential cytotoxicity, as they act as cytotoxic agents in acidic cancer environments while being protective agents (antioxidants) for normal cells at neutral pH.^[^ ^122^ ^]^ Under hypoxic conditions, CeO_2_‐NPs trigger angiogenesis and catalytically regulate intracellular oxygen, leading to the stabilization of hypoxia.^[^ ^123^ ^]^ The high surface area combined with the ratio of the two oxidation states of cerium (Ce^3+^/Ce^4+^ ratio) also regulates the level of intracellular oxygen, leading to robust induction of angiogenesis.^[^ ^124^ ^]^ CeO_2_‐NPs have been reported to be valuable nanozymes in cancer diagnosis as immune^[^ ^125^, ^126^, ^127^, ^128^ ^]^/DNA^[^ ^129^, ^130^ ^]^ electrochemical biosensors for tumor marker detection. CeO_2_‐NPs have been designed for magnetic resonance contrast imaging technologies for cancer detection.^[^ ^131^ ^]^ The ability of CeO_2_‐NPs to regulate ROS helps establish their role in photodynamic therapy (PDT) and photothermal therapy (PTT).^[^ ^132^ ^]^ PTT utilizes the ability of photosensitizers (PSs) to be stimulated by light to produce heat, leading to the generation of ROS, followed by the induction of oxidative stress at the tumor site to kill tumor cells. In the tumor environment, the large surface area, selective targeting mechanisms and increased circulation time of light‐stimulated CeO_2_‐NPs help increase the accumulation of PS and effective ROS generation to disrupt tumor cells.^[^ ^133^, ^134^ ^]^ Owing to its cytotoxic and protective antioxidant properties, CeO_2_‐NPs are used in radiotherapy^[^ ^135^, ^136^, ^137^, ^138^ ^]^ and chemotherapy^[^ ^139^, ^140^ ^]^ to synergistically protect the normal cells and selectively target the tumor cells. The use of CeO_2_‐NPs in PDT, PTT, radiotherapy and chemotherapy has potential for combination therapy approaches. Their ability to sensitize cancer cells enhances radiation/chemotherapy‐induced cell death in combination therapy, such as PDT/PTT,^[^ ^141^, ^142^ ^]^ and in chemotherapy/PTT,^[^ ^143^, ^144^ ^]^ demonstrating their versatile, multimodal, immunomodulatory, antioxidant application in cancer theranostics.^[^ ^145^ ^]^

Overproduction of stress‐induced ROS causes oxidative stress in distressed tissue, leading to dysregulation of the immune system. CeO_2_‐NPs effectively scavenge overproduced ROS, modulate the immune response and protect tissue from oxidative damage.^[^ ^146^ ^]^ A comparative study conducted on two catalytic nanoparticles, TiO_2_ and CeO_2_, revealed that both promote immunomodulation via dendritic cells (DCs) and T‐helper (T_H_) cells. This study revealed that CeO_2_‐NPs targeted antigen‐presenting cells (APCs) and stimulated DCs to secrete the anti‐inflammatory cytokine IL‐10. When cocultured with allogenic CD4^+^ T cells, CeO_2_‐NPs triggered the expression of T_H_2 regulatory cytokines (IL‐4, IL‐5 and IL‐10) and provided a basis for modulating immunity via the use of metallic nanoparticles.^[^ ^147^ ^]^ Cubic and sphere‐shaped CeO2‐NPs have the potential to modulate reactive nitrogen species (ROS) and reactive nitrogen species (RNS) and cause immune cell induction in respiratory syncytial virus (RSV) disease by modulating RSV‐infected alveolar macrophage polarization and increasing CD80, CD86, TNF‐α, and IL‐12p70 expression. The CeO_2_‐NPs decreased M2 CD206 expression and modulated the innate cellular response to address infection.^[^ ^148^ ^]^ Oxidative stress leads to the release of proinflammatory cytokines, and CeO_2_‐NPs have shown promise in treating chronic pain syndrome (UCPPS) via ROS scavenging, reducing oxidative stress and, in turn, downregulating the proinflammatory cytokines IL‐6 and TNF‐α.^[^ ^149^ ^]^ Polyethylene glycol (PEGylated) CeO_2_‐NPs have been developed to treat COVID‐19, leveraging their antimicrobial, antiviral, antioxidant and immunomodulatory properties. COVID‐19 suppresses cytokine expression, triggers ROS, and leads to multiple organ failure. During these events, CeO_2_‐NPs increase superoxide dismutase (SOD) and catalase (CAT) levels, reducing the degree of oxidative damage caused by ROS generation. Additionally, they exhibit immunostimulatory properties by activating the T‐cell‐mediated immune response, suppressing inflammation and preventing multiple organ damage, suggesting their potential as therapeutic agents for COVID‐19.^[^ ^150^ ^]^

CeO_2_‐NPs act as immunomodulators by increasing their antiapoptotic effect via the scavenging of free radicals in neurological disorders such as Parkinson's disease and Alzheimer's disease. These compounds exert their anti‐inflammatory effects by reducing hydrogen peroxide (H_2_O_2_) levels under ischemic and hemorrhagic stroke conditions. The reduction in free radicals is elicited by the ability of the NPs to mimic SOD, revealing the neuroprotective role of CeO_2_‐NPs through the conversion of superoxide anions to hydrogen peroxide and oxygen.^[^ ^151^ ^]^ Parkinson's disease is caused by the accumulation of alpha‐synuclein (α‐synuclein) and by a mutation in the α‐synuclein gene SNCA, leading to ROS generation and neuroinflammation and causing microglial cell neurotoxicity.^[^ ^152^ ^]^ CeO_2_‐NPs scavenge excess ROS, interact with α‐synuclein to inhibit its accumulation,^[^ ^153^ ^]^ protect dopaminergic neurons and preserve dopamine levels in an induced Parkinson's disease mouse model.^[^ ^154^, ^155^ ^]^ Hegazy MA et al. emphasized the need for dose optimization of CeO_2_‐NPs for effective neuroprotection from Parkinson's disease.^[^ ^156^ ^]^ Europium‐doped CeO_2_‐NPs (CeO_2_‐NPs‐Eu) exhibit an immunomodulatory effect in treating the neurodegenerative disorder Alzheimer's disease (AD) by maintaining immune cell homeostasis, targeting amyloid beta (Aβ) plaques that accumulate in diseased conditions, oxidative stress, and inflammation and inducing protective intracellular signaling. Microglia are special macrophage‐like cells that orchestrate the inflammatory response in the central nervous system. Eu‐CeO_2_‐NPs target microglia, increase microglial phagocytosis, upregulate the expression of CD36 scavenger receptors to facilitate Aβ clearance, attenuate the expression of proinflammatory cytokines (IL‐6, IL‐1β, and TNF‐α) and restore homeostasis in the diseased environment.^[^ ^157^ ^]^ PEGylated CeO_2_‐NPs (CeNPs‐PEG) decreased the expression of the proinflammatory M1 biomarker CD16/32 and increased the expression of the anti‐inflammatory M2 biomarker CD206 via intracellular ROS scavenging in microglia in vivo. CeNPs‐PEG eliminate ROS and lead to a reduction in the translocation of P65 NFκB induced by the inhibition of ROS‐triggered inflammation. CeNPs‐PEG shifted the microglial phenotype from the M1 phenotype to the M2 phenotype, which led to the control of the NFκB pathway via elimination of ROS.^[^ ^158^ ^]^

CeO_2_‐NPs play a catalase‐mimicking role in ischemic/hemorrhagic stroke conditions via the reduction of H_2_O_2_ into oxygen and water, leading to ROS scavenging. During ischemic stroke, disruption of the cerebral blood supply and central nervous system (CNS) infarction and the restriction of oxygen lead to acidosis in neurons. This condition induces oxidative stress via the generation of reactive oxygen species (ROS) and additional stress induced by H2O2 radicals and superoxide anions.^[^ ^159^ ^]^ CeO_2_‐NPs target H2O2 radicals and superoxide anions and scavenge ROS, which efficiently reduces ischemic cell death in the brain.^[^ ^160^ ^]^ The cytoprotective role of CeO_2_‐NPs in efficiently removing ROS and acting against RNS^[^ ^161^ ^]^ shows their therapeutic potential for treating ischemia‒reperfusion injury. Recent studies using CeNPs‐PEG^[^ ^162^ ^]^ and RGD‐CeNPs^[^ ^163^ ^]^ have shown that the repair of ischemia‐induced cerebral damage by ROS scavenging provides potentially new treatments for ischemic stroke. Hemorrhagic stroke is caused by rupture of the cerebral vasculature, accounting for its subtypes of intracerebral hemorrhage characterized by nontraumatic parenchyma bleeding^[^ ^164^ ^]^ and subarachnoid hemorrhage characterized by bleeding by aneurysmal bleeding.^[^ ^165^ ^]^ To reinstate homeostasis, thrombin is secreted, which induces an inflammatory response with additional microglial activation accelerating ROS generation in the hemorrhagic region.^[^ ^166^ ^]^ The phospholipid CeNPs‐PEG were found to aid in relieving oxidative stress by scavenging ROS in intracerebral hemorrhage via the reduction of CD68^+^ microglia/macrophages.^[^ ^167^ ^]^ Additionally, in subarachnoid hemorrhage, oxyhemoglobin is overexpressed and leads to ROS production‐induced oxidative stress.^[^ ^168^ ^]^ Water‐soluble aminocaproic acid‐coated CeO_2_‐NPs were synthesized to address the oxidative stress that occurs during subarachnoid hemorrhage, as they have the ability to remove superoxide anions and hydroxyl radicals, leading to ROS generation.^[^ ^169^ ^]^ The remarkable antioxidant properties of CeO_2_‐NPs promote free radical scavenging in various acute and chronic disease conditions, indicating their potential in the treatment of chronic inflammation. They modulate immunosuppression and immunostimulation due to their anti‐inflammatory properties. CeO_2_‐NPs, as immunomodulators, serve as vaccine adjuvants and antiallergy therapeutics,^[^ ^170^, ^171^, ^172^ ^]^ highlighting the need to explore NP‐mediated nanoimmunointeractions for clinical applications.

**Figure 9:**
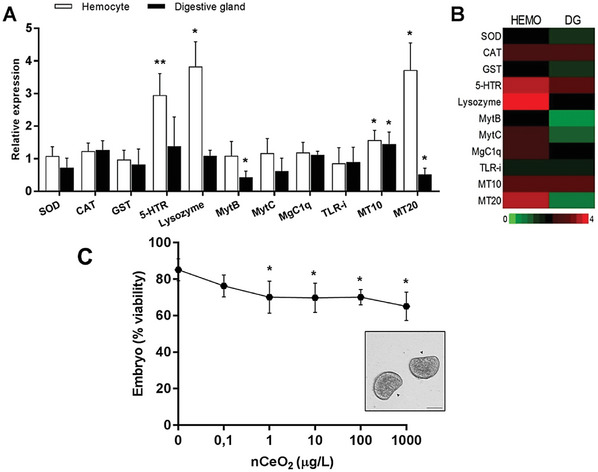
Effects of exposure to 100 µg/L CeO2 for 96 h on gene transcription in *Mytilus galloprovincialis* hemocytes (white bars) and digestive glands (black bars). (A) Relative expression of superoxide dismutase, catalase, glutathione transferase, 5‐hydroxyl triptamine receptor, lysozyme, mytilin, myticin B, C1q‐domain‐containing protein, Toll‐like receptor i isoform, and metallothionein isoforms 10 and 20. (B) Heatmap of differentially expressed genes in each sample (n = 6, *p < 0.05, and (Mann‒Whitney U test)). (C) Effects of CeO_2_ on *M. galloprovincialis* larval development after 48 h in a tested embryotoxicity bioassay. The fertilized eggs were exposed to CeO_2_ at different doses ranging from 0.01 to 1000 µg^/L^. (n = 6 for each sample). Inset: Representative light microscopy image of CeO_2_‐exposed embryos at a concentration of 1000 µg/L at a scale of 20 µm (Reproduced with permission.^[^ ^179^ ^]^ Copyright 2019, Elsevier Inc.).

Furthermore, CeO_2_‐NPs were found to demonstrate complex immunomodulatory effects, including both protective and potentially harmful outcomes. These cells play a protective role by activating mast cells that promote pulmonary inflammation, impair vascular relaxation and aggravate myocardial ischemia‒reperfusion injury. The pulmonary instillation of CeO_2_‐NPs led to increased production of prostaglandin D2 (PGD_2_), TNF‐α, IL‐6 and osteopontin as a cardiovascular inflammatory response.^[^ ^173^ ^]^ While CeO_2_‐NPs provide cardioprotection by increasing osteopontin, which is involved in cardiovascular remodeling and the pathogenesis of inflammatory/immune diseases, CeO_2_‐NPs can cause lung toxicity and inflammation when exposed to diesel engine exhaust, which uses cerium as a catalyst. CeO_2_‐NPs induce lung inflammation by increasing IL‐12 and osteopontin levels and reducing NO levels, which can lead to fibrosis in the lungs.^[^ ^174^ ^]^ In a dose‐ and time‐dependent manner, CeO_2_‐NPs increased the levels of IL‐1β and MIP‐2, which are mediators of acute neutrophilic inflammation.^[^ ^175^ ^]^ Prolonged exposure to CeO_2_‐NPs in rodent models increased the concentrations of the proinflammatory cytokines IL‐1β, TNF‐α, and IL‐6, leading to oxidative stress‐induced lung toxicity and impaired clearance and chronic inflammatory response.^[^ ^176^ ^]^ They can also trigger delayed‐type hypersensitivity via the induction of the inflammatory cytokines IFN‐γ and IL‐10, leading to lung fibrosis in mice^[^ ^177^ ^]^ and being immunotoxic to human monocytic THP‐1 cells.^[^ ^178^ ^]^ In addition, CeO_2_‐NPs exhibit immunogenic properties and potential toxicity in aquatic environments. CeO_2_‐NP exposure in mussels led to increased expression of immune and antioxidant genes at the molecular level; increased ROS and lysosome release at the cellular level; reduced embryonic development at the tissue level; and the induction of the oxidative stress genes catalase (CAT) and glutathione‐s‐transferase (GST) in the hemocytes and digestive glands of mussels.^[^ ^179^, ^180^ ^]^ This shows the selective immunomodulatory properties demonstrated by cerium nanoparticles at different levels of biological organization (see **Figure** **9**).

These reports highlight the need to carefully study the nanoimmune responses evoked by the synthesized CeO_2_‐NPs for biological use and as hazardous materials from industrial sources into the environment, as each exhibits varying toxicity and inflammatory effects.

#### 2.3.2 Group‐4 Elements and Reported Immunoengineering Applications

Group 4 is the second group of transition metals in the periodic table of chemical elements. It contains four elements: titanium (Ti), zirconium (Zr), hafnium (Hf), and rutherfordium (Rf). With the exception of rutherfordium, all the other elements have biological applications.

##### 2.3.2.1 Titanium‐Based Nanocomposites

Titanium (Ti) is a light and white‐silvery transition‐metallic element with strong mechanical and corrosion‐resistant properties. Ti and its compounds have long been tracked in clinical applications, primarily as bone implants, and subsequently, their antimicrobial, immunomodulatory, and anticancer properties have been studied. In addition, titanium carbide MXene (Ti_3_C_2_T_x_), the first synthesized composition of MXene in 2017,^[^ ^181^ ^]^ led to the advent of the generation of a new class of 2D nano/biomaterials in the form of MXene nanosheets and quantum dots in bioimaging and biosensing.^[^ ^182^, ^183^, ^184^ ^]^ Since their advent in biomedical applications, MXenes have been fabricated using various etchants, intercalants and functionalized postsyntheses for enhanced bioactivity and improved physiochemical properties.^[^ ^185^, ^186^, ^187^ ^]^ Surface terminations create avenues for surface modifications and aid in the conjugation of drugs on the MXene surface. Ti_3_C_2_ MXene nanodots are used in bioimaging.^[^ ^188^, ^189^, ^190^ ^]^ A Ti_3_C_2_ MXene surface modified with soybean phospholipid (SP) was the first demonstration of the use of MXenes for photothermal therapy (PTT).^[^ ^191^ ^]^ Furthermore, Ti_3_C_2_‐doxorubicin (Ti_3_C_2_‐DOX) MXenes, which are surface modified with hyaluronic acid (HA), SP and Ti_3_C_2_‐MnO_x_ MXenes modified with SP, are used in PTT/photodynamic (PDT)/chemotherapy by acting as efficient photothermal agents (PAs) in cancer theranostics.^[^ ^192^, ^193^, ^194^ ^]^

The surface‐modified MXenes exhibited enhanced photothermal conversion efficiency, as the heat conversion efficiency determines their ability to kill tumor cells. PEG‐modified Ti_2_C MXenes efficiently cause minimal damage to healthy nonmalignant cells during PTT.^[^ ^195^ ^]^ Additionally, Ti_3_C_2_ loaded with titanium dioxide (TiO_2_) nanoparticles was found to be an efficient sonodynamic therapy (SDT) agent. Ti_3_C_2_ has PTT effects, and TiO_2_ aids in SDT; combining both as Ti_3_C_2_‐TiO_2‐x_ shows highly advantageous therapeutic effects against cancer as a multifunctional nanohybrid.^[^ ^196^ ^]^ SP‐modified Ti_3_C_2_ loaded with DOX and cellulose‐integrated Ti_3_C_2_ with DOX are used for the delivery of chemotherapeutic agents, thus playing a synergistic role in chemotherapy and PTT.^[^ ^197^, ^198^, ^199^ ^]^

**Figure 10:**
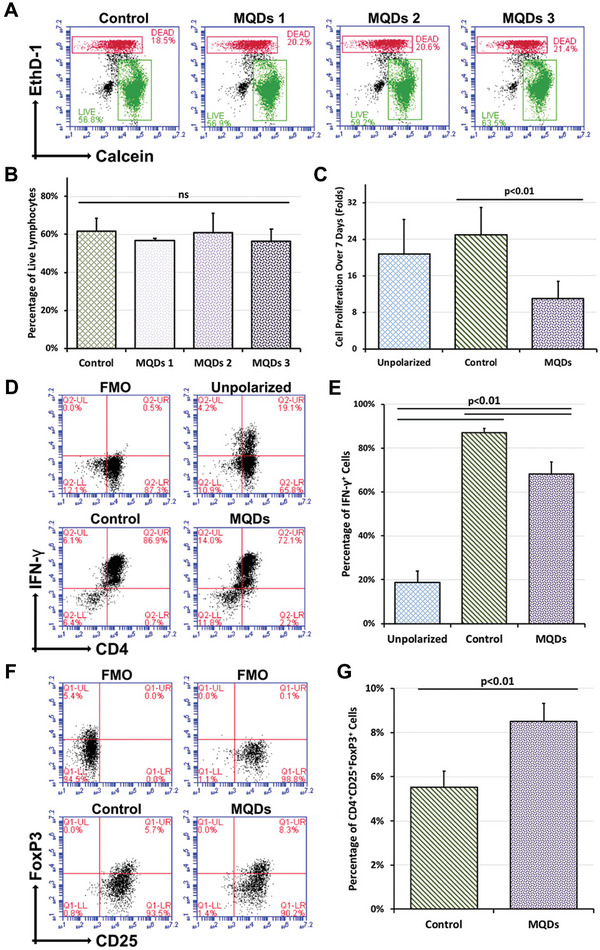
(A) Cytotoxicity assessment of titanium carbide MXene/oxide (Ti_3_C_2_T_x_ MQDs) with human peripheral blood mononuclear cells at different concentrations of 66 ng mL^−1^ (1x), 10x and 20x after 24 hours of culture via flow cytometry. The live cells were stained green with calcein AM, and the dead cells were stained red with ethidium homodimer‐1. (B) The percentage of viable live lymphocytes. (C) Quantification of stimulated naïve CD4^+^ T lymphocyte proliferation after 7 days of the experiment. (D) The immune modulatory effects of these MQDs on human T lymphocytes were evaluated. To do this, flow cytometry phenotyping was applied to stimulated naïve CD4^+^ T cells, and the cells were identified and quantified accordingly. (E) Compared with the control treatment, treatment with these MQDs effectively reduced the percentage of proinflammatory CD4^+^IFN‐γ^+^ cells. (F) Identification of CD4^+^ CD25^+^ FoxP3^+^ T‐regulatory cells. (G) Effects of these MQDs on the percentage of CD4^+^CD25^+^FoxP3^+^ regulatory T cells (Reproduced with permission.^[^ ^200^ ^]^ Copyright 2019, Wiley‐VCH GmbH).

Interestingly, Ti_3_C_2_‐based MXenes are among the first transition metal‐based biomaterials reported for their alluring immunomodulatory properties to manage graft rejection. This study was carried out in our laboratory (**Figure** **10**), and we demonstrated that MXenes are highly biocompatible with human peripheral blood mononuclear cells at concentrations of up to 1000 ng mL^−1^ after 24 hours of culture, with no significant cytotoxicity for up to seven days. In addition, the immunomodulatory assessments revealed a significant decrease in lymphocyte proliferation in the MXene groups compared with the control samples, indicating a decrease in lymphocyte activation in response to the observed proinflammatory stimuli. They further reduced the percentage of CD4^+^, IFN‐γ^+^, and T_h_‐1 inflammatory cells and increased the percentage of CD4^+^ CD25^+^ forkhead box P3 (FOXP3^+^) regulatory T cells. This concept was first established in our lab by the synthesis of Ti_3_C_2_ MXene‐quantum dots as next‐generation smart nanomaterials and administering them in vivo through a chitosan‐based MXene composite hydrogel for immunomodulation.^[^ ^200^ ^]^

**Figure 11:**
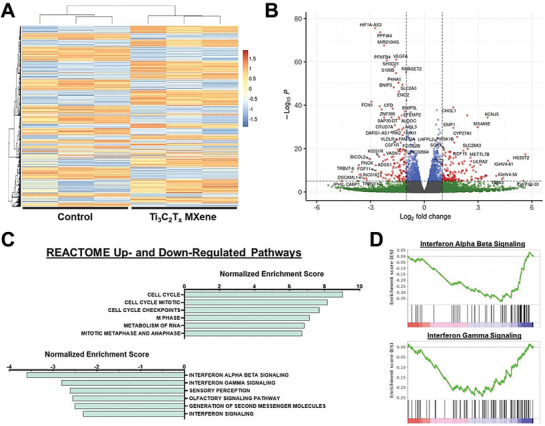
Transcriptomic analysis (RNA sequencing) of lymphocytes cultured with MXene nanosheet‐treated endothelial cells in a coculture system. Human peripheral blood mononuclear cells (HPBMs) were cultured with Ti_3_C_2_T_x_ MXene‐treated HUVECs followed by activation with interferon (INF)‐gamma. (A, B) A total of ∼37,000 genes were detected within the treated lymphocytes. Among them, ∼2300 genes (6.3 per cent) were significantly upregulated, and approximately 2100 genes were downregulated in the cells cultured with HUVECs treated with MXene at a concentration of 2 µg^/mL^. (C) Gene set enrichment analysis against 1026 REACTOMEs (the top upregulated pathways are normalized on the basis of cell cycle regulation, and the top downregulated pathways are related to IFN signaling). (D) The graphs show that both IFN‐alpha beta and IFN‐gamma have remarkable enrichment of downregulated genes in the tested samples (n = 3 per group) (Reproduced with permission.^[^ ^201^ ^]^ Copyright 2023, Elsevier Ltd.).

Our laboratory recently reported the first immunomodulatory application of Ti_3_C_2_T_x_ MXenes to treat allograft vasculopathy.^[^ ^201^ ^]^ The highly biocompatible and bioactive nature of Ti_3_C_2_T_x_ MXenes allows them to interact with human endothelial cells (ECs), regulating alloantigen presentation and cellular adhesion. Molecular evaluation revealed reduced endothelial expression of key antigen presentation mediators (HLA‐1, IRF1, TAP1, and B2M), significant downregulation of PECAM1 and VE‐CDH5, and upregulation of leukocyte adhesion molecules. These findings revealed the effects of MXenes on cell adhesion and maintenance of the normal endothelial phenotype. RNA sequencing analysis (see **Figure** **11**) and quantitative PCR revealed _that Ti3C2Tx_ MXenes mitigate immune cell infiltration through the top upregulated pathways related to cell cycle regulation. A reduction in the endothelial immune activation of T lymphocytes (decreased CD4 and CD28 expression both in vitro and in vivo) and amelioration of the pathogenesis of allograft vasculopathy by the significant downregulation of gene sets related to interferon alpha beta signaling and interferon gamma signaling were the other findings of our research to support the immunoengineered MXene nanosystem‐mediated treatment of transplant allograft vasculopathy.

##### 2.3.2.2 Zirconium‐based Nanocomposites

The physical and chemical properties of zirconium (Zr), a heat‐ and corrosion‐resistant transition metal, are similar to those of titanium. 2D zirconium has a role in photocatalysis, sensing, conductivity, lubricant and optoelectronics‐based applications.^[^ ^202^ ^]^ Ultrathin zirconium‐based 2D metal‒organic frameworks (Zr‒MOFs) are more advantageous than 3D bulk MOFs because of their enhanced catalytic activity, large surface area, and thermal properties, indicating great potential in photocatalysis.^[^ ^203^ ^]^ Owing to their high structural tunability and biodegradability, MOFs are multifunctional and useful in biological applications. Zr‐MOFs have greater accuracy in early cancer cell detection and biosensing, particularly in the detection of the early marker protein mucin 1 (MUC1), where the overexpression of MUC1 is characteristic of different malignancies.^[^ ^204^ ^]^ MUC1 plays an intrinsic role in regulating and modulating infection‐induced and pathogen‐induced inflammatory responses, respectively, suggesting that Zr‐MOF detection of MUC1 is a clinical marker for chronic respiratory‐related inflammatory diseases. Thus, zirconium‐based nanosheet materials are promising for potential immune‐biosensor applications.

##### 2.3.2.3 Hafnium‐based Nanocomposites

Hafnium, a tetravalent transition metal that chemically resembles zirconium, has diverse applications in industrial settings, such as semiconductors, nuclear reactors and alloys, in combination with niobium, titanium, and tungsten. Hafnium oxide (HfO_2_) is the first biosensor used to assess cortisol levels in heart failure patients via electrochemical impedance spectroscopy (EIS). HfO_2_ acted as the substrate bearing the functionalized magnetic nanoparticles and antibodies as self‐assembled monolayers (SAMs) and aided in the detection of cortisol via its interaction with the polyclonal antibody via EIS analysis in cardiac disease management.^[^ ^205^ ^]^

Hafnium‐based two‐dimensional metal organic layers (2D‐MOLs) and three‐dimensional metal‐organic frameworks (3D‐MOFs) connected by hafnium clusters are utilized in fluorescence sensing,^[^ ^206^ ^]^ biomimetic sensing, catalysis and biorelated analytical separation applications.^[^ ^207^ ^]^ Due to their multifunctional use in biological systems, transition metal‐based nanoparticles, including hafnium‐based nanoparticles (Hf‐NPs), have gained importance in cancer therapies. Understanding the mechanism of Hf‐NPs in the biophysiological microenvironment is critical for their effective application beyond traditional industrial uses.^[^ ^208^ ^]^ As the particle size decreases, the reactive surface area increases, ultimately increasing the toxicity of the nanoparticles to cancer cells. Thus, careful evaluation of the potential toxicity of Hf‐NPs in normal and cancerous human cells is needed. Additionally, the reduction in the size of the NPs allows easy cellular uptake, systemic distribution throughout the body and potential deflection of macrophage defenses. Testing the cytotoxic nature of HfO_2_ and HfO_2_‐NPs in different human cell lines and other microbial systems revealed that these particles do not cause acute or severe toxicity to cells.^[^ ^209^ ^]^

**Figure 12:**
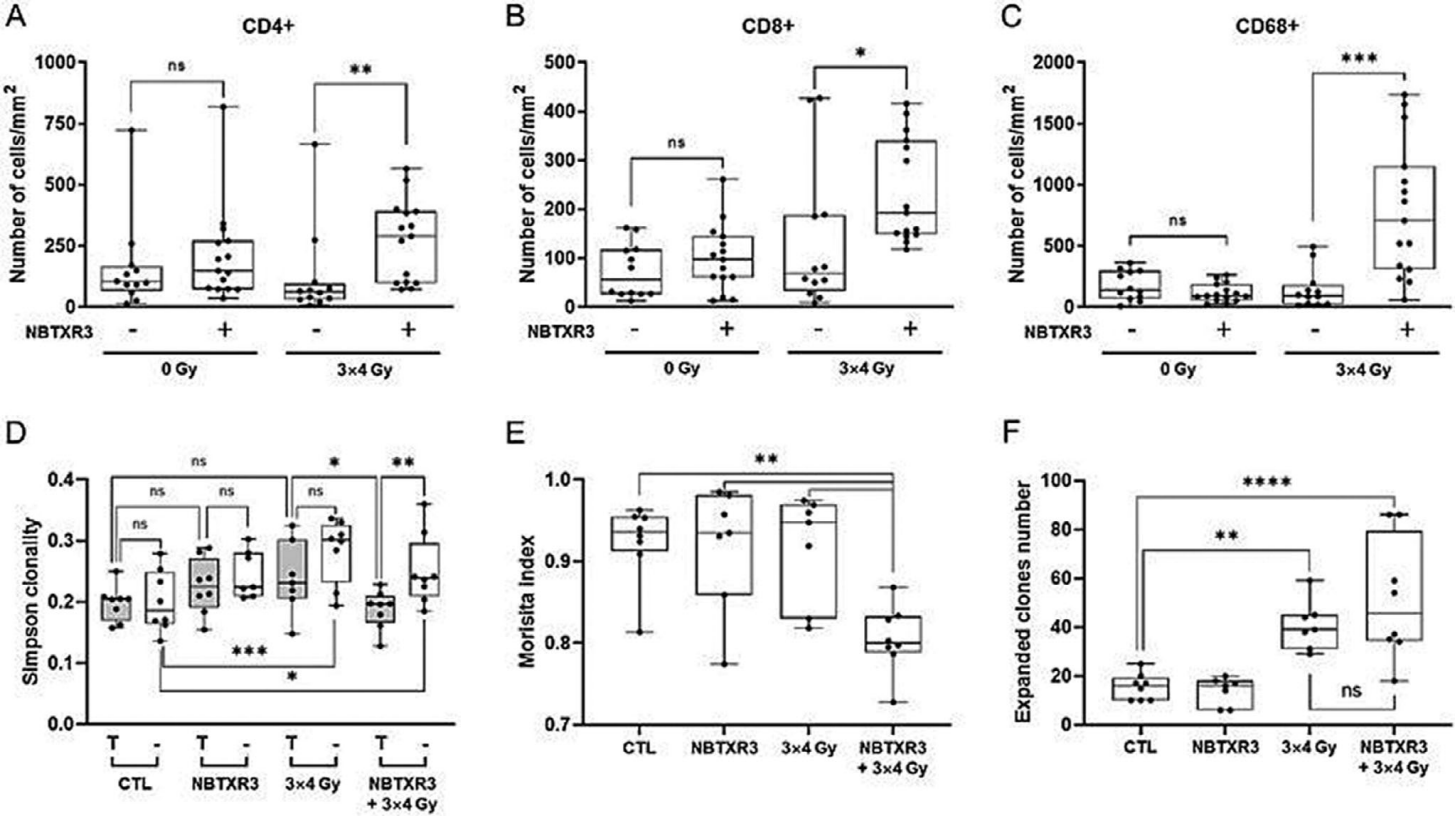
Modulation of T‐cell receptor (TCR) and immune cell infiltration by a novel radioenhancer composed of functionalized hafnium oxide crystalline nanoparticles (NBTXR3) activated by radiation therapy (RT). Cell density measurements of (A) CD4^+^, (B) CD8^+^, and (C) CD68^+^ infiltrates in the treated tumors analyzed by immunohistochemistry (IHC) at 5 days after the last RT fraction. For each sample, 3 slices of tumor formalin‐fixed, paraffin‐embedded (FFPE) blocks (the first, middle and third sections of each tumor sample) were stained with antibodies (n = 4–5 mice per group). TCR repertoire analysis, box/whisker representation plots of (D) Simpson‐clonality, (E) Morisita index, and (F) expanded clones of treated (T) and nontreated (–) CT26 tumors. WT‐bearing model mice 3 days after the last RT fraction (n = 7–8 mice per group were analyzed by one‐way ANOVA for Simpson‐clonality and expanded‐clones. The Mann‒Whitney test was used for the other variables, including statistical significance, marked as *p < 0.05, **p *<* 0.01, ***p *<* 0.001, and ****p *<* 0.000. (Reproduced with permission.^[^ ^217^ ^]^ Copyright 2022, Springer Nature, Author(s)).

Hf‐NPs especially show promise in radiation‐based anticancer approaches because of their ability to enhance radiation dose effectiveness. The high number _of HfO2_‐NPs is considered favorable for radiation‐based cancer therapy because it enables adequate local and systemic tolerance, good interaction with high ionizing radiation and the deposition of high energy in the tumor region. The systemic tolerance of HfO2‐NPs is attributed to their nontoxic nature when taken up by phagocytic cells, making them nonimmunogenic, chemically inert, and have minimal health hazards, making them viable for enlarging the therapeutic window of radiation‐based cancer therapies.^[^ ^210^ ^]^ Hafnium ions are high‐Z metal ions with high molar extinction and photoelectric effects, making them effective as metal‒polyphenolic nanosystems for photothermal therapy (PTT) and radiation therapy (RT). The polyphenolic system led to photothermal conversion (PTC) in the NIR‐II biowindows, and chelated hafnium radiosensitization led to the neutralization of tumor reoccurrence, which is prevalent in PTT.^[^ ^211^ ^]^ Thus, this synergistic hafnium‐sensitized RT and PTT can be effectively used in cancer theranostics. Under X‐ray irradiation, hafnium sensitizes abnormal ROS generation caused by radio‐dynamic therapy (RDT) as a nanoprocessor composed of hemoglobin (Hb), hafnium, and chlorin e6 (Ce6). The combined effects of Hb‐Hf‐Ce6 led to the modulation of the oxygen balance in the tumor microenvironment and the induction of an antitumor immune response.^[^ ^212^ ^]^ Subsequently, the radioenhancer NBTXR3 with functionalized HfO_2_‐NPs is used for radiotherapy (RT) to treat liver cancer, which is currently in clinical trials.^[^ ^213^, ^214^ ^]^ Radiotherapy activates the NBTXR3 HfO_2_‐NPs that target tumor cells, and the NPs are chemically inert in cellular and subcellular systems, resulting in favorable systemic tolerance. These findings support its use in clinical trials to treat cancer with radiation therapy via on/off modes of action.^[^ ^215^ ^]^ HfO_2_‐NPs serve as nanoenhancers, combining radiosensitization and tumor cell death by radiation in various cancer theranostic approaches at the cellular and molecular levels.^[^ ^216^ ^]^ To overcome the immunogenicity caused by radiotherapy and address the antitumor immune response stimulated by RT, RT‐activated NBTXR3 (functionalized HfO_2_‐NPs) were tailored to act as effective immunomodulators by affecting the lymphocyte population and altering tumor cell immunogenicity through immunopeptidome modulation (**Figure** **12**).^[^ ^217^ ^]^ RT‐activated NBTXR3 results in a specific adaptive immune response by significantly increasing CD3^+^ and CD8^+^ T‐cell populations with enriched cytokine, adaptive immunity and T‐cell receptor signaling pathway genes in patients with soft tissue sarcoma (STS).^[^ ^218^ ^]^ These compounds are used to treat locally advanced STS in phase 2–3 controlled clinical trials, where the effect of RT‐activated NBTXR3 has been studied in comparison with that of conventional RT.^[^ ^219^ ^]^ These findings emphasize the role of the RT‐activated high‐Z hafnium radioenhancer as an effective modulator of cancer immunogenicity in anticancer radiation‐mediated immune therapies. Recently, it has been reported that nanoscale metal‒organic frameworks (nMOFs) offer better radiosensitizing ability than solid NPs do. A comparison of the efficacy of NBTXR3 with that of Hf‐oxo nMOF revealed that the dose‐enhanced radiosensitizing effect of the nMOFs was efficient in controlling the ROS and DNA damage caused by radiation therapy in cancer treatment.^[^ ^100^ ^]^

**Figure 13:**
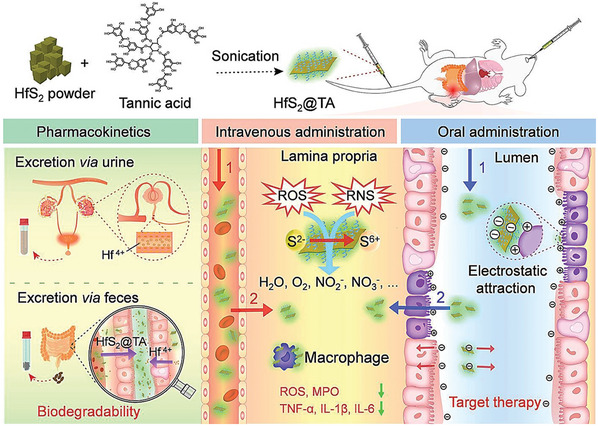
Schematic depicting the exfoliation of degradable hafnium disulfide nanosheet (HfS_2_)‐modified tannic acid (TA) with potential bioactivity properties for the treatment of inflammatory bowel disease via oral administration or intravenous processes (Reproduced with permission.^[^ ^220^ ^]^ Copyright 2022, American Chemical Society Publications).

Hf‐NPs are employed beyond tumor‐related applications because of their role as anti‐inflammatory nanoagents to treat inflammatory diseases. Tannic acid‐coated hafnium disulfide nanosheets (HfS_2_‐TA‐NSs) were developed for targeted therapy of inflammatory bowel disease (IBD). HfS_2_‐TA‐NSs were found to eliminate excessive ROS and reactive nitrogen species (RNS) in an IBD model due to their high specific area and number of reactive sites. The excellent targeting ability of HfS_2_‐TA‐NSs (**Figure** **13**) and their ability to accumulate in the intestinal mucosa via electrostatic interactions led to the inhibition of inflammation and aided in repairing the intestinal mucosa by downregulating proinflammatory cytokines, which were subsequently excreted by the kidney and hepato‐intestinal systems. This finding highlights the potential of HfS_2_‐TA‐NSs to have immunomodulatory properties as anti‐inflammatory two‐dimensional nanoagents combined with hafnium in CT imaging for IBD‐targeted diagnosis and therapy in the future.^[^ ^220^ ^]^

Carbon dots (CDs) are promising nanoprobes for bioimaging techniques such as noninvasive tumor imaging and diagnosis. Hafnium‐based carbon dots (Hf‐CDs) are biocompatible, enhancing tumor site accumulation and providing excellent renal clearance, overcoming the limitations of conventional carbon dots, with significant computed tomography (CT) performance in an orthotropic liver cancer model.^[^ ^221^ ^]^ Thus, hafnium‐doped CD materials have potential for use as multimodal nanoprobes in bioimaging and in clinical diagnosis. HfO_2_ layers also aid in bone tissue engineering applications by exerting anti‐inflammatory effects, enhancing osteogenesis, and reducing osteoclastogenesis in an osteoporotic bone fracture model without eliciting an immune response. These findings indicate that the HfO_2_ biomaterial can be used to restore homeostasis by modulating the imbalance between bone formation and bone resorption and, consequently, enhancing the healing process in patients with bone‐related disorders.^[^ ^222^ ^]^

The above studies show the dynamic and versatile applications of nanohafnium‐based materials in biosensing, radiation therapy, photothermal therapy, radiation‐mediated immunotherapy, immunomodulation and tissue engineering.

#### 2.3.3 Group‐5 Elements and Reported Immunoengineering Applications

Group 5 of the period table of chemical elements consists of vanadium (V), niobium (Nb), tantalum (Ta), and dubnium (Db) transition metals. Dubnium is a radioactive transuranium element described as a crucial component of nanoparticles that can be intravenously injected into the body and can be irradiated by a laser.^[^ ^223^ ^]^ Subsequently, dubnium nanoparticles have been developed and studied for their ability to absorb energy from bean radiation and convert it into heat in a short span of time to target cancer cells and extend their application in anticancer therapy.^[^ ^224^ ^]^ The following sections discuss the well‐researched immunomodulatory applications of 2D Va, Nb and Ta.

##### 2.3.3.1 Vanadium‐based Nanocomposites

Vanadium is a rare, ductile, gray‒white metallic element found in different animals, and natural resources are widely used in industry because of its optical and magnetic properties, which are attributed to its low neutron absorbing properties. It can be used to treat type 2 diabetes by lowering blood sugar levels.^[^ ^225^ ^]^ Vanadium is easily cleared by the body, which limits its bioavailability, and high‐dose administration of vanadium causes toxicity issues. Therefore, vanadium‐based nanomaterials have been developed to overcome the drawbacks of vanadium in its native state. These nanomaterials have been used in various cancer therapies and in tumor diagnosis.^[^ ^226^ ^]^ Vanadodrugs are vanadium‐based therapeutics for cancer with highly modifiable chemical and structural properties that target malignant tissues, inhibit tumor growth and cancer cell proliferation, and limit the invasion and metastasis of neoplastic cells. They influence miRNA expression, stem cell differentiation and autophagy, making them effective anticancer agents with specificity, selectivity and effectiveness for future metallodrug applications.^[^ ^227^ ^]^

Vanadium oxide nanoparticles (V_2_O_3_‐NPs), as transition metal oxides (TMOs), create strong oxidative stress, which impairs cellular functions in a biological system because of their high surface reactivity. Owing to this, V_2_O_3_‐NPs are potentially toxic to human endo‐ and epithelial lung cells and can be exploited to target malignant lung tissues.^[^ ^228^ ^]^ Vanadium pentoxide (V_2_O_5_) is the most stable thermodynamic form of vanadium‐oxygen compounds,^[^ ^229^ ^]^ and its antitumor effects have been validated both in vitro and in vivo. Vanadium pentoxide nanoparticles (V_2_O_5_‐NPs) present pro‐ and antioxidative properties, altering the mRNA levels of antioxidant genes and influencing autophagy and apoptosis. Hyperaccumulation of V_2_O_5_‐NPs induces autophagy‐ and mitophagy‐mediated mitochondrial damage, leading to lysosomal dysfunction and influencing cell cycle arrest and apoptosis. V_2_O_5_‐NPs are selective for the treatment of breast cancer cells, making them promising candidates for the treatment of chemoresistant cancer.^[^ ^230^ ^]^

As a group of transition metal elements, vanadium carbide MXenes are of interest for a wide range of energy storage applications. Vanadium carbide MXene (V_2_CT_x_) nanosheets synthesized from V_2_AlC,^[^ ^231^, ^232^ ^]^ are of interest for a wide range of energy storage applications and biomedical applications, particularly in photothermal therapy (PTT) such as its counterpart Ti_3_C_2_T_x_. The biocompatibility of V_2_CT_x_ is questionable in biological systems because of its rapid oxidation^[^ ^233^ ^]^ and decomposition into vanadium oxide mixtures,^[^ ^234^ ^]^ which cause cytotoxicity, cell cycle disruption and cell membrane damage.^[^ ^235^ ^]^ Conventional methods of V_2_CT_x_ synthesis result in low photothermal conversion efficiency (PTCE); hence, a green delamination method in which algae intercalate and delaminate the V_2_AlC MAX phase results in V_2_CT_x_ MXene nanosheets with a PTCE as high as 48%.^[^ ^236^ ^]^ Another interesting feature of V_2_CT_x_ is that it exhibits the enzyme‐mimetic properties of superoxide dismutase (SOD), peroxidase (POD), catalase (CAT) and glutathione peroxidase (GPx) and acts as a theranostic nanozyme to suppress ROS elevation after ischemic stroke. V_2_CT_x_ scavenges ROS and blocks ROS‐induced inflammation, extending its antioxidative property against oxidative injury and leading to neuronal apoptosis. Thus, V_2_CT_x_ can act as a regulator of inflammation and be used to treat ROS‐related brain diseases.^[^ ^237^ ^]^ Inspired by their cytotoxic nature and PTT effect, vanadium carbide MXene‐derived quantum dots (V_2_CT_x_‐QDs) were synthesized as sonodynamic therapeutic agents to hinder antioxidant cellular mechanisms to aid in ROS generation when triggered by ultrasound irradiation to be potent in ROS‐mediated sonodynamic anticancer therapy.^[^ ^238^ ^]^

The combination of antioxidant, photothermal‐based cancer therapy and the immunoregulatory properties of vanadium‐based nanoforms makes engineering cytotoxic 2D vanadium crucial in the development of future immune‐cancer therapeutics.

##### 2.3.3.2 Niobium‐based Nanocomposites

Niobium is a ductile transition metal commonly used to coat metals, alloys, and implants that are implanted in the human body at the site of injury, as it is highly resistant to physiological corrosion and is biocompatible. Niobium carbide MXenes (Nb_2_CT_x_) were the first reported photothermal agents (PTA) for PTT in 2017 but had a low PTCE of 28.6% in the NIR‐II region.^[^ ^239^ ^]^ To enhance its PTCE, surface‐engineered Nb_2_CT_x_ materials have been developed. Surface_‐modified Nb2CTx_ with polyvinylpyrrolidone (PVP) increased the PTCE to 36.4% and 45.65% in the NIR‐I and NIR‐II biowindows, respectively, suggesting that this material is an efficient antitumor therapeutic agent.^[^ ^34^ ^]^ Polydopamine (PDA)‐modified Nb_2_CT_x_ combined with an immunoadjuvant was developed to confront the tumor‐associated antigens (TAAs) that are released during photothermal ablation of tumor cells. These TAAs induce immunogenic cell death, and the addition of an immunoadjuvant to Nb_2_CT_x_ enhances their immunotherapeutic activity. In addition to the photothermal therapeutic activity elicited by Nb_2_CT_x_, PDA modification helps increase the loading of the immunoadjuvant, making this MXene system synergistic in PTT and immunotherapy.^[^ ^240^ ^]^

**Figure 14:**
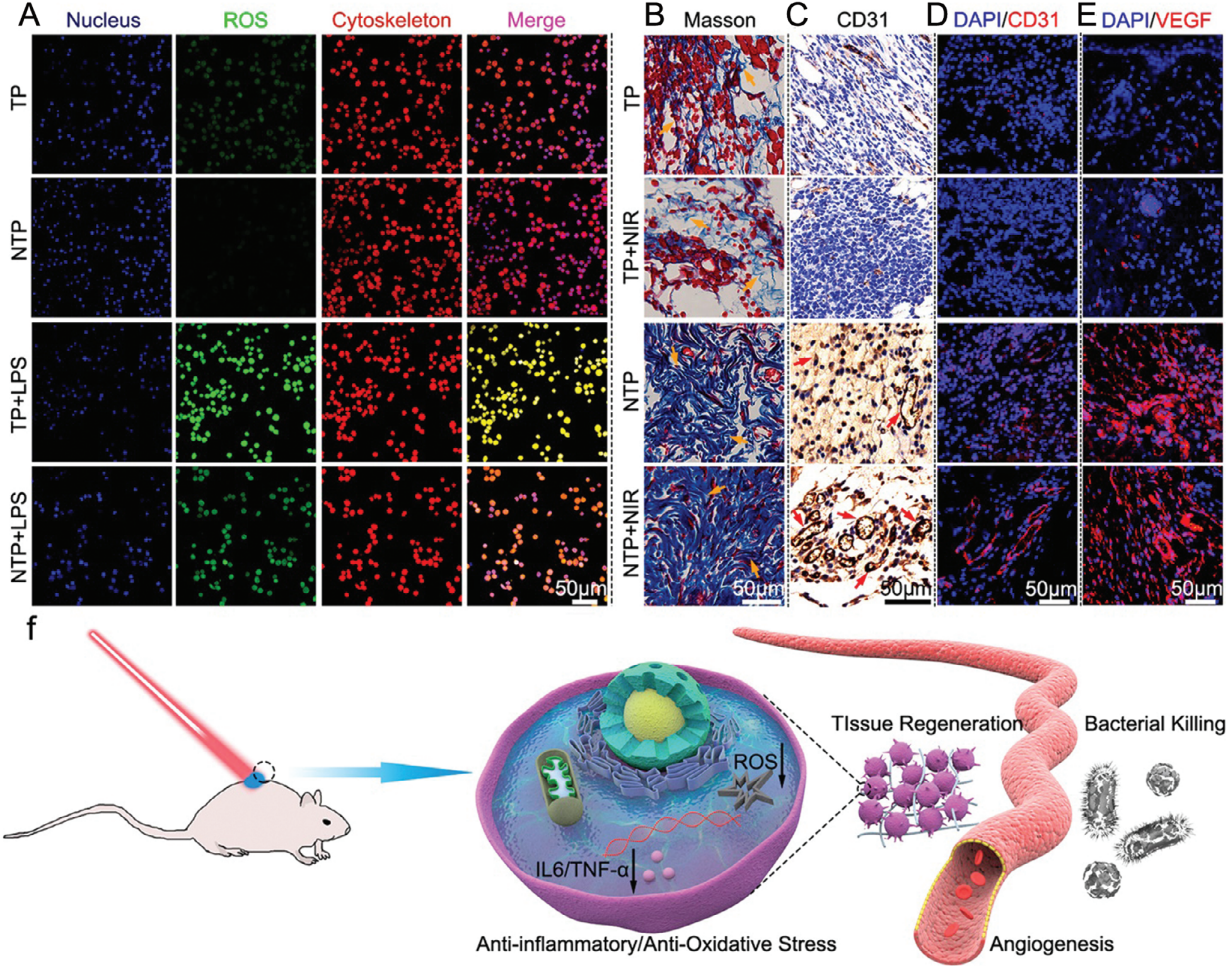
(A) Fluorescence microscopy images of intracellular ROS. (B) Masson's trichrome staining revealed collagen fibers. (C) Immunohistochemical staining of the cells. (D, E) Demonstration of immune fluorescence images of CD31 and VEGF. (F) Schematic of the in vivo multimodal infection management of designed material‐based thermotherapy. These data suggest the potential of the synthesized system for ROS scavenging and accelerating wound healing (Reproduced with permission.^[^ ^242^ ^]^ Copyright 2020, American Chemical Society).

Moreover, multifunctional Nb_2_CT_x_ was developed by coating nanosheets with silica (Si) and an immune adjuvant and then 3D printed on biodegradable bioglass (BG) scaffolds as immune‐engineered nanomaterials for tissue regeneration. This composite has both PTT effects on target cancer cells and tissue regeneration potential due to the presence of Nb and Si, which aid in bone regeneration. The immune adjuvant helped the composite activate the immune response for anticancer therapies. This composite MXene nanosystem acts as a combined therapy to target tumor cells and the immune response, thereby promoting tissue regeneration in bone metastasis caused by breast cancer.^[^ ^241^ ^]^ With the history of the use of niobium as an alloy to coat metal implants, Nb_2_CT_x_‐coated titanium plate implants with anti‐infection properties have been developed for clinical use. By utilizing the photothermal performance of Nb_2_CT_x_ as a thermoablation agent to increase bacterial infection resistance when it is implanted, these clinical implants were found to impact the immune response by increasing proinflammatory responses and ROS scavenging, aiding in angiogenesis and tissue remodeling.^[^ ^242^ ^]^ As shown in **Figure** **14**, their data suggest effective alleviation of oxidative stress in material‐treated immune cells and macrophages compared with or without lipopolysaccharide stimulation. In addition, ultrathin Nb_2_C nanosheets were developed to target inflammatory bowel disease by targeting ROS, reducing intracellular ROS levels and promoting cell survival.^[^ ^243^ ^]^ This evidence demonstrates the potential of 2D niobium as an effective PTA and antioxidant‐based immunoregulator for treating ROS‐related diseases, cancer therapy, tissue regeneration and immune modulation in medical applications.

##### 2.3.3.3 Tantalum‐based Nanocomposites

**Figure 15:**
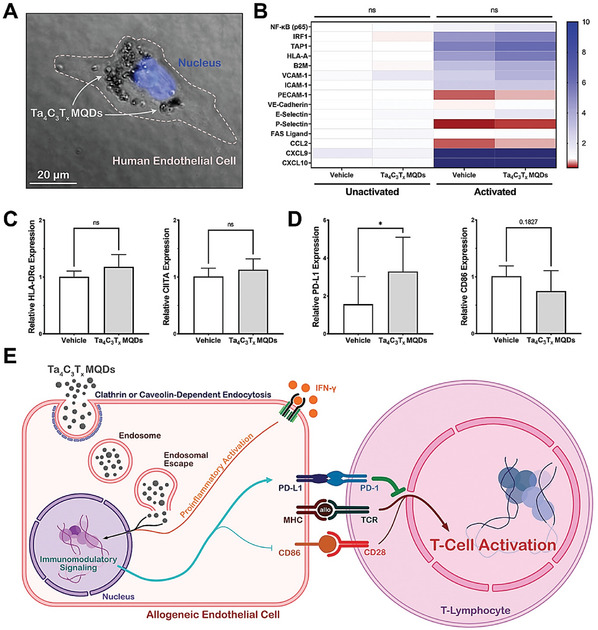
Mechanistic immunomodulatory evaluation of Ta_4_C_3_T*_x_* MQDs. (A) Light microscopy revealed that these MQDs were internalized into cultured human vein endothelial cells (HUVECs) after 24 hours. (B, C) Quantitative PCR analysis of genes involved in antigen presentation, lymphocyte recruitment, cellular adhesion, and chemokine signaling. (D) Treatment with these MQDs altered the expression of the T‐cell coinhibitor on the surface of these activated cells. (E) Schematic of their immunomodulatory mechanisms in reducing T‐cell activation (Reproduced with permission.^[^ ^247^ ^]^ Copyright 2021, Wiley‐VCH GmbH).

**Figure 16:**
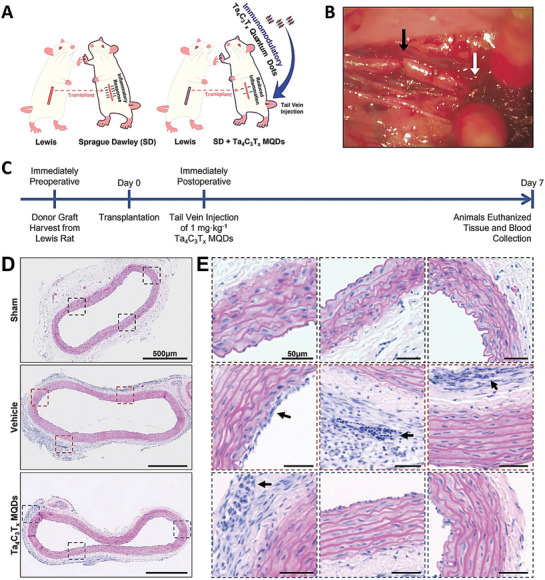
(A) Schematic illustration and (B‐E) in vivo immunomodulatory validation of the immunoengineered Ta_4_C_3_T*_x_* MQD‐based system in a tested rat cardiac allograft vasculopathy model. The animals received a tail‐vein injection at a concentration of 1 mg kg^−1^/body weight for seven days. (B) A representative photograph of a transplanted aortic segment. (C) Experimental timeline. (D, E) H&E staining confirmed the explanted abdominal segments. Inflammation signs are obvious in the transplanted animals. Furthermore, there appeared to be significant reductions in cell thickening and lymphocyte infiltration in the MQD‐treated group compared with the control group (Reproduced with permission.^[^ ^247^ ^]^ Copyright 2021, Wiley‐VCH GmbH).

Tantalum is a soft, silvery, lustrous transition metal with desirable physiochemical properties for a wide range of applications. Owing to its high corrosion resistance, it is utilized in the synthesis of electronic components, as a thin layer coating of metals to achieve high capacitance, and in the coating of metal implants because of its high corrosion resistance, which is contributed by its oxide form in aqueous media. Tantalum is less toxic than titanium and can be effectively used in biological applications such as tantalum carbide MXenes (Ta_4_C_3_T_x_), such as Ti_3_C_2_T_x_. Surface‐modified Ta4C3Tx has been applied in tumor ablation, especially Ta4C3Tx functionalized with superparamagnetic iron oxide nanoparticles (Ta_4_C_3_T_x_‐IONPs), and furthermore, surfaces modified with soybean phospholipids (SPs) are efficient photothermal agents (PTAs) in breast cancer theranostics. The PTCE of these Ta_4_C_3_T_x_‐IONP‐SP nanosheets was 32.5%, which effectively eradicated tumors without reoccurrence. The IONP‐SP conjugation aided in magnetic resonance imaging, thus making the Ta_4_C_3_T_x_‐IONP‐SP composite an efficient bioimaging tool for diagnosis and PTT.^[^ ^244^ ^]^ Similarly, manganese oxide nanoparticle‐functionalized Ta_4_C_3_T_x_ (Ta_4_C_3_T_x_‐MnO_x_) is a promising candidate for cancer theranostics, with an enhanced PTCE of 34.9% and enhanced photoacoustic imaging (PAI) potential due to the MnO_x_ component.^[^ ^245^ ^]^ Ta_4_C_3_T_x_ is gaining attention in bioelectronics because of its exceptional electronic, optical, and magnetic properties. Accordingly, we developed a highly biocompatible Ta_4_C_3_T_x_‐tantalum oxide (TTO) nanohybrid by adapting an oxidized fluorine‐free method of synthesis. The oxygen‐containing functional groups contributed to the electrochemical performance of the material, which is crucial for bioelectronic applications.^[^ ^246^ ^]^ Owing to the enhanced biocompatibility of Ta_4_C_3_T_x_, we further investigated these nanomaterials in the form of quantum dots (Ta_4_C_3_T*_x_* MQDs) for immunomodulatory applications for the first time. The immunoengineered Ta_4_C_3_T*_x_* MQDs efficiently modulate the immune response during allograft vasculopathy by reducing immune cell infiltration during organ transplantation. Our study revealed the promising application of these MQDs in immunomodulation‐related applications,^[^ ^247^ ^]^ as they caused a remarkable shift in the expression pattern of surface coinhibitory and costimulatory molecules in HUVECs and significantly increased the expression of programmed death ligand‐1 in activated HUVECs compared with that in control cells (∼3.3‐fold). In addition, (**Figure** **15**), there was a marked difference in the expression levels of PD‐L1 and CD86 in activated HUVECs treated with MQDs (1.3‐fold, *p* = 0.182), suggesting their role in T‐cell‐activation pathways as coinhibitors and coactivators, respectively, via cultured antigen‐presenting cells. We also observed that there was no significant change in the percentage of Th2 cells, confirming that immunomodulation was achieved by the modulation of Th1 cells. In vivo experiments revealed that the immunoengineered MQDs exhibited effective immunomodulatory properties without significant cytotoxic effects on cells, tissues, or organs in the tested aortic transplantation rodent models (see **Figure** **16**).

These findings highlight the potential of Ta_4_C_3_T*_x_* in combining cancer therapy and tissue regeneration, similar to its predecessors V and Nb, with promising immunomodulatory applications in preventing organ transplant failure.

#### 2.3.4 Group‐6 Elements and Reported Immunoengineering Applications

The Group 6 elements of the periodic table include the following transition metals: chromium (Cr), molybdenum (Mo), tungsten (W), and seaborgium (Sg). Cr, Mo and W are refractory metals with reported biomedical and immunomodulatory applications, which are discussed below. The radioactivity and rarity of Sg limits its biological use due to potential toxicity; hence, there are no reports related to the immunomodulatory properties of 2D seaborgium nanosheets.

##### 2.3.4.1 Chromium‐Based Nanocomposites

Chromium is widely used in metallurgy and enhances insulin activity in individuals with type 2 diabetes, preventing the risk of cardiovascular diseases.^[^ ^248^ ^]^ Nanoforms of chromium conjugated with supplements, such as nanochromium‐containing metal‒organic frameworks (MOFs), are being explored to improve insulin resistance and repair impaired glucose metabolism by extending their antioxidant and anti‐inflammatory effects to ameliorate it.^[^ ^249^ ^]^ Another study showed that nano chromium picolinate (nCrPic) affected insulin‐related signaling pathways in skeletal muscle and adipose tissue in an in vivo pig model. nCrPic increased the expression of genes related to insulin resistance (PI3K, AKT, and adiponectin) and regulated genes related to glucose metabolism (SOCS3, UCP3, and IL‐15).^[^ ^250^ ^]^ Additionally, Cr‐based nanoparticles and microparticles of chromium, when alloyed with cobalt, have been tested for their cytotoxicity to fibroblasts to study particle size effects. Particle size is crucial in orthopedic implants, as it influences bioactivity and potential inflammation upon implantation.^[^ ^251^ ^]^ More recently, chromium nanoparticles (CrNPs) were found to address challenges associated with immune checkpoint blockade, a promising cancer immunotherapy.^[^ ^252^ ^]^ CrNPs enhance the infiltration of immune cells, particularly M1 macrophages and CD8^+^ T cells, via the MIP‐1α and PI3K/Akt/mTOR pathways. CrNPs also exhibit strong photothermal properties, and when combined with PD‐1/PD‐L1 inhibitors, they improve antitumor efficacy by alleviating the immunosuppressive TME and increasing immune cell infiltration.

Biocompatible ferromagnetic 2D materials can selectively target tumor cells without affecting healthy tissue by combining PTT via hyperthermia with magnetically directed drug delivery systems. Commonly explored manganese‐containing monolayers for ferromagnetic chemotherapy are neurotoxic; thus, chromium‐based monolayers have been developed and studied. Biocompatible CrX_3_ (X = chloride, bromide, iodide) monolayers exhibit ferromagnetic properties at room temperature and show great potential in cancer theranostics for magnetic biolabeling, drug delivery and hyperthermia treatment of cancer.^[^ ^253^ ^]^ Chromium‐based MXenes (Cr_2_CT_x_) are also ferromagnetic and are employed in spintronic applications^[^ ^254^ ^]^ and cancer theranostics. The functionalization of Cr_2_CT_x_ with multiple functional groups (Cr_2_CF(OH)) affects its optical properties and promotes its use as a photocatalyst for the hydrogen evolution reaction (HER).^[^ ^255^ ^]^ HER catalysts play a vital role in cancer therapy, and ongoing research on these Cr‐based catalysts has focused on optimizing their toxicity to tumor cells while minimizing side effects on healthy tissues.

These findings highlight the potential immunomodulatory effects of nano‐Cr, where its antioxidant, ferromagnetic, optical, photothermal and photocatalytic properties enable the selective targeting of tumor cells. This releases tumor antigens and increases immune cell infiltration by stimulating the immune system via immunogenic tumor cell death.

##### 2.3.4.2 Molybdenum‐Based Nanocomposites

Molybdenum is a trace element found in various dietary sources with diverse applications, including alloys, catalysts, lubricants, pigments, fertilizers and medicinal supplements. Molybdenum from dietary sources such as legumes, grains, dairy and meat helps in the body's metabolism by preventing molybdenum cofactor deficiency, a rare genetic neurological disorder. Molybdenum from mines and other industrial sources is harmful. Molybdenum‐based 2D materials can be synthesized via both top‐down and bottom‐up methods. Conventionally, Mo_2_C crystals were synthesized via a bottom‐up approach, chemical vapor deposition (CVD), but plasma‐enhanced pulsed laser deposition (PELPD) and template methods afforded nontoxic, biocompatible Mo_2_C and MoN nanosheets.^[^ ^199^ ^]^

Molybdenum carbide MXenes (Mo_2_CT_x_) derived from their metal carbide MAX phase Mo_2_Ga_2_C follow a top‐down approach, and like other MXenes, they are extensively used in energy storage applications. Unlike other MXenes, where the “A” of MAX has a single layer of “A”, the molybdenum MAX phase has two layers separating the Mo_2_C layers.^[^ ^256^ ^]^ Compared with other MXenes, Mo_2_CT_x_ is stable and has enhanced HER activity,^[^ ^257^ ^]^ making it a promising candidate for cancer theranostics. Mo_2_CT_x_ has been studied for its anticancer effects and ability to circulate in the reticuloendothelial system, showing a satisfactory biocompatible and biodegradable nature in vivo. Mo_2_CT_x_ surfaces modified with polyvinyl alcohol (PVA) serve as efficient PTAs, enabling effective photonic tumor ablation by inducing the release of lysosomotropic compounds into the cytoplasm during early cancer cell apoptosis. Mo_2_CT_x_‐PVA has a high PTCE of 24.5% in the NIR‐I and 43.3% in the NIR‐II biowindows, allowing deep tissue penetration and large permissible exposure of hyperthermia in tumors, making these MXenes excellent biodegradable PTAs for PTT.^[^ ^258^ ^]^

Furthermore, 2D molybdenum disulfide (MoS_2_) TMDs have versatile biological applications, including photothermal therapy, cancer theranostics, gene/drug delivery, bioimaging, biosensing and, recently, tissue engineering, as they aid in the differentiation of different stem cell lineages.^[^ ^259^, ^260^, ^261^ ^]^ These MoS_2_ nanosheets (MoS_2_‐NSs) are highly biocompatible at concentrations of up to ∼100 ppm and, compared with pristine nanosheets, are 10% less toxic to cells.^[^ ^262^ ^]^ Unlike other TMDs, PEGylated MoS_2_‐NSs are highly biodegradable and do not accumulate in organs for extended periods.^[^ ^263^, ^264^ ^]^ PEG‐MoS_2_‐NSs have been found to be useful as drug carriers for cancer therapy. The delivery of lipoic acid (LA‐PEG‐MoS_2_) and doxorubicin (DOX‐PEG‐MoS_2_) for combination therapy involving anticancer drug delivery and PTT has synergistic anticancer effects.^[^ ^262^ ^]^ Nanocomposites of MoS_2_ conjugated with Fe_3_O_4_,^[^ ^265^, ^266^, ^267^ ^]^ Bi_2_S_3_,^[^ ^268^, ^269^ ^]^ MoS_2_ nanodots,^[^ ^270^ ^]^ and MoS_2_ quantum dots^[^ ^271^ ^]^ were found to be useful for photothermal, photodynamic and chemical applications. MoS_2_ functionalized with chitosan (CS‐MoS_2_) was reported to possess photothermal‐triggered drug delivery potential to deliver DOX for combined bioimaging (CT), chemotherapy and photothermal therapy applications. The PTCE of the CS‐MoS_2_ ∼ 24.37% in the NIR region allowed heat‐induced controlled drug release of DOX in response to the NIR laser.^[^ ^272^ ^]^

MoS_2_‐NSs show promise as “nanozymes” in cancer theranostics, where a hybrid structure of gold nanobipyramid (AuNBP) coated with MoS_2_ was developed. This smart nanozyme was readily internalized into cancer cells when studied via two‐photon bioimaging, and it exhibited plasmon‐mediated photothermal effects via the generation of ROS at the targeted site.^[^ ^273^ ^]^ MoS_2_‐NSs are rapidly internalized by macrophages, leading to an inflammatory response,^[^ ^274^ ^]^ and compared with pristine MoS2‐NSs, PEG‐MoS_2_‐NSs induce a better inflammatory response.^[^ ^275^ ^]^ Owing to their rapid internalization by macrophages and ability to extend the anti‐inflammatory response, PEG‐MoS_2_ nanoflowers possess high drug loading ability, facilitating controlled and sustained drug delivery, with long circulation times in the blood, which can aid in targeted drug delivery for immunomodulatory applications.^[^ ^276^ ^]^ and facilitated targeted photothermal tumor ablation without inducing any systemic toxicity or side effects.^[^ ^277^ ^]^ MoS_2_‐NSs modulate dendritic cell (DC) maturation and migration upon interaction in a size‐ and dose‐dependent fashion. MoS_2_‐treated DCs also play a vital role in T‐cell activation and the regulation of the adaptive immune response, suggesting their application in nanoimmunotherapy as nanoadjuvants and “nanovaccines”.^[^ ^278^ ^]^ A specific size range of 100–500 nm of MoS_2_‐NSs effectively improved the T‐cell priming capability of DCs regardless of their size and improved downstream immune responses in CD8^+^ cytotoxic T‐ and B‐lymphocytes.

Considering the interaction of MoS_2_‐NSs with macrophages and DCs, PEG‐MoS_2_‐NSs are employed as nanocarriers for drug delivery‐mediated photothermally enhanced cancer immunotherapy.^[^ ^279^ ^]^ Nanosheets easily penetrate through biological membranes and aid in the release of drugs and proinflammatory cytokines,^[^ ^280^ ^]^ and MoS_2_‐CuO composites have been applied in immune‐adjuvant cancer therapy.^[^ ^281^ ^]^

Thus, the above‐discussed research shows evidence that 2D molybdenum‐based nanomaterials have multiple biomedical applications because of their unique interactions with immune cells, photothermal, drug delivery and immunomodulatory effects.

##### 2.3.4.3 Tungsten‐Based Nanosheets

Tungsten is a shiny and silvery‐white metal that has a wide range of applications, from light bulb filaments to electrodes/conventional electronics, the metal/superalloy industry and biomedical applications. Tungsten‐based 2D nanomaterials show promise in biosensing, cancer theranostics and immunomodulation. TMOs of tungsten oxide (WO_x_) possess unique physiochemical properties, and as nanosheets (WO_x_NS), they are highly conductive and exhibit efficiency in the hydrogen evolution reaction (HER) when oxygen vacancies (OVs) are introduced into WO3NS.^[^ ^282^ ^]^ OV‐WO_3_NS, with an enhanced photothermal conversion efficiency (PTCE) ƞ ≈ 41.6% and an up to 96.8% tumor inhibition rate, has a multifunctional role in energy‐based and cancer‐based applications.^[^ ^283^ ^]^ Ultrathin hydrogenated, OV‐WO perovskite NSs are excellent drug delivery platforms for cancer theranostics. These oxygen‐deficient WO_x_‐NSs exhibit pH‐ and photoresponsive drug release behavior with deep tumor penetration in the NIR‐II region, which may aid in the targeted treatment of cancer without instigating any immune response to these nanomaterials.^[^ ^284^ ^]^ Macrophages aid in inhibiting tumor growth, but their long‐term survival in biological systems is a challenge. Thus, combining the photothermal property of WO with the phagocytotic function of macrophages leads to minimal collateral damage to healthy tissue and helps treat cancer. However, WO nanoparticles lack fluorescent properties, which limits their imaging‐guided performance. Hence, in this study, a visually guided therapeutic method was employed in combination with WO_x_ and macrophages as excellent candidates for immunotherapy and PTT both in vitro and in vivo.^[^ ^285^ ^]^

Single‐layer WO‐NSs are used as an efficient method for permanent male sterilization in experimental animals and livestock. This nonsurgical male sterilization method adapts to the photoresponsive‐induced hyperthermia of WO‐NSs. These WO‐NSs are termed “smart photoresponsive sterilants” because of their i) ability to sensitize singlet oxygen, ii) PT/PD effects with benign, negligible toxicity and iii) increased biocompatibility without causing long‐term immune suppression, overcoming adverse effects.^[^ ^286^ ^]^ Defective WO_x_ nanomaterials have been employed as promising solutions to address the challenge of repairing bone defects in patients with diabetes, a condition characterized by disrupted bone immune homeostasis. These WO_x_ nanoribbons, which are immunoengineered as ultrathin nanoribbons with abundant OVs, demonstrate combined functionality: they can moderately scavenge reactive oxygen species (ROS) and exhibit robust photothermal antibacterial properties. Owing to this, WO_x_ nanoribbons prevent the disruption of ROS homeostasis and combat bacterial infections in the repair of diabetic bone defects.^[^ ^287^ ^]^ Doping with metals enhances the physiochemical properties of WO nanoparticles, such as increasing the PTCE, which is characteristic of any given transition metal, and increasing the electrical conductivity. Hence, hybrid WO_x_ nanomaterials, in the form of nanosheets and nanoparticles, find their place in biosensing applications. The increased NIR photothermal conversion of these metal‐doped nanocomposites aids in the treatment of cancer through PTT and PDT and serves as a promising contrast agent for magnetic resonance imaging (MRI), photoacoustic tomography (PAT) and computed tomography (CT) scans.^[^ ^288^ ^]^

PEGylated iron tungsten oxide‐based nanosheets (FeWO‐PEG‐NSs) have been found to be nanoadjuvants for immune therapy and chemodynamic therapies (CDTs), as these nanosheets augment tumor‐infiltrating T cells, provoking a systemic immune response that combines PTT and CDT and causes immunogenic cell death triggered by increased production of ROS. Thus, FeWO‐PEG‐NSs are excellent CDT/PDT/PTT agents for targeting primary tumors and immunotherapeutic agents via the effects of ROS/PTT to inhibit distant tumor growth in NIR‐II biowindows.^[^ ^289^ ^]^ Tungsten oxide‐decorated gold nanoparticle (AuNP‐WO_x_) hybrids aid in anchoring primary antibodies to form chemical bonds between the AuNPs and the –NH_2_ of the antibodies to be used as photoelectrochemical immunosensors for prostate‐specific antigen detection.^[^ ^290^ ^]^ WO_x_ nanoplates exhibit ROS‐mediated anticancer effects, inducing IL‐2, IL‐8 and TNF‐α secretion and altering proapoptotic and antiapoptotic gene expression.^[^ ^291^ ^]^ Functionalized tungsten trioxide (WO_3_) nanosheets were developed as electrochemical immunosensors for cardiac biomarker detection because of their high biomarker loading and selective detection capabilities.^[^ ^292^ ^]^ Most recently, metal single‐atom (SA)‐deposited oxygen vacancy (OV)‐rich WO_3‐x_ nanocomposites with superior sonodynamic therapeutic potential were developed. Sonodynamic therapy employs sonosensitizer‐induced ROS upon ultrasound irradiation to induce tumor cell apoptosis and necrosis to treat cancer. Among various metal SA‐based OV‐ WO_3‐x_, Zhan et al. (2024) reported that copper‐decorated WO_3‐x_ outperformed in terms of ultrasound‐induced ROS production, leading to effective tumor eradication in vitro and in vivo via the modulation of the apoptosis and TNF signaling pathways.^[^ ^293^ ^]^

2D tungsten disulfide (WS_2_) nanosheets are 2D transition metal dichalcogenides (TMDs) with promising electrochemical sensing abilities and are used as photodetectors because of their improved light absorption over a wide UV‒Vis‐NIR (ultraviolet‒visible‐near‐infrared) spectral range.^[^ ^294^ ^]^ An assembly of AuNPs on WS_2_ nanosheets enables highly selective detection of the cardiac marker myoglobin, such as WO_3_ nanosheets, via aptamer‐based SERS detection methods.^[^ ^295^ ^]^ Tungsten disulfide nanosheets (WS_2_‐NSs) aid in monitoring and quantifying the coupling of immunosuppressive drugs with laser desorption ionization mass spectrometry (LDI‐MS), leveraging their strong UV absorbance and ability to generate electron‒hole pairs under laser irradiation to facilitate ionization and desorption.^[^ ^296^ ^]^ Nanocomposites of WS_2_‐graphene and gold nanoparticles are employed in the electrochemical detection of proteins for biosensing applications for the detection of IgE in serum samples as part of an electrochemical immunoassay.^[^ ^297^ ^]^ Because of their high biocompatibility and protein immobilization capability, poly(vinylpyrrolidone)‐tungsten disulfide nanosheets (PVP/WS_2_‐NSs) are used as novel electrochemical immunosensors for the detection of fibroblast growth factor‐21 (FGF‐21). Owing to their protein immobilization capability and high biocompatibility. PVP/WS_2_‐NSs functionalized with gold nanoparticles and metal organic framework (AuNP/MOF) nanozymes are highly electroconductive, assisting in the direct immobilization of antibodies on the electrode surface and emphasizing their role in clinical diagnostics. PVP improved the dispersion and stability of WS_2_‐NSs, and MOF nanozymes helped produce strong electrochemical signals. AuNPs provided a functional surface for bioreceptor immobilization, making this composite highly sensitive, cost effective, rapid and enzyme‐free for the detection of proteins in serum.^[^ ^298^ ^]^ WS_2_‐NSs are exploited for their ability to quench time‐resolved fluorescence efficiently in enzyme‐linked immunosorbent assays, leveraging the ability of bioprobe to absorb WS_2_ through the van der Waals forces created between WS_2_ and nucleobases, leading to quenched fluorescence for analysis. Doping multicolor‐emissive nanoparticles with the bioprobe and WS_2_ led to this time‐resolved fluorometric aptasensor.^[^ ^299^ ^]^ WS_2_‐NSs have also demonstrated excellent antibacterial properties, as they adhere to the surface of bacteria and robustly inhibit cell proliferation, damaging cell membrane integrity and oxidative stress via ROS generation.^[^ ^300^ ^]^

WS_2_ quantum dots (WS_2_QDs) are employed in multimodal (CT/PA) imaging coupled with PTT/RT for the precise detection of tumors and tumor eradication due to their photothermal ability, high NIR absorption and strong X‐ray attenuation. Their ultrasmall size prevents any side effects that can occur due to the injection of the biomaterial.^[^ ^301^ ^]^ Multiwall inorganic WS_2_ nanotubes and fullerene‐like nanoparticles can impair salivary gland function without affecting cell morphology or cell viability when they are taken up by cells and accumulate in cytoplasmic vesicles. This study suggests the potential of these biocompatible nanomaterials for treating oral diseases.^[^ ^302^ ^]^ Another 2D TMD of tungsten is tungsten selenide (WSe_2_) nanosheets, which are employed as ultrasensitive electrochemical biosensors in miRNA detection via an RNA‒RNA hybridization configuration to discriminate and detect any base mismatches in the miRNA sequence.^[^ ^303^ ^]^ Owing to the high PTCE of 35.2% erbium‐doped tungsten selenide (Er‐WSe_2_) in the NIR‐II biowindow, Er contributes to luminescence, and the emission of WSe_2_ in the NIR‐II region shows promising bifunctional bioimaging and PTT effects in cancer theranostics.^[^ ^304^ ^]^

These studies show that tungsten, in its diverse 2D forms, is employed in various fields of immunoengineering, combined with light‐ and heat‐mediated cancer therapy, promising bioimaging and biosensing candidates for clinical therapies.

#### 2.3.5 Group 7 Elements and Reported Immunoengineering Applications

Group‐7 elements include manganese (Mn), technetium (Tc), rhenium (Re), and bohrium (Bh). These elements are abundant in nature and have a hard metallic nature. However, technetium and bohrium have no obvious immunomodulatory effects on their nanosheets in the literature.

##### 2.3.5.1 Manganese‐Based Nanocomposites

Manganese is a brittle, pinkish‐gray metallic element with relatively high chemical reactivity and oxidation capacity. Bivalent manganese ions (Mn^2+^) play a vital role in the innate immune‐cell sensing of tumor antigens and serve as tumor immune activators in promoting the adaptive immune response against tumors.^[^ ^305^, ^306^ ^]^ Photodynamic‐based therapy has been widely studied as a promising noninvasive therapeutic strategy for the treatment of cancer, and 2D manganese dioxide nanosheets have been reported for their application in photodynamic therapy and bioimaging.^[^ ^307^ ^]^ Hao et al reported a pH‐responsive drug delivery application of layered manganese dioxide on the basis of its redox mechanisms for theranostic cancer applications.^[^ ^308^ ^]^ Recently, Mn‐based MOFs have been developed for cancer metalloimmunotherapeutic approaches. The immunostimulatory Mn‐MOFs aid in the infiltration of immune cells into tumors to suppress tumor growth and metastasis.^[^ ^309^ ^]^ Mn was doped with cobalt molybdenum double layered hydroxide (Mn‐CoMo‐LDH) nanosheets to improve the sonodynamic therapeutic potential of the nanosheets.^[^ ^310^ ^]^ This modification effectively modulates ROS production and ROS clearance after alleviating the hypoxic tumor microenvironment to induce tumor cell apoptosis. Furthermore, the doping of Mn served to enhance the MRI contrast ability, further highlighting the multifunctionality of Mn in this nanocomposite. Thus, manganese‐based 2D nanomaterials are employed in biosensing and immunostimulatory‐based clinical cancer theranostics, owing to the above‐discussed research.

##### 2.3.5.2 Rhenium‐Based Nanosheets

Rhenium is a silvery metallic element with relatively good corrosion resistance. Rhenium disulfide (ReS_2_)‐based materials obtained via liquid exfoliation were reported as theranostic nanoagents for photoacoustic/computerized tomography imaging and PTT applications.^[^ ^311^ ^]^ The obtained 2D ReS_2_ nanosheets (ReS_2_‐NSs) have an average lateral size between 20–100 nm and a layer thickness of 1.5–5 nm. As a light‐mediated therapeutic agent, the synthesized PVP‐capped ReS_2_‐NSs exhibited enhanced tissue penetration and minimal invasion properties due to the relatively high Z content of rhenium (*Z *= 75) and photoacoustic activity. With high near‐infrared absorption and a PTCE of approximately 79% (other TMDs in the literature possess a PTCE of approximately 30%),^[^ ^272^, ^312^, ^313^ ^]^ a tumor elimination rate of up to 100% was achieved. Briefly, 5 concentrations of the as‐designed biocomposite were tested at 10, 20, 40, 80, and 120 µg mL^−1^; the results revealed rapid and dose‐dependent temperatures (from 6.7 to 46.1 °C) after 10 minutes of irradiation, indicating effective photothermal activity. In addition, no significant cytotoxic effects, organ damage or inflammation were observed.

The application of a rhenium‐based immunoengineered system for tumor targeting and stimuli‐responsive drug delivery combined with chemo‐photothermal therapy has been reported.^[^ ^314^ ^]^ In this study, bovine serum albumin‐assisted exfoliation of thin 2D layered rhenium‐disulfide resulted in efficient photothermal properties under laser irradiation at 808 nm. This treatment resulted in photothermal activity at a pH of ≈6.5 and facilitated the release of the anticancer drug, with 55% resveratrol after six cycles of 5 min of NIR irradiation. Rhenium‐based compounds are promising for targeted anticancer therapies because of their relatively high oxidation states and heterometallic complex assemblies.^[^ ^315^ ^]^ Owing to the cytotoxic and phototoxic effects of these rhenium‐based materials on cancer cells reported in preclinical studies, these materials act through DNA binding, mitochondrial effects, enzyme inhibition, and oxidative stress regulation through ^1^O_2_ and CO release mechanisms. Rhenium radioisotopes have potential in the treatment of inflammatory joint damage and bone tissue damage. Taken together, these findings suggest that rhenium‐based immunoengineered nanosystems offer selective anticancer and immunomodulatory effects, meriting further investigation.

#### 2.3.6 Group 8 to Group 10 Elements and Reported Immunoengineering Applications

The elements of group 8 of the periodic table are iron (Fe), ruthenium (Ru), osmium (Os), and hassium (Hs). Only iron has notable applications as an iron‐based nanomaterial and oxide composite for diagnosis, antibacterial activity, and treatment of bacterial infectious diseases.^[^ ^316^ ^]^ Metal ions modulate immune cell function within the complex tumor microenvironment via the transmission of intracellular and extracellular signals. The application of nanometals for tumor immunotherapy has proven effective in synergistically enhancing the T‐cell‐mediated immune response against cancer.^[^ ^317^ ^]^ In this context, iron‐based LDHs have been developed to enhance ferroptosis‐induced cancer immunotherapy. Fe/Al‐LDHs are immunoengineered to selectively degrade in acidic tumor environments and release iron ions to induce programmed cell death in tumor cells while simultaneously promoting the polarization of macrophages toward the M1 phenotype. These findings underscore the potential of nanoiron to significantly improve the efficacy of tumor immunotherapy.^[^ ^318^ ^]^

^The^ Group 9 elements of the periodic table contain all the transition metals in the d‐block. These compounds include cobalt (Co), rhodium (Rh), iridium (Ir) and meitnerium (Mt). Rh is an extremely rare, carcinogenic, precious metal, and Ir is the second‐densest silvery white metal in nature. Both rhodium and iridium are platinum group metals (PGMs). Except for cobalt (Co), to date, no significant reports are available on the immunomodulatory applications of 2D Rh, Ir, and Mt‐based nanosheets. In Groups 9–12, there are some elements with unknown properties, termed chemically uncharacterized elements, as they are rare, and radioactive synthetic elements are not found in nature. The elements that fall into this category are meitnerium (Group 9), darmstadt (Group 10), roentgenium (Group 11), and copernicium (Group 12), with few reports on their 2D synthesis and unexplored biological properties.

##### 2.3.6.1 Cobalt‐based Nanosheets

Cobalt is used industrially and medically for irradiation and is an essential component of vitamin B12, assisting in mature red blood cell formation and extending a protective role to the nervous system. 2D cobalt‐based nanomaterials are employed in cancer therapy. For example, layered cobalt hydroxide nanosheets (Co(OH)_2_‐NSs) were developed for treating ovarian cancer, where Co(OH)_2_ offered more effective anticancer and antioxidant activity than did cobalt acetate (as the precursor).^[^ ^319^ ^]^ As self‐therapeutic nanomaterials, Co(OH)_2_‐NSs exert selective anticancer effects, offering an alternative to existing chemotherapies. Compared with FDA‐approved cisplatin drugs, Co(OH)_2_‐NSs are less toxic to normal cells and increase the degree of cellular apoptosis. Localization and cellular internalization assays indicated effective uptake and escape from lysosomal degradation. Iron sulfide/cobalt sulfide nanosheets have been employed for hypoxic tumor therapies because of their light‐assisted intracellular photocatalytic oxygen generation bioactivity.^[^ ^320^ ^]^ Furthermore, a composite nanosheet comprising iron, cobalt, and polyethylene glycol (FeS_2_/CoS_2_@PEG) was formulated owing to the redox cycles between iron and cobalt (Fe^3+^/Co^3+^ and Fe^2+^/Co^2^). The nanocomposite exhibited high absorption in the NIR‐II biowindow with a PTCE of 50.5%, with high redox potential because it readily oxidizes water, forming O_2_ and reducing the dissolved O_2_ to produce ROS at the tumor site. The nanocomposite relieved tumor hypoxia by mimicking peroxidase and catalase activities to decompose endogenous hydrogen peroxide and served as a glutathione oxidase to disrupt ROS homeostasis and facilitate intracellular oxidative stress in the tumor area.

Additionally, cobalt‐based LDHs have been employed in treating cancer through sonodynamic therapy. These LDHs act as sonosensitizers, which, when activated by ultrasound, produce promising amounts of ROS for treating deeply located tumors via the induction of cancer cell apoptosis. Upon undergoing phase transformation, CoW‐LDH nanosheets were found to act as highly efficient sonosensitizers for sonodynamic therapy.^[^ ^105^ ^]^ Furthermore, the doping of Mn with CoMo‐LDH nanosheets alleviated the hypoxic conditions in the tumor microenvironment and synergistically promoted the sonodynamic therapeutic potential of the Cobased LDH nanosheets.^[^ ^310^ ^]^ Thus, the antioxidant and anticancer effects of cobalt‐based nanomaterials support their efficient use in synergistic antitumor immune, photothermal, photodynamic, sonodynamic and chemotherapeutic applications.

Group 10 of the period table consists of d‐blocks, silvery white transition metals, namely, nickel (Ni), palladium (Pd), platinum (Pt), and the chemically uncharacterized darmstadtium (Ds). Nickel is a hard, ductile element with good conductivity and hydrogen absorption properties. Crystalline ultrathin nickel‐based nanosheets have been fabricated for various chemical, electrical, and optoelectronic applications; however, their biological use has not been well reported. However, layered nickel‐graphene composites have demonstrated desirable electrocatalytic properties and are used as biosensors.^[^ ^321^ ^]^ Platinum is a lustrous, ductile element with corrosion‐resistant properties. Platinum‐based nanosheets are synthesized in different forms, including bulk crystals, colloids, and thin films, for diverse electrochemical and detection‐based approaches. Platinum compounds such as cisplatin and carboplatin are used for the treatment of cancer. Despite the biologically inert nature of platinum, soluble, halogenated platinum salts potentiate platinum‐salt hypersensitivity upon direct contact and exposure to the body, limiting their use in immunoengineered biological systems.

##### 2.3.6.2 Palladium‐Based Nanocomposites

Palladium is a shiny metallic element that is naturally available in some geological reigns but is often extracted as a byproduct of nickel refinement. In medicine, palladium‐based nanostructures have been studied for use in photothermal therapy as antimicrobial and antitumor agents. Ultrasmall palladium nanosheets (Pd‐NSs) have been shown to possess suitable light‐sensitizing properties,^[^ ^322^ ^]^ converting light into heat upon laser irradiation, with a PTCE of 52%. Pd‐NSs functionalized with reduced glutathione possessed enhanced biodistribution in the blood and accumulation at a relatively high rate in tumors, with ultrasmall sizes (<10 nm) allowing a unique renal clearance route. Thus, they are effective photothermal agents with favorable biodistribution and clearance. Jiang et al reported the effect of 2D palladium‐containing injectable scaffolds for synergistic chemo‐photothermal therapy.^[^ ^323^ ^]^ These hydrogels showed high photothermal efficiency, stability over the conventional matrix of similar compositions, high drug loading capacity and sustained release of doxorubicin under NIR irradiation to inhibit tumor metastasis. These findings demonstrate the use of palladium‐based nanomaterials to develop novel anticancer therapies with enhanced biodistribution and clearance without causing a nanomaterial‐induced immune response.

#### 2.3.7 Group 11 Elements and Reported Immunoengineering Applications

Group 11 of the periodic table. is composed of copper (Cu), silver (Ag), gold (Au) and roentgenium (Rg). Silver and gold are considered noble metals, and roentgenium is a radioactive synthetic element with no known biological role. All the metallic elements in this group mostly occur in oxidation stages ^+1^ and ^+2^. The immunomodulatory applications of Cu, Ag and Au are discussed below.

##### 2.3.7.1 copper‐based nanocomposites

Copper‐based nanosheets have demonstrated good electronic and optoelectronic properties. However, despite these well‐established favorable properties, their biological applications have remained largely unexplored. This research gap can be attributed to concerns regarding the high electrical and thermal conductivity of copper‐based materials, as well as the potential release of free copper ions or ligand‐assisted copper ions in biological environments over time.^[^ ^324^, ^325^ ^]^ Nonetheless, recent investigations have begun overcoming these challenges, paving the way for renewed interest in testing copper‐based nanomaterials in biomedical applications, particularly in the field of cancer immunotherapy. Guoqing et al. (2024) developed copper ion‐doped LDH (Cu‐LDH) nanoparticles for cancer immunotherapy via their dual ability to trigger cuproptosis through abnormal lysosomal aggregation within tumor cells and induce lysosomal rupture. Cuproptosis is a regulated form of cell death triggered by excess copper, and pyroptosis is a form of cell death triggered by inflammation‐associated proinflammatory signals. Researchers have leveraged this copper‐induced cell death and immune cascade for cancer therapy. Cu^2+^ ions facilitate therapeutic efficacy by promoting cuproptosis and pyroptosis, leading to the blockade of autophagy to effectively suppress tumor cell growth and increase the efficiency of tumor therapy.^[^ ^326^ ^]^ In addition, dual gadolinium and copper LDH (GdCu‐LDH) nanoparticles have been developed owing to their high magnetic resonance imaging (MRI) contrast properties for use in accurate cancer diagnostics.^[^ ^327^ ^]^ Owing to the efficiency of synergistic sonodynamic therapy (SDT) and cuproptosis in cancer therapy, Cooper‐based LDH nanosheets have been used. The introduction of Cu^2+^ ions into the tumor microenvironment (TME) amplified oxidative stress and enhanced the sonodynamic therapeutic potential by triggering immunogenic cell death and enhancing dendritic cell maturation. Overall, this strategy of Cu^2+^ ion‐mediated SDT/cuproptosis was found to be effective in promoting antitumor immune outcomes in the treatment of cancer.^[^ ^328^ ^]^ Copper‐based LDH composed of cobalt, copper and molybdenum loaded on *Lactobacillus acidophilus* has been reported to elicit sufficient photodynamic (PDT) activity in the TME, leading to ROS‐induced cell apoptosis and tumor eradication.^[^ ^329^ ^]^ These studies show that copper‐based nanomaterials, particularly LDHs, are promising candidates for cancer immunotherapy because of their unique ability to induce pyroptosis, enhance SDT/PDT and provide improved diagnostic capabilities.

##### 2.3.7.2 Silver‐Based Nanocomposites

Silver nanomaterials have a long history of antimicrobial applications in combating different types of invasive and noninvasive microorganisms for the treatment of pathogenic diseases. Despite concerns about their long‐term toxicity, extensive research has focused on optimizing their therapeutic efficacy. For example, Li et al. reported the synthesis of hybrid silver nanosheets (Ag‐NSs) as efficient antibacterial membranes.^[^ ^330^ ^]^ Compared with silver ions, silver nanoparticles (Ag‐NPs) offer superior controllability, long‐term antibacterial effects and antibiofouling activity. They elicit antibacterial effects by interacting with bacterial amino acids to inhibit their intracellular proteases and subsequently prevent DNA replication. In this context, Ag‐NPs have been studied for antibacterial, antiviral, anticancer, and anti‐inflammatory applications, including immunomodulation and gene regulation.^[^ ^331^, ^332^, ^333^, ^334^ ^]^ Ag‐NPs exhibit broad‐spectrum antiviral activity against respiratory pathogens by targeting viral glycoproteins, preventing pathogen entry, reducing pathogen replication, decreasing the production of proinflammatory cytokines and chemokines and increasing neutrophil recruitment and activation.^[^ ^334^ ^]^ Studying Ag‐NP‐induced cytokine release is essential for its use in clinical studies, as Ag‐NPs interact with cells, and their toxicity profile induces cytokine release, predicting an immune response. An in‐depth study by Bae, Jiwon, et al. via mass spectrometry revealed the size‐dependent Ag‐NP‐mediated inflammatory response in human peripheral blood mononuclear cells (hPBMCs), providing guidance for the design of appropriate nanomaterials for future therapeutics.^[^ ^335^ ^]^ These results expand the scope of the study and evaluation of the immune toxicity, long‐term safety and environmental compatibility of silver nanostructures.

##### 2.3.7.3 Gold‐Based Nanocomposites

Gold is one of the most ductile and malleable metallic elements, and gold‐based nanomaterials are widely utilized in biomedical and clinical applications, including biosensors, implants, drug delivery, antimicrobial therapy, anticancer, and regenerative medicine.^[^ ^335^, ^336^ ^]^ Since its early development, engineering of gold nanoparticles (Au‐NPs) has advanced, leading to the development of different nanoforms, such as nanorods, nanoparticles, nanotubes, and thin nanofilms. Inflammatory diseases cause an imbalance between M1 and M2 macrophages. Gold nanostructures possess suitable biocompatibility at controlled doses to aid in the treatment of inflammatory diseases.^[^ ^337^ ^]^ These compounds effectively interact with macrophages because of their ability to track contrast and highly active surface for targeted delivery and elicit macrophage‐mediated inflammation.^[^ ^338^ ^]^ These compounds possess excellent therapeutic features for immune modulation, such as inhibiting macrophage responses, preventing macrophage recruitment, and modulating M1/M2 polarization.^[^ ^337^, ^339^ ^]^ Owing to the interaction of gold nanoparticles with macrophages, they can be used in inflammation‐related disease imaging combined with their photothermal ability, serving as effective macrophage modulators in the immunotherapy of inflammatory diseases and photothermal therapy in cancer.

Elsewhere, gold nanomaterials have emerged as potential treatments for rheumatoid arthritis, a common autoimmune inflammatory disease characterized by destruction of joint cartilage. This is driven by complex interactions between inflammatory mediators such as TNF‐a, IL‐1b, cyclooxygenase‐2 and nitric oxide.^[^ ^340^ ^]^ In this context, the immunomodulatory mechanism of Au‐NPs in a collagen‐induced arthritis rat model was studied. After the induction of arthritis, the animals received 20 µg kg^−1^/body weight of the Au‐NPs for approximately 3 weeks. Nanogold treatment significantly reduced the levels of TNF‐a, IL‐1b, COX‐2 and NF‐κB. Au‐NPs decreased oxidative stress and inflammatory mediator levels and attenuated the imbalance between oxidant and antioxidant status close to normal. Au‐NPs inhibited the activation of NF‐kB, reducing proinflammatory cytokines and ultimately preventing cartilage destruction. Compared with those of the control animals, the histological findings revealed a remarkable decrease in bone erosion and inflammation in the Au‐NP‐treated animals. The application of nanogold‐based composites to fabricate biosensor electrodes for the detection of SARS‐CoV‐2 has been recently reported.^[^ ^341^ ^]^ This biosensor is composed of gold, graphene, a polymeric matrix, and immunoglobulins, and specific SARS‐CoV‐2 antibodies are attached to designed electrodes. Changes in electrochemical signals indicate virus detection, serving as a bioelectronic tool for monitoring health/infection status.

Thus, owing to the inherent photothermal, antioxidant and immunomodulatory properties of gold nanoforms in ablating tumors, reducing oxidative stress and modulating macrophages, respectively, 2D gold is considered the finest candidate for immunoengineering‐based cancer and regenerative medicine.

#### 2.3.8 Group 12 Elements and Reported Immunoengineering Applications

Group 12, also called the zinc group, contains zinc (Zn), cadmium (Cd), mercury (Hg), and copernicium (Cn). Mercury is widely used in medical devices such as thermometers, sphygmomanometers and mercury‐containing derivatives and is employed in antiparasitic, anti‐inflammatory, antisyphilis, antipruritic, antiseptic drugs and dental amalgams. However, the toxicity of mercury to humans is still debatable, which limits its use as a 2D nanomaterial for various biomedical applications. Copernicium, a synthetic element formed by fusing lead and zinc atoms, has no biological role because of its limited availability and radioactivity. The biological role of zinc and cadmium in biomedical applications is discussed below.

##### 2.3.8.1 Zinc‐Based Nanocomposites

Zinc, a lustrous, bluish‐white metallic element, plays antioxidant and anti‐inflammatory roles in the human body by reducing oxidative stress, which leads to chronic inflammation. Like nanogold, nanozinc also adapts to phagocytic uptake by macrophages to target inflammation and has potential as an anti‐inflammatory agent. Recently, 2D metal‒organic frameworks (MOFs) were designed on the basis of zinc cations and tetrakis(4‐carboxyphenyl) porphyrin as the organic linker. These Zn‐MOFs were surface modified with polyethylene glycol as potential drug delivery carriers for combined chemo‐photodynamic therapy.^[^ ^342^ ^]^ The developed anticancer system possesses significantly greater light‐triggered singlet oxygen generation properties with efficient doxorubicin drug‐loading capacity due to the photodynamic properties and large surface areas of these zinc‐containing nanosheets for targeted delivery. They also exhibited increased biocompatibility and antitumor efficacy for effective tumor retention upon intravenous injection. Zn‐LDHs have been used as highly efficient photosensitizers for photodynamic therapy to treat cancer by penetrating deep into the tissue and inducing cancer cell damage.^[^ ^106^ ^]^ Hence, nanozinc is employed in immunomodulatory and anticancer therapeutics.

##### 2.3.8.2 Cadmium‐based Nanocomposites

Cadmium, a lustrous, malleable, ductile element, has properties similar to those of zinc, as it is present in small traces in zinc ores and is a byproduct of zinc production. Cadmium‐based materials in thin film and solid powder forms are used to fabricate different biomedical sensors and electrodes.^[^ ^343^, ^344^, ^345^ ^]^ Although nano‐ and quantum‐sized materials of cadmium have gained attention, concerns regarding their long‐term safety persist due to their ability to release toxic cadmium ions and accumulate in different organs (spleen, liver, and kidney) through the bloodstream through reversible cellular uptake, promoting systemic inflammation and toxicity to the intestine and gut microbiota.^[^ ^346^, ^347^ ^]^ Studies on the biosafety, biodistribution and pharmacokinetics of these quantum dots are important for the future development of biologically safe cadmium‐based nanomaterials. For example, Golovine et al reported that cadmium could effectively downregulate the expression of X‐linked inhibitor of apoptosis protein (XIAP) in prostate cancer cells.^[^ ^348^ ^]^ Prostate has the highest amount of cadmium accumulation, and in prostate cancer patients, cadmium levels are high both in circulation and in resident tissues, leading to increased risk of inflammation. Cadmium downregulates the expression of IAPs, inhibiting apoptosis at the posttranscriptional level via an NF‐κB‐independent proteasome‐mediated mechanism, which coincides with TNF‐α‐mediated apoptosis. Further investigations are needed to establish the pro‐/anti‐inflammatory effects of cadmium for its use as a two‐dimensional nanomaterial.

In conclusion, the above‐discussed sections highlight the significant potential of Group 3 to Group 12 transition metal‐based 2D nanomaterials in immunoengineering applications in diverse forms, such as nanoparticles, nanosheets, nanofilms and quantum dots. The physiochemical properties of these elements dictate their synthesis, which is tailored for specific applications, including drug delivery; photothermal, photodynamic and sonodynamic cancer therapies; and immunomodulation. Owing to their outstanding bioelectronic and thermal properties, these materials selectively target tumor tissues via preferential heating, enabling oxidative stress‐induced tumor ablation. Additionally, immune cell modulation can be facilitated by demonstrating selective toxicity to tumor cells while maintaining biocompatibility with normal cells for anticancer, tissue regeneration and inflammatory disease treatment. As research advances, the potential of transition metal‐based 2D nanomaterials has increased, offering promising avenues for cancer theranostics and regenerative medicine.

### 2.4 Group‐13 to Group‐17 Elements and Reported Immunoengineering Applications

Groups 13–17 include posttransition elements categorized into boron, carbon, nitrogen, chalcogen, halogen and noble gas groups, respectively, which contain metals, nonmetals and at least one metalloid. Like the chemically uncharacterized radioactive metals discussed in the previous groups, the following elements are synthetic radioactive elements with no biological role: nihonium (Group‐13), flerovium (Group‐14), moscovium (Group‐15), livermorium (Group‐16), tennessine (Group‐17), and organesson (Group‐18). Below are groupwise discussions on 2D materials derived from posttransition elements and their immune‐based applications.

#### 2.4.1 Group 13 Elements and Reported Immunoengineering Applications

Group 13, known as the boron group, includes boron (Br), aluminum (Al), gallium (Ga), indium (In), thallium (Tl), and the chemically uncharacterized nihonium (Nh). Indium is the softest, malleable, silvery metal with remarkable semiconductor and optoelectronic properties. 2D indium nanosheets maintain metallic properties under strain, and their buckled forms can transition from an indirect semiconductor to a metal. Hence, adding indium to alloys enhances their mechanical properties, such as microhardness, wear resistance and compressive strength, yielding highly corrosion‐resistant alloys for orthopedic applications.^[^ ^349^ ^]^ Studies have revealed that single layers of a new 2D indium (indiene) possess metal‐like properties that enhance their electronic conductance and optical absorption for potential nanomedicine and anticancer applications.^[^ ^350^ ^]^ However, given the high toxicity of indium and the lack of knowledge on its direct effects on human health, no obvious studies have reported its intrinsic biomedical applications. 2D thallenenes possess optoelectronic properties comparable with those of other noncommon Xene bulk nanosheets^[^ ^351^ ^]^ but lack biomedical studies. The reported 2D immunomodulatory applications of 2D nanoforms of B, Al and Ga are discussed below.

##### 2.4.1.1 Boron‐Based Nanocomposites

Boron, the only nonmetal in group 13, is found in minerals such as borate, kernite, borax, colemanite, and ulexite. The two‐dimensional form of boron, borophene, is a type of Xene. Xenes are novel 2D monoelemental materials with unique physicochemical, electronic, and optical properties. Borophene surpasses its preceding translational nanomaterials because of its high‐performance diagnostic, sensing, optical, magnetic and electrical properties, and it is widely used in energy storage, biosensors, bioimaging and theranostic applications. Borophene's alluring application in artificial intelligence (AI), internet of medical things (IoMT)‐assisted biomedical devices, shows its futuristic prospects in next‐generation healthcare management.^[^ ^352^ ^]^ PEGylated borophene nanosheets (PEG‐B‐NSs) serve as a photonic drug delivery platform with high drug‐loading capacity and a high PTCE of 42.5%. Photothermal irradiation triggers drug release, with multimodal imaging and thorough tumor ablation, and these nanosheets show good tracking and biocompatibility without significant cytokine response or tissue injury.^[^ ^28^ ^]^ 2D borophene flakes integrated with plasma proteins to synthesize functional nanosheet‒corona complexes promoted a macrophage‐mediated immune response; the plasma corona promoted the uptake of the nanomaterial by phagolysosomes.^[^ ^353^ ^]^ Proteomic analysis revealed that plasma proteins could effectively change the surface identities of B‐NSs, with a binding efficiency of 46.5%. and consequently regulate macrophages by reducing the production of cytokines, such as TNF‐α, and increasing the level of ROS. Owing to their excellent drug loading properties, the anticancer drug fluorouracil‐loaded B‐NSs have been developed to increase drug bioavailability and overcome adverse effects, such as neurotoxicity, cardiotoxicity, and myelotoxicity.^[^ ^354^ ^]^ Thus, B‐NSs possess exceptional drug loading capability, macrophage regulation ability and photothermal properties for future nanoenabled immune‐cancer theranostics.

##### 2.4.1.2 Aluminum‐Based Nanocomposites

The abundance, inertness, and physicochemical properties of aluminum, a lightweight, soft posttransition metal, and its derivatives make them valuable in various engineering applications. Owing to its malleability, aluminum is used in diagnostic, surgical and other medical devices. Aluminum hydroxide‐based substances are considered “generally regarded as safe” (GRAS) in various foods, cosmetics, human vaccines, and preclinical applications.^[^ ^355^, ^356^, ^357^ ^]^ In particular, aluminum‐containing adjuvants increase vaccine efficacy by increasing antigen biodistribution for prolonged periods, leading to efficient stimulation of the host immune system.^[^ ^358^ ^]^

In cancer therapy, aluminum nanosheets affect the tumor microenvironment and potentiate the antitumor effects of cancer drugs. Nanoaluminum oxide hydroxide‐based materials (boehmite) are promising nanocontainers for anticancer applications.^[^ ^359^ ^]^ Recently, 2D layered aluminum sheets in the fields of nanomedicine and cancer therapy were reported.^[^ ^360^ ^]^ Cancer cells are sensitive to changes in the extracellular ion concentration; metallic nanomaterials such as aluminum hydroxide nanosheets (aloohenes), which have high bioactivity and high positive charge, disturb the tumor microenvironment (TME). The desirable microstructural stability, optical adsorption, and surface charge properties of aloohene potentiate its antitumor effect and can be used in combination with other anticancer drugs. The large surface area and positive charge of aloohene lead to the selective adsorption of anionic species in the extracellular environment, leading to ionic imbalance in the perimembranous space in the cells of the TME. This resulted in a 30–37% decrease in the proliferation of the 3 tested cancer cell lines. Aloohene nanosheets interact with tumor cells and the TME stromal compartment by decreasing tumor cell viability and proliferation. This inhibits the progression of murine melanoma when loaded with a low dose of doxorubicin. These reports demonstrate the synergistic therapeutic role of nanoaluminum in cancer immune therapy.

##### 2.4.1.3 Gallium‐Based Nanocomposites

Gallium possesses remarkable chemical stability, loading capacity, optical low‐power‐consumption, and phase‐change memory properties; thus, it has applications in electronics, robotics, product design, medical devices such as thermometers, barometers and biomedicine. A recent study by Kochat et al. developed a gallium‐based Xene with a honeycomb‐lattice structure of monolayered gallium sheets (gallenene),^[^ ^361^ ^]^ opening avenues for diagnostic, imaging, and optical‐therapeutic strategies and other biomedical applications.^[^ ^362^ ^]^

Antibacterial nanomaterials have limited effectiveness due to their cytotoxicity; gallium‐based nanocomposites overcome this limitation and elicit enhanced antibacterial effects by preventing implantation‐induced infections, such as those caused by *Pseudomonas aeruginosa*, a common rod‐shaped bacteria that triggers a cascade of inflammatory responses. In this context, a nanocomposite consisting of graphene oxide (GO) and gallium nanoparticles (GaNPs) was developed to treat implant‐associated bacterial infection in the treatment of bone fractures.^[^ ^363^ ^]^ GO‐GaNPs demonstrated enhanced therapeutic efficacy and biosafety when tested in in vitro and in vivo infectious microenvironments. The increased osteogenic ability of the GO‐GaNPs is attributed to the GO enhancing osteoblastogenesis and the GaNPs inhibiting osteoclastogenesis. Together, these synergistic nanosystems aid in improving bone regeneration, eliminating biofilm formation and overcoming the complications imposed by implant‐related osteomyelitis. The relatively low toxicity of gallium makes it suitable for replacing liquid metals such as mercury in biomedical applications.^[^ ^364^ ^]^ Additionally, gallium‐based liquid metals have been developed and studied for their antimicrobial properties.^[^ ^365^ ^]^

By utilizing the antibacterial properties of gallium, the wound healing properties of gelatin and the anti‐inflammatory properties of quercetin, gallium‐based gelatin nanoparticles loaded with quercetin (QCT@GNPs‐Ga) were synthesized for wound healing applications.^[^ ^366^ ^]^ Gallium ions, which mimic iron, enter bacterial cells to disrupt iron metabolism, enhancing their antibacterial effects. QCT@GNPs‐Ga modulates inflammation by regulating macrophage polarization from the M1 phenotype to the M2 phenotype through the TGF‐β/Smad pathway, reducing scar formation and enhancing healing. Gallium‐based metal‒organic frameworks (Ga‒MOFs) were developed to overcome the clinical challenges associated with biofilm formation. Ga‐MOFs disrupt bacterial iron metabolism and function as antibiotic carriers that penetrate bacterial biofilms, inhibiting bacterial pyroptosis and alleviating inflammation.^[^ ^367^, ^368^ ^]^ Gallium is also known to disrupt iron homeostasis in immune cells, resulting in its anti‐inflammatory effect. However, Chengchen et al. reported that gallium nanodroplets (Ga‐NDs) have immunomodulatory effects without hindering iron homeostasis.^[^ ^369^ ^]^ This is achieved by the molecular interaction between Ga‐NDs and macrophages, which selectively inhibits nitric oxide production without affecting proinflammatory mediators (verified by the nondisrupted accumulation of ROS, IL‐6, TNF‐α, and NO). These findings highlight the extraordinary application of nanogallium: a) in resolving clinical dilemmas related to bacterial infection, b) in tissue engineering due to its regeneration potential and c) in immunoengineering owing to its anti‐inflammatory properties.

In conclusion, the group of 13‐2D nanocomposites of boron, aluminum and gallium extend their biomedical application, particularly in cancer therapy and immunomodulation, by influencing the TME and immune responses, respectively. They show similarities in drug loading abilities with inherent anticancer properties and regulate macrophage‐mediated inflammatory responses, leveraging their application in wound healing and antibacterial treatments.

#### 2.4.2 Group‐14 Elements and Reported Immunoengineering Applications

The group‐14 elements consisted of carbon (C), silicon (Si), germanium (Ge), tin (Sn), lead (Pb), and the extremely radioactive synthetic element flerovium (Fl). Lead is widely used in industrial applications and in the nuclear industry as a protective shield to effectively absorb electromagnetic radiation. Despite its industrial applications, lead has no biological use because it is highly toxic, accumulates in the body, depletes antioxidants and increases systemic inflammation, especially in the liver, causing chronic inflammation and fibrosis.^[^ ^370^ ^]^ Animal studies have shown lead‐induced hepatoxicity, characterized by reduced levels of antioxidants such as glutathione and increased levels of proinflammatory cytokines such as tumor necrosis factor (TNF‐α), interferon (INF‐γ), and interleukins such as IL‐1β, IL‐6, and IL‐8.^[^ ^371^ ^]^ Lead in urine serves as a breast cancer biomarker, as lead disrupts the endocrine system, affecting estrogen and inducing tumors by inhibiting apoptosis and abnormal signal transduction,^[^ ^372^ ^]^ and promotes inflammation via the regulation of interleukin 1 receptor‐associated kinase 1, which is a regulator of the NF‐κB pathways involved in mammary gland malignancy.^[^ ^373^ ^]^ The development of thin 2D lead‐based nanosheets is still in its infancy^[^ ^374^ ^]^ and is mostly employed in optoelectronic applications. Owing to the toxicity and proinflammatory properties of lead, lead‐based 2D nanomaterials are not preferred for biomedical applications. The immunomodulatory applications of C, Si, Ge and Sn are discussed below.

##### 2.4.2.1 Carbon‐Based Nanocomposites

Carbon is one of the most abundant elements with unique physicochemical properties and can form new biologically salient compounds with superior characteristics. Owing to this, 2D carbon nanomaterials have gained significant popularity because of their enhanced bioactivity and tunable physicochemical and biological properties. Interestingly, the physical interaction of 2D biomaterials with the cellular membrane leads to selective cellular uptake, and tissue penetration is considered the primary basis for their biological and immunological properties.^[^ ^375^ ^]^ The surface charge of 2D carbon‐based nanosheets significantly affects their biocompatibility, cellular uptake activity, and accumulation of phospholipids on their large surface area.^[^ ^376^ ^]^

Graphene, a 2D single layer of carbon arranged tightly in a hexagonal honeycomb lattice, has revolutionized the field of nanomedicine because of its tunable immunomodulatory properties. Graphene and its derivatives elicit their immunomodulatory effects by stimulating macrophages and activating monocytes, releasing growth factors, regulating inflammation, promoting the differentiation of mesenchymal stem cells to osteoblasts to maintain bone homeostasis and enhancing regeneration.^[^ ^377^ ^]^ Specifically, GO‐NSs induce bone regeneration by stimulating macrophages toward the M1 phenotype to release proinflammatory cytokines, creating a favorable osteoimmune microenvironment for osteogenesis with increased levels of osteogenic factors for the osteogenic differentiation of stem cells. Xue et al investigated the effects of graphene oxide nanosheets (GO‐NSs) on the inflammatory properties of mouse macrophages (RAW‐264.7 cell lines) toward enhanced osteogenesis,^[^ ^378^ ^]^ where GO‐NSs increased the levels of the proinflammatory cytokines IL‐1β, IL‐6, INFγ, and TNF‐α and the osteogenic factors myeloid differentiation factor 88, BMP‐2, oncostatin M, and VEGF via the oncostatin M and NF‐κB‐VEGF signaling pathways.^[^ ^379^ ^]^

Graphene‐based nanomaterials have been shown to possess immunomodulatory properties because their intrinsic ability to suppress immune cells^[^ ^377^, ^378^, ^380^ ^]^ after being internalized through passive diffusion does not affect cell viability or induce an inflammatory response, leading to cell death.^[^ ^381^ ^]^ In addition, carbon quantum dots at nontoxic doses aided in the production of anti‐inflammatory T helper 2 cytokines, inhibited the production of proinflammatory T helper 1 cytokines, affected dendritic cell functions, suppressed T regulatory cells, downregulated reactive oxygen species (ROS) generation, influenced NF‐κB translocation and mTOR activity and increased autophagy.^[^ ^382^, ^383^, ^384^ ^]^

The increasing importance of graphene in nanotechnology has created the need to understand the underlying immunomodulation^[^ ^385^ ^]^ and molecular mechanism of interaction via molecular stimulation.^[^ ^386^ ^]^ To reduce its toxic effects and increase its biological effects, the functionalization of graphene nanomaterials with polymers such as polyethylene glycol (PEG) and polyethyleneimine (PEI) has been reported. By being readily internalized by macrophages, functionalization enhances biocompatibility and immunomodulatory properties, with the PEI composite accumulating in both endosomes and the cytoplasm and the PEG composite in endosomes.^[^ ^387^ ^]^ The immunomodulatory activities of graphene and carbon‐based nanomaterials have received considerable attention for cardiac tissue regeneration. Han et al reported the dual immunomodulatory role of GO‐NSs in macrophage‐targeting/polarizing effects. The dual roles of GO‐NSs are a) to act as antioxidants in reducing inflammation via macrophage polarization from the M1 to M2 phenotype and attenuating intracellular ROS generation and b) to function as nanocarriers of IL‐4 plasmid DNA for the propagation of M2 macrophages and enhanced release of cytokines favorable for cardiac repair. This immunoengineering approach remarkably improved ROS scavenging, fibrosis mitigation, and cardiac functions with desirable bioactivity to increase the survival and proliferation of transplanted stem cells under hypoxic conditions. Park et al reported the antioxidant responses of MSC‐encapsulated graphene oxide/alginate hydrogels, where MSCs improved cardiac maturation via paracrine signaling and alginate microgels decreased the infract size; together, they served as an effective platform for stem cell delivery and a protective antioxidant system.^[^ ^388^ ^]^

Functionalized carbon nanotubes (CNTs) modulate immune responses by interacting with macrophages, lymphocytes, dendritic cells, phagocytic cells and the complement system.^[^ ^385^ ^]^ The surface charge and molecular patterns of CNTs can influence complement activation, which in turn modulates phagocytosis and cytokine responses, potentially dampening proinflammatory responses.^[^ ^389^ ^]^ In addition, CNTs serve as intracellular transport carriers of immunostimulatory sequences such as CpG DNA, with enhanced cellular uptake efficiency and prolonged immunocompetence in cancer immunotherapy.^[^ ^390^ ^]^ Fullerene, a 2D allotrope of carbon, has shown significant potential in immunomodulation because of its unique chemical structure. In colorectal cancer therapy, oral fullerene tablets (OFTs) target the inflammatory microenvironment, scavenge ROS, inhibit NF‐κB and STAT3, and reduce the infiltration of proinflammatory M1 macrophages and neutrophils to restore immune homeostasis and inhibit tumor progression without significant toxic side effects.^[^ ^391^ ^]^ Curdlan‐decorated fullerene nanoparticles (Cur‐F‐NPs) alleviate immune‐mediated hepatic injury by reducing macrophage infiltration, eliminating excessive ROS, and suppressing the NF‐κB signaling pathway, which results in decreased production of proinflammatory cytokines and balanced immune homeostasis.^[^ ^392^ ^]^ Collectively, these studies underscore the versatile role of carbon‐based nanomaterials in modulating immune responses, offering new therapeutic avenues for cancer, autoimmune disorders, and inflammatory conditions.

##### 2.4.2.2 Silicon‐based Nanocomposites

Silicon is the second most abundant element in the Earth's crust and is used for a wide range of biomedical applications, as it is environmentally friendly and biocompatible.^[^ ^393^ ^]^ Silicon plays a crucial role in the synthesis of collagen and elastin, which are essential components of the aorta and other organs, such as bone and skin.^[^ ^394^ ^]^ Silica nanoparticles (SiO_2_‐NPs) possess high biocompatibility, physiochemical and surface properties with controllable particle sizes, creating avenues for surface functionalization and structural modification with enhanced biological properties.^[^ ^395^ ^]^ Despite this, their therapeutic applications are largely limited to drug delivery systems and medical diagnosis, as the poor intrinsic biodegradation of silicon biomaterials contributes to their poor efficacy in vivo and in clinical translation. To improve their efficacy, SiO_2_‐NPs are functionalized to reduce their acute toxicity. SiO_2_‐NPs containing redox nanocarriers loaded with anti‐inflammatory drugs were employed to induce anti‐inflammatory effects through ROS scavenging for the treatment of inflammatory bowel diseases.^[^ ^396^ ^]^ Vascular endothelial growth factor (VEGF)‐functionalized SiO_2_‐NPs were developed to promote angiogenesis in cardiomyocytes,^[^ ^397^ ^]^ light imaging, MRI contrast,^[^ ^398^ ^]^ radiolabeled imaging,^[^ ^399^ ^]^ ultrasound imaging,^[^ ^400^ ^]^ fluorescent magnetic imaging and photoacoustic imaging‐based applications.

Recent advancements have led to the use of silicon as 2D nanomaterials called silicene nanosheets (nSi‐NSs) with enhanced degradation properties via the wet chemical exfoliation method,^[^ ^401^ ^]^ and tailoring the desirable degradability of silicene under specific simulated physiological conditions was assessed for diagnostic imaging and phototriggered therapies.^[^ ^402^ ^]^

Compared with conventional silicon‐based biomaterials, nSi‐NSs, owing to their sp^2^/sp^3^ lattice structure, possess enhanced thermal conductivity and photothermal properties. In this context, extensive research to optimize the synthesis routes and efficacy of thin 2D silicene for photothermal therapeutic applications^[^ ^401^, ^403^, ^404^ ^]^ has been conducted. The photothermal effect of biodegradable mono‐/oligo layer silicene, with a PTCE of ∼36.09%, is elicited by inducing tumor hyperthermia followed by specific tumor ablation without significant toxicity to healthy cells, surpassing that of gold‐based nanorod nanomaterials (PTCE = ∼21%). Furthermore, silicene nanocomposites are employed as synergistic photothermal‐assisted chemotherapies, where doxorubicin‐loaded nSi‐NSs exhibit pH‐dependent drug delivery and NIR‐triggered anticancer activity.^[^ ^405^ ^]^ Additionally, compared with mesoporous SiO_2_‐NPs and porous silicon substances, nSi‐NSs have remarkable drug delivery efficacy^[^ ^406^ ^]^ and have been used as photodynamic nanosystems with tunable bandgaps and selective degradability.^[^ ^407^ ^]^ Despite the promising role of 2D silicene in oncological applications, its clinical translation is still limited by hyperthermia, indicating the need to assess the molecular mechanisms of the photonic hyperthermia of nSi‐NSs in in vitro and in vivo models.^[^ ^408^ ^]^ nSi‐NSs induce photothermia via the activation of caspase 3 and caspase 7, enhancing apoptosis in cancer cells and epigenetically suppressing the anticancer functions of proapoptotic proteins and p53. These results serve as valuable tools for future optimization and clinical translation of photothermal agents.

Ultrathin nSi‐NS surfaces modified with hydrogen atoms exhibit good stability in the TME with selective degradability in the neutral physiological environment of normal tissues. The nSi‐NSs showed good biocompatibility and hemocompatibility without inducing any inflammatory reactions, hepatotoxicity or nephrotoxicity.^[^ ^401^ ^]^ Hydrogen‐terminated nSi‐NSs, referred to as H‐silicene, are efficient at treating acute inflammation. The hydrogen atoms released from the nanosheets scavenge ROS and have anti‐inflammatory effects on inflammatory diseases.^[^ ^409^ ^]^ Recently, H‐nSi‐NSs functionalized into tricalcium phosphate scaffolds were developed for osteoimmunomodulation, where the scaffold remodeled immune cells and promoted bone repair at the site of injury. H‐nSi‐NSs were subsequently released from the scaffold, scavenged the ROS, and enabled endocytosis by macrophages, facilitating macrophage polarization to the M2 phenotype. Consequently, the immune microenvironment is converted from a proinflammatory type to an anti‐inflammatory type, leading to the degradation of the scaffold to promote osteogenesis. This study highlights the immune‐bone remodeling ability of these nanosheets, expanding their application in immunomodulation‐based regenerative medicine.^[^ ^410^ ^]^ Taking these accounts together, 2D silicon‐based nanomaterials have prospects in immunoengineered biomedical and cancer therapies.

##### 2.4.2.3 Germanium‐Based Nanosheets

Pure germanium is a lustrous gray‒white, nontoxic metalloid with chemical and physical properties similar to those of silicone and is known for its free radical scavenging, antioxidant,^[^ ^411^ ^]^ anticancer,^[^ ^412^ ^]^ immunoregulatory^[^ ^413^, ^414^ ^]^ and regenerative^[^ ^411^ ^]^ properties, making it a significant constituent of nutritional health‐protective supplements.^[^ ^415^ ^]^ Germanium can be administered as a drug or dietary supplement through the blood; after its biological functions, it is absorbed by acids and enzymes.^[^ ^416^ ^]^ A study on the underlying mechanism by which germanium plays an immunomodulatory role was reported in 130 human subjects, and it was shown that biogermanium increased natural killer cell cytotoxicity; activated immunoglobulins, B lymphocytes and TNF‐α; and regulated the NF‐κB and MAPK pathways.^[^ ^417^ ^]^ Administration of germanium in a dose‐dependent manner decreased the production of proinflammatory factors (IL‐6, IL‐1β and TNF‐α) and increased IL‐10 in microorganism‐induced mastitis, indicating its potential for treating inflammatory diseases.^[^ ^418^ ^]^ Knee sleeves embedded with germanium were developed to treat knee osteoarthritis, as germanium‐free electrons undergo transdermal effects, creating a microelectromagnetic field in the embedded fabric, which consequently increases blood circulation and tames the inflammatory process.^[^ ^419^ ^]^

The recent development of a 2D germanium allotrope similar to graphene called germanene has been reported,^[^ ^420^ ^]^ and these monoelemental germanenes with unique structural and optoelectronic properties have received significant attention for biomedical applications.^[^ ^362^ ^]^ Rapidly biodegradable and photothermal free‐standing 2D germanene nanosheets (nGe‐NSs) have provided high photoacoustic contrast, with a PTCE of ∼38% in NIR‐II biowindow in vivo cancer models.^[^ ^421^ ^]^ Briefly, the obtained nGe‐NSs Doxorubicin‐functionalized nGe‐NSs increased drug accumulation in drug‐resistant cancer cells and exhibited a maximal drug effect of 62.8%, with no significant cytotoxicity or hemolysis.^[^ ^422^ ^]^ Conjugation of the chemotherapeutic drug doxorubicin to nGe‐NSs Elsewhere, owing to the sensitivity of germanene to ultrasound and hyperthermia, these nanosheets aided in photothermal conversion and photoacoustic imaging with enhanced biocompatibility and oxidative biodegradability. Here, the ability of germanene as a multifunctional cancer therapeutic was explored because it is easily degraded after it scavenges tumor cells.^[^ ^421^ ^]^ Germanium's antioxidant properties aid in the ability of hydrogen‐terminated germanene nanosheets to scavenge ROS in acute kidney injury. These nanosheets accumulate in injured kidneys and exert robust antioxidant protective effects by regulating inflammation through free radical and redox reaction absorption.^[^ ^423^ ^]^ In summary, germanium and 2D germanium are powerful agents for immunomodulation, cancer therapeutics and the treatment of anti‐inflammatory diseases.

##### 2.4.2.4 Tin‐Based Nanocomposites

Tin, a pliable, soft metallic element, is highly corrosion resistant and is used as a protective coating for other metals for electrical circuits. However, recent research by Ahmad et al. revealed that tin nanoparticles (Sn‐NPs) have antioxidant, hematoprotective and cytotoxic potential^[^ ^424^ ^]^ in hemolytic anemia. Sn‐NPs play an immunomodulatory role by decreasing the levels of proinflammatory cytokines and increasing the levels of anti‐inflammatory cytokines; play a hematoprotective role by increasing the blood cell counts of total platelets, white blood cells, and granulocytes; and have antioxidant potential by increasing the levels of GPX, CAT and SOD. Furthermore, Sn‐NPs decreased the serum erythropoietin, ferritin and ferrous iron levels, which is characteristic of their potential for treating hemolytic anemia. Functionalized Sn‐NPs are employed in biosensing applications. Cerium oxide/tin oxide nanocatalysts (CeO_2_/SnO_2_‐NPs) can be used to sense anti‐inflammatory drugs electrochemically, and the combination of two metal nanoparticles improved their physiochemical and electrocatalytic properties, facilitating efficient sensing of the drug mesalamine.^[^ ^425^ ^]^ The anti‐inflammatory properties of SnO_2_‐NPs loaded on a chitosan‒polyethylene glycol complex can be improved by modulating the levels of cytokines and their antioxidant properties by increasing their antioxidant enzyme activities, preventing lipid peroxidation and oxidative stress, and increasing their antiarthritic potential by reducing bone depletion in the immunomodulatory treatment of rheumatoid arthritis.^[^ ^426^ ^]^

Despite the promising biomedical applications of Sn‐NPs, their potential toxicity should be considered, as indium‐tin oxide (ITO) is employed as a transparent conductive coating in the electronics industry, and the production of ITO releases toxic pollutants and indium‐related compounds, causing indium‐induced interstitial lung disease called alveolar proteinosis. In this context, ITO nanoparticles were synthesized and administered intraperitoneally to mice to determine the occupational exposure effects of indium. ITO activated NOD‐like receptor pyrin domain‐containing 3 (NLRP3) in macrophages, followed by neutrophil recruitment and IL‐1β production. Macrophage endocytosis of ITO induced the production of TNF‐α, cell death and the activation of T helper cells, which are characteristic of pyroptosis. This study also cocultured macrophages impaired by ITO with mesenchymal stem cells (MSCs), suggesting that MSCs are effective therapeutics for indium‐induced pulmonary alveolar proteinosis.^[^ ^427^ ^]^ Liu et al. also investigated the toxicity induced by ITO and observed oxidative stress, chronic pulmonary inflammation, and lung fibrosis through the release of Il‐1β, IL‐6 and TNF‐α; the number of alveolar macrophages; and the NF‐κB pathway, which affects both the morphology and function of pulmonary organs.^[^ ^428^, ^429^ ^]^

Stanene is a recently synthesized 2D tin‐based monoelemental material akin to graphene, with unique physicochemical properties, enhanced thermoelectric performance, optoelectronic properties and topological superconductivity.^[^ ^430^ ^]^ Biomedical applications of stanene nanosystems have been investigated because of their good stability and photonic and sonodynamic properties. The potential effect of stanene‐based nanosheets for the delivery of anticancer β‐Elemene drugs and ultrasound‐assisted combination cancer therapy was reported, where the nanosheets presented an enhanced PTCE of ∼40% and acted as a nanocarrier due to their inherent 2D‐planar structure.^[^ ^431^ ^]^ This nanosystem acts as a promising sonosensitizer to achieve ultrasound‐triggered ROS generation for application in sonodynamic therapy. Owing to the excellent ability of stanene to generate reactive oxygen species (ROS) under ultrasonic stimulation to damage bacterial structures, a stanene‐loaded hydrogel system was developed to test its sonodynamic antibacterial effect. This antibacterial effect is harnessed to promote wound healing in the treatment of drug‐resistant infections, and the hydrogel reduces inflammation at the wound site.^[^ ^432^ ^]^ PEGylated stanene nanosheets with increased stability and biocompatibility were prepared to test their multifunctional role in cancer therapy. The release of free electrons from the upper and lower stanene layers and their ability to convert light to heat at the tumor site assist in photothermal cancer elimination and potential use as multimodal imaging‐guided therapy.^[^ ^433^ ^]^ Drug‐loaded stanene nanosheets tend to repolarize macrophages associated with tumors and hence are effective in chemoimmunotherapy. Immunosuppression in the TME by tumor‐associated macrophages (TAMs) reduces the efficiency of immunotherapy, and drug‐loaded drugs overcome TAM‐induced immunosuppression by polarizing M2‐tumor support, such as TAMs, to the M1‐tumor suppressive phenotype. They also enhance CD4+ and CD8+ T lymphocytes, mature dendritic cells and deliver chemotherapeutic drugs, amplifying antitumor effects. This novel study highlights the immunomodulatory potential of stanine in improving cancer therapy to develop nanoimmunotherapeutics.^[^ ^434^ ^]^

Thus, group 14 elements, with graphene as their forerunner, Si, Ge and Sn, have demonstrated extraordinary potential as 2D monoelemental nanoforms such as silicene, germanene and stanene. These “ene”‐ending 2D nanomaterials exhibit unique optoelectronic and photothermal properties, modulate immune responses, scavenge ROS and promote tissue regeneration, making them versatile candidates for next‐generation therapeutics and regenerative medicine.

#### 2.4.3 Group‐15 Elements and Reported Immunoengineering Application

Group 15 consist of nitrogen (N), phosphorus (P), arsenic (As), antimony (Sb), bismuth (Bi), and highly radioactive moscovium (Mc). As one of the most abundant elements, nitrogen plays a significant role in various biological mechanisms and exhibits indirect effects on immunomodulatory functions. Nitrogen readily reacts with different elements to form different 2D hybrid, hetero, and composite nanostructures with enhanced physicochemical and biological properties. The immunomodulatory applications of 2D P, As, Sb and Bi are discussed below.

##### 2.4.3.1 Phosphorus Based Nanocomposites

Phosphorous, an abundant multivalent non‐metal plays a vital role in sustaining life on earth by maintaining the biosphere, hydrosphere and geosphere. It plays a dominant role in energy metabolism of carbohydrates and fats, regulates the cell growth and repair of tissues. Phosphorous‐based nanomaterials range from biodegradable black phosphorus nanosheets to phosphorous containing dendrimers and metal phosphates, are employed in biomedical applications due to their unique physiochemical, optical, and biological properties. They are employed as biosensors, drug carriers, cancer theranostics as tumor imaging agents, photothermal/dynamic agents and tissue regenerators, especially in bone regeneration.^[^ ^435^ ^]^ Black phosphorous nanosheets (BP‐NSs) biodegrade to phosphate ions that affects ATP hydrolysis^[^ ^436^ ^]^ leading to tumor cell death,^[^ ^437^ ^]^ by selectively inducing high intracellular oxidative stress in cancer cells while remaining biocompatible with normal cells. BP targets the aggregation of β‐amyloid peptides (characteristic feature of neurodegenerative Alzheimer's disease), inhibits ROS generation, targets neuroinflammation and stabilizes the calcium homeostasis.^[^ ^438^ ^]^ Due to the low toxicity, high stability, and metal free high PTCE of native BP and its derived planar form, they are highly desirable photo‐thermal agents when compared to conventional carbon nanostructures. Biocompatible BP nanosheets integrated with cellulose elicits its anti‐cancer effects due to its high PTCE, exhibiting strong absorbance in the NIR regions, demonstrating high tumor elimination efficiency.^[^ ^439^ ^]^ Ultrathin BP‐NSs with high PTCE act as photothermal agent and immunological stimulator relieving immunosuppression in TME. by recruiting monocytes in initiating a tumor specific immune response.^[^ ^440^ ^]^ BP‐NSs’ PTCE can reach up to 31%; when combined with immunotherapy; they inhibit the tumor growth and prevent tumor recurrence. This is achieved by stimulating host immune system to develop antitumor immunity to protect normal cells during cancer treatments.^[^ ^441^ ^]^ Carbon dot‐passivated BP nanosheets (CD‐BP‐NSs) possess significantly higher PTCE of 77.3% in NIR‐I biowindow and 61.4% in NIR‐II biowindow, exceeding that of pristine BP's PTCE of 50% at NIR‐I and 28% at NIR‐II biowindows.^[^ ^442^ ^]^ BP_NSs excel as drug delivery agents with high drug loading efficiency, which is helpful in administering tumor‐suppressing drugs through these photothermal agents.

Monoelemental 2D layered form of phosphorus named “phosphorene” possess outstanding optoelectronic properties.^[^ ^443^ ^]^ Phosphorene (nP) is a 2D Xene nanomaterial akin to graphene with unique anisotropic orthorhombic structure, physical, optical, and biological properties. nP shows promise in caner treatment and serves as template for anti‐cancer drug loading and release mechanisms, without causing any significant change in chemical structure of the drug.^[^ ^444^ ^]^ Owing to the biocompatible, high adsorption photoredox capability, intrinsic fluorescence and photoacoustic (FLPA) properties, prevention of PTT‐triggered hyperthermia induced inflammatory diseases and selective ROS generation in TME, maximizes therapeutic efficacy and minimizes systemic toxicity of nP. Thus, phosphorene‐based technology is viable for multifunctional biomedical applications including cancer therapy, treatment of degenerative disorders, stem‐cell therapy, blood‐brain barrier, and targeted delivery applications.^[^ ^445^ ^]^

Phosphorus containing dendrimers play therapeutic role by breaking down into non‐toxic substances in the treatment of autoimmune inflammatory disease, rheumatoid arthritis (RA).^[^ ^446^ ^]^ The multivalent nature of these dendrimers interact with multiple cellular targets; specifically targeting monocytes to decrease pro‐inflammatory cytokines and increase anti‐inflammatory cytokines’ characteristic to T helper 2 response. These dendrimers extend its therapeutic role by inhibiting the signalling pathway responsible for monocyte differentiation into osteoclasts, in RA.^[^ ^447^ ^]^ Water‐soluble, biodegradable phosphorous containing dendrimers hold high potential as nanocarriers to delivery chemotherapeutic drugs like paclitaxel^[^ ^448^ ^]^ and doxorubicin. The, nanomaterials are endosomally taken up in TME to sustainably release high concentrations of the chemotherapeutic drug.^[^ ^449^ ^]^

Metal‐based phosphate nanomaterials, like calcium phosphates and hydroxyapatite, mimic natural inorganic bone constituents and hence used in bone tissue engineering.^[^ ^450^ ^]^ Calcium phosphate‐based nanocomposites with increased functionalization are employed as effective diagnostic, cellular (nucleic acid drugs) delivery agents and nanocarriers, replacing conventional synthetic polycationic nanocarriers.^[^ ^451^ ^]^ Calcium phosphate nanoparticles (CaP‐NPs) improve heart function and can treat heart failure, when loaded with drugs and injected. Inhaled peptide loaded CaP‐NPs reached the heart faster, were taken up by the cardiomyocytes and improved cardiac function in diabetic cardiomyopathy animal models. showing their potential use as invasive targeted cardiac drug delivery method in humans. The nanoparticles protect the loaded peptides from immediate enzymatic degradation, providing cellular permeability into the cardiomyocytes and release bioactive microRNA, improving cardiac function without inducing any oxidative stress, toxicity or interfering heart function TME is characterised by low pH, high ROS, high GSH and overproduced hydrogen peroxide. To effectively target the tumor, the physiological behaviour of nanoparticles at low pH conditions needs validation. Hollow Manganese phosphate nanoparticles were employed as multifunctional probes for synergistic cancer therapy^[^ ^452^ ^]^ and covalently functionalized with poly (ethylene glycol) with chemotherapeutic drug. The nanoparticles possessed low MRI at pH 7.4, but exhibited high MRI contract at pH 5.4; leading to the release ofMn^2+^ upon erosion, facilitating targeted delivery of chemotherapeutic drug DOX. This pH‐controlled MRI and pH‐triggered drug delivery are ideal for diagnosis and treatment of cancer. Copper phosphide nanowires serves as electrocatalytic cancer cell detection probe to facilitate selective and sensitive detection of hydrogen peroxide (H_2_O_2_), crucial indicator of physiological cellular homeostasis.^[^ ^453^ ^]^ Tumor cells release H_2_O_2_, when detected under ROS conditions abnormal H_2_O_2_ levels indicate cell aberrance, imbalance ROS and apoptosis, all of which are relate to disorders like Parkinson's disease, myocardial infraction, and cancer. Hence, H_2_O_2_ sensing is important and metal‐based phosphide nanomaterials act as non‐enzymatic sensors in this application.

These aforementioned properties of phosphorus nanomaterial, help in its application in cancer detection/treatment/imaging, antioxidant potential, H_2_O_2_ sensing, ROS reduction, photoredox potential and immunomodulatory regenerative medicine.

##### 2.4.3.2 Arsenic Based Nanocomposites

Chronic environmental arsenic exposure leads to inflammatory infiltration, renal edema resulting in renal failure and liver inflammation, by activating pro‐inflammatory signalling pathways, ROS induction and consequent, DNA damage, impairs both innate and adaptive immune system.^[^ ^454^ ^]^ Arsenic alters proliferation, differentiation, and function of T lymphocytes^[^ ^455^ ^]^ making it a promising therapeutic for lymphoproliferative autoimmune syndromes that damage lungs, skin and kidneys.^[^ ^456^ ^]^ Arsenic extends a pro‐oxidant effect and increases the ROS levels in macrophages, lymphocytes, and several cell types. Arsenic aids in the management of chronic graft vs host disease, by the depletion of glutathione and ROS production,^[^ ^457^ ^]^ prolonged allograft survival by repressing CD4+^[^ ^458^, ^459^ ^]^ and CD8+ memory T cells and redox‐sensitive signalling pathways modulation by DNA damage, immune cell gene expression impairment,^[^ ^460^ ^]^ cell stress‐induced NRF2 regulation of antioxidant genes and IL‐12 inhibition in dendritic cells.^[^ ^461^ ^]^ Studies show that arsenic targets nucleotide‐binding oligomerization domain‐like receptor proteins (NLRP) inflammasome complexes, blocks its activity, and affects IL‐1β showing its application in treating chronic inflammatory responses in inflammasome ‐mediated diseases.^[^ ^462^ ^]^

Owing to the pro‐oxidant and ROS generating ability of arsenic, layered 2D structure of arsenic called arsenene (nAs), are widely employed in cancer therapies including its application as enzymatic phenol biosensor and as therapeutic agents for acute promyelocytic leukemia cells (APL).^[^ ^463^ ^]^ Planar nAs and arsenic nanomaterials (As‐NMs) obtained by wet chemical exfoliation method have shown promising therapeutic anticancer effect against APL cells through reducing super‐oxide dismutase activity and generating ROS mechanism.^[^ ^464^, ^465^, ^466^ ^]^ Furthermore, the cytotoxicity of 2D arsenene nanosheets (nAs‐NSs) with different types of cancer cell lines, including A549, A2780, HeLa, and MCF‐7 was assessed and compared to normal human cell lines (HL‐7702 liver and HEK293 embryonic kidney). nAs‐NSs demonstrated effective therapeutic anticancer properties with 50–80% inhibition affecting nuclear deoxyribonucleic acid (DNA) and subsequent nuclear protein TXNL1 in treated‐NB4 cancer cells to effectively suppress their proliferation and induce pyrimidine metabolism pathways. Development of a therapeutic nanomotor consisting of nAs‐NSs for anticancer drug delivery, with intrinsic cytotoxicity, motion capabilities, and drug carrier competency of the immunoengineered arsenene‐based system was investigated.^[^ ^467^ ^]^ nAs‐NSs are highly biocompatible and enhanced cellular uptake, and targeted delivery for future self‐carrier therapeutic approaches using arsenic nanocompounds.

##### 2.4.3.3 Antimony Based Nanosheets

Antimony is a durable, semi‐metallic element that naturally occurs in environment and its commonly used to develop frame‐ retardant materials, pottery, alloys for machine bearings and bullets. Chronic exposure to antimony and its compounds leads to respiratory, cardiovascular, gastrointestinal and lung inflammation, and elicits human carcinogenicity. However, since the 16th century, antimony‐based compounds have been used in medicine as tartar emetics for treatment of inflammatory conditions and diseases like Leishmania.^[^ ^468^ ^]^ Anti‐inflammatory antimony compounds have also been used as antiprotozoal drugs to treat psoriasis, a chronic inflammatory disease with characteristic hyperproliferation of skin keratinocytes. In psoriasis conditions, cytokines are activated by the activation of the NF‐κB, STAT3 pathways, type 1 interferon (IFN) signaling pathways and antimony compounds influenced the production of pro‐inflammatory cytokines IL‐6 and IL‐8, and reduced the dendritic cell activation and IL‐23 expression respectively.^[^ ^469^ ^]^ Studying the toxicity profile of antimony oxide nanoparticles (Sb_2_O_3_‐NPs) in comparison to 7 other metal oxide nanoparticles on different hematopoietic human cell lines, showed that Sb_2_O_3_‐NPs extended specific toxicity to erythroid precursor proliferation and inhibited the differentiation of T helper 1 monocytes to macrophages.^[^ ^470^ ^]^ Sb_2_O_3_‐NPs showed low degree of aggregation when in contact with cells, as the nanoparticles interacted with the erythroid precursors at the plasma membrane level. Sb_2_O_3_‐NPs possess photocatalytic activity due to its optoelectronic and photoelectric properties and when incorporated into poly (o‐phenylenediamine), this nanocomposite aided in the photocatalytic degradation of Ibuprofen, a non‐steroidal anti‐inflammatory drug and organic pharmaceutical pollutants from the environment.^[^ ^471^ ^]^ Antimony selenide nanosheets conjugated with polyvinyl pyrrolidone (PVP) and irradiated by infrared laser efficiently act as antibacterial agents to combat multi‐drug resistant bacteria via ROS induction.^[^ ^472^ ^]^ PVP modification enhanced nanosheets’ bioinertness and the nanosheets’ photothermal‐induced antibacterial property aided in complete eradication of bacterial infections.

2D elemental layered nanostructures of antimony called antimonene are gaining attentions for biomedical and therapeutic applications.^[^ ^467^ ^]^ Zhang et al reported the fabrication of poly(lactic‐co‐glycolic acid)‐coated antimonene nanosheets as an X‐ray radiosensitizer.^[^ ^473^ ^]^ Upon X‐ray irradiation, the nanosheets induced strong oxidative stress by the increased generation of ROS to effectively accelerate electron transfer by radiocatalysis and inactivate vascular endothelial growth factor‐regulated hypoxia. Notably, without radiosensitization, the antimonene‐based nanosystem exhibited significant cytotoxicity with the co‐cultured cancer cell lines and upon radiosensitization induced cancer cell apoptosis. This shows the importance of tunable antimonene to be used in future radiosensitization based clinical therapeutics. Dual‐responsive 2D antimonene nanosheets (nSb‐NSs)‐based drug‐loading system for cancer theranostics was reported.^[^ ^474^ ^]^ nSb‐NSs are constructed by wet chemical exfoliation of bulky antimony and surface modified with PEGylation and doxorubicin loading due to hydrophobic interactions and this nanoplatform can be activated by both internal stimuli of acidic pH stimuli and external stimuli of NIR light at 808 nm. With drug‐loading capacity of 150% and PTCE of 42%, this nanoplatform was readily taken up through endocytosis and enhanced tumor growth inhibition by NIR irradiation. Likewise, application of a 2D antimonene‐based heterostructure with enhanced capability of ROS generation was reported for photonic cancer theranostics.^[^ ^475^ ^]^ Ultrathin 2D antinomy‐based sheets were functionalized with a photosensitizer and modifier, with an average thickness and particle size of 4 nm and 110 nm, respectively, are fabricated. Their structural arrangement facilitated a loading capacity as high as 83% for 5,10,15,20‐Tetrakis(4‐hydroxy‐phenyl)‐21H,12H‐porphine as photosensitizer, PTCE of ∼ 44.6% and improved electron‐hole pairs to hold redox potential for ROS generation at tumor site. Further, multimodal imaging properties of this nanosystem were owed to its photothermal and photoacoustic imaging ability, paving the way toward future image guided therapeutics of 2D antimony‐based nanomaterials.

##### 2.4.3.4 Bismuth Based Nanosheets

Bismuth, brittle and crystalline pinkish‐white metallic element is the heaviest and “green” metal due to its low toxicity (much lower than sodium chloride) and benign nature.^[^ ^476^ ^]^ Bismuth finds its application in manufacturing industry and cosmetics. Though bismuth has no known biological function, the low‐toxicity and tunable nature of bismuth compounds contributes to its use in biological applications. Compared to arsenic and antimony, large concentration of bismuth can be tolerated by the human body. Bismuth‐based drugs are commercially used for the treatment medical disorders and used for their antimicrobial and anticancer properties. Bismuth complexes strongly bind to proteins, glycoproteins, and enzymes of the connective tissue, and inhibits microbial adherence, eliciting a synergistic effect by modulation of metal‐associated toxicity and enhancement of biological interactions.^[^ ^477^, ^478^ ^]^ Bismuth nanoparticles (Bi‐NPs) are used for treating multidrug resistant bacteria in combination with x‐ray‐based therapies, where upon X‐ray irradiation, tBi‐NPs exhibit photothermal effect. They directly interact with bacterial cell wall, leading to ROS generation, limiting biofilm formation and stimulating immune reaction. This same phenomemon is observed in bismuth bisulphide nanoparticles.^[^ ^479^ ^]^ Bismuth nanoparticles (Bi‐NPs) also help in curing infections resulting from inflammatory diseases, by attacking the pathogen causing inflammation^[^ ^480^ ^]^ and can be amalgamated with antibiotics for increase sensitivity to microbes.^[^ ^481^ ^]^ Bi‐NPs synergistically aid in antibiotics delivery^[^ ^482^ ^]^ and photothermally aids in breaking the bacterial wall making antibiotics accessible.

2D bismuthene nanosheets (nBi‐NSs) with desirable microstructure and biological properties are explored in cancer theranostics.^[^ ^483^, ^484^ ^]^ Biocompatible nBi‐NSs engineered as biomimetic radiosensitizer enhanced the radiation‐killing of cancer cells without disturbing the normal cells around the tumor site and modulate radiotherapy‐resistant hypoxic tumor by inducing excess ROS. Functionalised nBi‐NSs with quantum dots and chemotherapeutic drugs elicited pH ‐sensitive and photothermal induced drug release in the TME. This shows the photodynamic properties of bismuth to induce oxygen radicals, PTCE of ∼54% and the chemotherapeutic drug loading ability of ∼250%, can be used as extraordinary cancer therapeutics. Gou et al, utilized the pH sensitive and photothermal property of bismuthene in combination with cancer immunotherapy in sequentially triggering programmed death‐ligand 1 siRNA release. nBi‐NSs simultaneously induce the pathological permeability, retention and rapid clearance leading to inhibition of both local and distant metastasis, by enhancing DC maturation and immunosuppressive tumor reversion.^[^ ^485^ ^]^ Further, photothermal nBi‐NSs are employed as potential drug carriers and can reduce carbon dioxide into carbon monoxide (CO) by adapting a photocatalytic route. to reduce the chemotherapeutic drug resistance and photothermal‐induced inflammation.^[^ ^486^ ^]^ These immunoengineered bismuthene‐doxorubicin nanosystems improved tumor inhibition up to 50% and upon simultaneous irradiation with IR and NIR, the tumor inhibition rate increased to 90%. in the tested tumor‐bearing mouse models. These findings further support the multifunctional immunomodulatory anticancer properties of bismuthene‐based system in future clinical therapies.

The extensive research discussed above on 2D forms of group 15 elements, including P, As, Sb and Bi, exhibits their remarkable pH‐sensitive properties aligning with increasing metallic character and decreasing electronegativity down the group. These characteristics enhance their potential in biomedical applications alongside their pH/photothermal‐triggered chemotherapeutic drug release, antimicrobial, immunomodulatory and biosensing properties for future thernaostics.

#### 2.4.4 Group‐16 Elements and Reported Immunoengineering Application

Group 16, the oxygen‐group elements, also called chalcogens consist of oxygen (O), sulfur (S), selenium (Se), tellurium (Te), polonium (Po), and livermorium (Lv). Owing to a biradical electron configuration, oxygen is highly reactive, forming compounds with other elements rather than its own stable 2D structures, and its poor intrinsic properties limits the synthesis of stable 2D nanomaterials. Polonium is rare, highly toxic and radioactive with no biological role. Recently, studies have explored the synthesis of stable layered 2D polonium nanosheets (poloniumene)^[^ ^487^ ^]^ Similar to molybdenum disulfide, these nanostructures tend to form three‐atomic‐layers with desirable large band gap and stable monolayers.^[^ ^488^ ^]^ However, no significant biomedical applications have been reported; instead, research focuses on assessing risks of radioactive polonium exposure from various sources. Livermorium, discovered in 2000 by bombarding curium with calcium, is extremely radioactive, has no biological role and is used in research to study the behaviour of superheavy atoms. The biomedical applications of the other elements of this group are discussed below.

##### 2.4.4.1 Sulfur Based Nanocomposites

Sulfur plays a vital role in various biological pathways and has been used in dermatology for centuries due to its biologically active nature. Nipin et al, validated sulfur's anti‐inflammatory effects in modulating LPS‐induced skin inflammation, where sulfur suppressed the ROS and DNA damage in skin fibroblasts.^[^ ^489^ ^]^ This study shows potential of sulfur in developing safe cosmetics. Sulfur‐based nanoparticles (S‐NPs) exhibit anti‐bacterial properties and are employed in treating dermatological diseases, effectively disrupting the bacterial cell wall of Staphylococcus bacteria that causes acne vulgaris.^[^ ^490^ ^]^ Synthesis of anti‐fungal and anti‐microbial S‐NPs from citrus limon leaves was validated as synergistic, safe nanohydrogel formulation for clinical use.^[^ ^491^ ^]^ These studies validate the anti‐bacterial, anti‐fungal and anti‐microbial role of S‐NPs in synergistically treating skin inflammation and developing safe dermal applications.

Elsewhere, selenium‐sulfur nanocomposite showed accelerated tissue regeneration by enhancing angiogenesis and regulating inflammation.^[^ ^492^ ^]^ Transcriptomic analysis showed that nSeS promoted biosynthesis and reduced inflammation by targeting ROS in zebrafish and mice models. During the early stage of tissue regeneration, nSeS improved the accumulation of inflammatory cells, increased CD206+ macrophages and decreased myelopeoxidase‐positive neutrophils. This study proved that the incorporation of sulfur was the driving force to accelerate tissue regeneration and reduce inflammation. S‐NPs extend their synergistic anti‐microbial and immunomodulatory activities in potential wound healing applications.^[^ ^493^ ^]^ In addition to this, synthetic 2D planar sulfur‐based materials possess TME responsive, anticancer properties. Li et al, studied this by incorporating tirapazamine and indocyanine green into sulfur nanosheets with the loading efficiency of around 6% and 95%, respectively.^[^ ^494^ ^]^ Compared to carbon nanomaterials, sulfur nanomaterials have unmatched antibacterial, antifungal activity and possess large specific area, enhanced dispersibility and optical transparency, making them excellent candidatures for multifunctional photothermal/dynamic/chemotherapy. On the onset of high H_2_O_2_ in TME these sulfur nanomaterials decompose, to elicit its photothermal‐assisted chemotherapeutic effect., These nanocomposites demonstrate suitable biocompatibility alongside NIR fluorescence, ROS generation, and PTCE for enhanced TME responsiveness in vitro and inhibitory effect on tumor growth in vivo. Also, design and application of hydrothermally synthesized layered sulfurene nanosheets by assembling sulfur quantum dots was tested for visible‐light‐active photocatalysis and optical sensing applications.^[^ ^495^ ^]^ This beneficial nanomaterial‐based strategy for offered diagnostic and therapeutic approaches, owing to its optical absorption and multi‐wavelength photoluminescence and photoelectric properties.

Overall, sulfur and its 2D nanocomposites elicit extraordinary anti‐microbial, anti‐inflammatory, tissue regenerative, photothermal assisted anti‐cancer and immunomodulatory effects.

##### 2.4.4.2 Selenium Based Nanocomposites

Selenium's physicochemical properties are similar to sulfur and tellurium, and is typically found un metal sulfide ores. The World health organization (WHO) recommends a daily dietary consumption of 40µg of selenium due to its nutritional benefits^[^ ^496^ ^]^ in regulating various homeostatic mechanisms and normalizing cell proliferation.^[^ ^497^ ^]^ Selenium nanoparticles (Se‐NPs) are widely used for their antioxidant, anticancer, antimicrobial, and immunomodulatory roles in various biomedical applications. Se‐NPs extends its antioxidant property by scavenging ROS and modulating oxidative stress, major factor in disease progression. They increase biodegradability of nutritional supplements aiding their clearance from the body.^[^ ^498^ ^]^ In cancer therapy, Se‐NPs are encapsulated by immune cells, causing mitochondrial ROS induction, ATP depletion and mitochondrial damage, ultimately resulting in tumor ablation.^[^ ^499^ ^]^ Thus, Se‐NPs are excellent candidates for cancer therapy due to its prophylactic ability to selectively target tumor cells without inducing host toxicity and can be to exhibit enhanced anti‐cancer effects.^[^ ^500^, ^501^, ^502^, ^503^ ^]^

Selenium deficiency leads to several diseases like atherosclerosis, spinal cord injury, rheumatoid arthritis, diabetes, epilepsy, renal diseases by promoting inflammation and reactive oxygen species (ROS) generation. Selenoprotiens boost the immune system by exhibiting antioxidant functions. They influence effector functions of immune cells by maintaining redox, oxidative stress burst, protein folding and regulating immunity.^[^ ^504^ ^]^ These selenoproteins exhibit antioxidants and anti‐inflammatory effects in wound healing,^[^ ^505^ ^]^ reducing ROS, lowering fatty acid and lipid peroxides levels,^[^ ^506^ ^]^ translocating NF‐κB,^[^ ^507^ ^]^ affecting lymphocyte proliferation^[^ ^508^ ^]^ and regulates the monocyte adherence.^[^ ^509^ ^]^ Se‐NPs’ immunostimulatory effects were first reported in breast cancer model.^[^ ^510^ ^]^ Se‐NPs’ induced delayed type hypersensitivity by increasing natural killer (NK cells, INF‐γ and IL‐17. Further, Se‐NPs exerts immunomodulatory role by increasing IL‐8, IL‐6 and TNF‐α in colon cancer treatment;^[^ ^511^, ^512^ ^]^ TNF‐α, interferon regulatory factor (IRF1) and receptor interacting protein (RIP1) in prostate cancer treatment^[^ ^513^ ^]^ and, increasing IL‐33, IL‐1β, IL‐6, superoxide dismutase (SOD) and decreasing glutathione (GSH), glutathione peroxidase (GPx) in treatment of hepatocellular cancer.^[^ ^514^, ^515^ ^]^ Further, Se‐NPs extend anti‐inflammatory ability to reduce the inflammation by reducing leukocyte count, TNF‐α, prostaglandin E_2_ (PGE_2_), nitric oxide (NO_x_) and lipid peroxidation.^[^ ^516^ ^]^ Se‐NPs are employed in treating inflammatory diseases such as chronic inflammatory rheumatoid arthritis^[^ ^517^, ^518^ ^]^ and nephropathy.^[^ ^519^ ^]^ In immunomodulated treatment of rheumatoid arthritis, Se‐NPs increased GSH, CAT, SOD, GPX proteins and, decreased COX‐2, IL‐1β, IL‐6 and MCP‐1. In atherosclerosis, Se‐NPs decreased lipid peroxidation and increased NO, GPX, SOD and catalase (CAT) levels.^[^ ^520^ ^]^ Se‐NPs increased the M2 macrophage, SOD, GSH, GPX and decreased TNF‐α, Il‐6, ROS to treat spinal cord injury.^[^ ^521^ ^]^ In epilepsy, Se‐NPs increased GSH, GPX, SOD, CAT, nuclear factor‐erythroid factor 2‐related factor 2 (Nrf2), heme oxygenase (HO‐1) and decreased IL‐1β, TNF‐α, COX‐2, NF‐κB levels.^[^ ^522^, ^523^ ^]^ Se‐NPs functionalized with mannose‐rich oligosaccharides (MRO) were developed to treat inflammatory bowel disease, like ulcerative colitis, by improving M2 phenotype polarization, reducing IL‐1β, IL‐6, IL‐12, monocyte chemoattractant protein (MCP‐1) and TNF‐α cytokines. showing the resolution of inflammation in this disease model. Amit et al, showed that selenium deficiency was observed in COVID‐19 patient and supplementing the diet with Se‐NPs can overcome this deficiency that leads to increased mortality. Se‐NPs boost selenoprotien activity and reduce secondary bacterial infections associated with the COVID‐19.

In addition to the therapeutic applications of Se‐NPs, development of 2D layered nanostructures of selenium (selenene) has been theoretically reported.^[^ ^524^ ^]^ Owing to the chair‐like buckled structural similarity with germanene and siliciene, 2D selenene and similar to group IV Xenes, it the Se atoms are held by covalent bonds in its layered structure.^[^ ^525^ ^]^ 2D selenene are potential anti‐metastatic/anti‐angiogenic/ nanoagent^[^ ^362^ ^]^ and effective radiosensitizer for cancer radiotherapy applications. Their favorable physical, electronic and optoelectronic properties of 2D selenene and ultrathin can be used in combinational cancer therapy.^[^ ^525^ ^]^ 2D ultrathin selenium‐based nanosheets with tunable optical and optoelectronic properties were synthesized using a typical wet exfoliation method^[^ ^526^ ^]^ exhibited photoluminescence and ultrafast photonics. Overall, 2D selenium offer a wide range of favorable diagnostic and therapeutic effects in various inflammatory disease models, suggesting in‐depth preclinical investigations for its safe use and commercialization in future therapeutics.

##### 2.4.4.3 Tellurium Based Nanocomposites

Tellurium is a rare, toxic, teratogenic metalloid with limited biological mechanisms. However, advancements in nanotechnology led to the experimental construction of stable 2D tellurium‐based nanosheets, reported in 2018. 2D forms of tellurium include, tullurene (nTe), the 2D monoelemental material Xene, transition metal tellurides (TMTs), optoelectronic tellurium nanosheets, antioxidant tellurium nanorods, tellurium nanoparticles and photoconductive nanowires. Zhongjian et al, prepared 2D structure of crystalline tellurium layers with covalent Te‒Te bonds in their intrachain and Van der Waals forces in their interchains^[^ ^527^ ^]^ offering desirable photoresponse and photoelectrochemical properties under simulated lights at different wavelengths (350 to 475 nm). Thereby, biomedical applications of 2D nTe have received attention due its potential electronic and optoelectronic properties. Ultrathin tellurene nanosheets modified with polyethylene glycol (nTeNS‐PEG) were fabricated for combined cancer thermo‐chemotherapy.^[^ ^528^ ^]^ nTeNS‐PEG's large‐surface‐area efficiently generated local hyperthermia with a high drug loading capacity (around 160%) and significant PTCE of ∼55% under laser irradiation at 808 nm. These nanosheets induce TME‐triggered drug release, showing the combined effect of the chemotherapeutic drug and photothermal nanosheets in chemo/photothermal cancer nanomedicine. Furthermore, the use of biocompatible 2D tellurium‐based sheets was suggested for PAI‐guided photodynamic therapy^[^ ^529^ ^]^ by generating ROS under light irradiation of around 670 nm for multispectral optoacoustic tomography. nTe nanosheets are efficient plasmonic biosensors and reported to significantly detect SARS‐CoV‐2 virus.^[^ ^530^, ^531^ ^]^

Tellurium nanoparticles (Te‐NPs) exhibit antioxidant, antimicrobial, cytotoxic and photothermal properties.^[^ ^532^ ^]^ Furthermore, numerous selenium and tellurium nano‐derivatives are used in research for their anti‐proliferative and antioxidant roles to serve as chemotherapeutic agents.^[^ ^533^, ^534^, ^535^ ^]^ Te‐NPs enhance the photothermal properties of selenium and together, they, generate ROS, mimic pro‐oxidant roles, boost antioxidant mechanisms, induce apoptosis and stimulate the immune response in inflammation. In detail, activated by photothermally, radiation or chemical stimulation in the tumor site, these nanocomposites inhibit metastatic tumor cell proliferation in local and distant sites by the stimulation of immune response and exhibiting selective toxicity to cancer cells, and prevents the formation of malignant tissues.^[^ ^533^ ^]^ Immunomodulatory selenium‐tellurium derivatives are used in tropical formulations to treat dermatitis, by effectively inhibiting cysteine proteases,^[^ ^533^ ^]^ modulating the corticosterone levels in the treatment of anxiety‐related disorders and extend their neuroprotective role by inhibiting integrin avβ3 to modulate anxiety regulating pathways.^[^ ^536^ ^]^ Te‐NPs regulate the redox metabolism in oxidative stress conditions by scavenging H_2_O_2_, OH^.^ radicals and inducing apoptosis in the treatment of colon cancer.^[^ ^537^ ^]^ Embedding antioxidant Te‐NPs into electrospun fibers of polycaprolactone/gelatin, enhanced its wound healing properties, by targeting inflammation at the wounded site, leading to dose‐dependent collagen formation and modulation of redox enzymes (GSH, CAT, malondialdehyde (MDA)). This study shows the combinational immunomodulatory and tissue regenerative role of antioxidant Te‐based nanofibers.^[^ ^538^ ^]^

Tellurium nanowires (Te‐NWs) act as inorganic prodrug to mediate precise tumor ablation, by generating TeO_6_^6−^ molecules upon interacting with intracellular H_2_O_2_, which decreases intracellular GSH and consequently increasing ROS. Te‐NWs selectively target tumor cells by apoptosis and caspase‐independent autophagy without inducing host toxicity. After eliciting its immunomodulatory antitumor effects, Te‐NWs are easily cleared from the body by dissociating into smaller molecules.^[^ ^539^ ^]^ Further, hydrophilic ultra‐thin Te‐NWs with well‐defined mesostructures possessed extraordinary reversible photoelectric properties enhancing its potential in photothermal cancer therapy.^[^ ^540^ ^]^ Functionalized tellurium nanorods with polysaccharide‐protein complex were developed using the facile one‐pot hydrothermal synthetic system, exhibited remarkable hemocompatibility, low toxicity to normal cells and induced a dose‐dependent mitochondrial dysfunction and apoptosis by targeting intracellular ROS. The functionalized nanorods scavenged the free radicals: 2,2‐diphenyl‐1‐picrylhydrazylhydrate (DPPH) and 2,2′‐azinobis‐(3‐ethylbenzothiazoline‐6‐sulfonic acid radical cation (ABTS^.+^) exhibiting its immunomodulatory antioxidant properties.^[^ ^541^ ^]^ In summary,, tellurium in different nanoforms of holds promise in various therapeutics.

Together with sulfur and selenium, these 2D chalcogens exhibit significant immunomodulatory effects owing to its role in redox modulation, photothermal, antimicrobial and anti‐inflammatory properties.

### 2.5 Role of Group‐17 & 18 Elements in Fabricating Immunomodulatory 2D Biomaterials

The halogen group consist of fluorine (F), chlorine (Cl), bromine (Br), iodine (I), and astatine (At) and is known for its high reactivity, due to 7 valence electrons in its structure that needs only 1 more to complete its octet. Except for astatine, all the elements are non‐metallic and non‐radioactive. The halogen elements are mostly employed for synthesis processing, surface modification, and chemistry manipulation of various nanomaterials. For instance, fluoride‐based acids and salts are used in the etching of “A” from the MAX phase in the synthesis of 2D MXenes, affecting the surface termination and physiochemical properties of resultant MXenes. The group‐18, “noble gas” elements include helium (He), neon (Ne), argon (Ar), krypton (Kr), xenon (Xe), radon (Rn), and oganesson (Og), and are inert/non‐reactive due to full valence electron structure. These noble‐gas elements are mostly colourless, odourless, tasteless and non‐flammable making them useful applications requiring stable environment, such as fabrication, manipulation, and functionalization processes of synthesis of other 2D biomaterials. While halogens and noble gases contribute to the fabrication and functionalization of 2D nanomaterials, their direct applications as immunoengineered nanomaterials are rarely reported and primarily explored in theoretical and computational studies.

## 3 Conclusions and Future Prospects

This review comprehensively reports the group‐wise element by element exploration of the periodic table in the field of immunoengineering and its allied applications. The chemical diversity of the elements of the periodic table opens new avenues to utilize the existing research for the development of new innovative strategies. Hence, this review helps in the identification of the elements that extend its immunomodulatory role by its interaction with immune cells, induction of ROS/RNS and modulation of oxidative stress. This review also covers the elements that may have a potential role in the future to be used as viable immunomodulatory agents owing to their theranostic applications. So far, the transition metals in groups 3 to 12 have been extensively explored as 2D nanomaterials and studied for their immunomodulatory roles. As discussed, the 2D layered nanomaterials composed of the periodic chemical elements significantly outperform their thicker counterparts in the interms of physicochemical and biological properties for biomedical applications. Besides, the family of 2D nanomaterials beyond graphene have been significantly expanded over the past decade with the development of a large set of transition‐metal carbonatites MXenes, transition‐metal dichalcogenides, ultrathin organic crystals, metal‐organic frameworks, metal oxides, post‐transition metal chalcogenides, and mono‐elemental Xenes. These materials have garnered consistent attention to be used as newer nano‐agents for antimicrobial, anticancer, immunomodulatory, targeted delivery, and tissue engineering applications. More recently, smart immunoengineered nanosystems based on these biomaterials have been considered promising strategies towards the treatment of various inflammatory and infectious diseases including COVID‐19 infection.

Thus, in this review we have comprehensively discussed the advances in the preparation, physicochemical properties, and bio‐applications of 2D immunomodulatory materials to understand their mechanisms to interact with the immune cells. We have thoroughly evaluated and studied the immunomodulatory role of each element of the periodic table and categorized them based on their position in the periodic table and reported applications in a group‐wise fashion. To expand the scope of our work, we also discussed other biomedical applications of 2D materials for light‐activated cancer therapy and combined chemotherapy and summarized their representative biocompatibility in vitro and in vivo. It presents a new design of the periodic table by focusing on immunomodulatory elements and derived 2D biomaterials with a focus on immunoengineering mechanisms. This work will also pave the way towards the construction of new mono, hybrid, and composite nanosystems with tunable immunomodulatory properties for nanomedicine applications.

## Conflict of Interest

The authors declare no conflict of interest.

## Author Contributions

The study was conceptualized and designed by Alireza Rafieerad, Ahmad Amiri, Leena Regi Saleth and Sanjiv Dhingra. Alireza Rafieerad, Leena Regi Saleth, Soofia Khanahmadi, Keshav N Alagarsamy and Sanjiv Dhingra drafted the manuscript and designed Figures 1 and 2. All authors have read and approved the final version of this comprehensive review manuscript.

## References

[1] H. Yin , K. Xing , Y. Zhang , D. M. A. S. Dissanayake , Z. Lu , H. Zhao , Z. Zeng , J.‐H. Yun , D.‐C. Qi , Z. Yin , Chem. Soc. Rev 2021, 50, 6423. 34100047 10.1039/d0cs01146k

[2] B. R. Smith , S. S. Gambhir , Chem. Rev. 2017, 117, 901. 28045253 10.1021/acs.chemrev.6b00073

[3] Nanocarriers as an Emerging Platform for Cancer Therapy, Jenny Stanford Publishing, Singapore, 2020.

[4] B. Pelaz , C. Alexiou , R. A. Alvarez‐Puebla , F. Alves , A. M. Andrews , S. Ashraf , L. P. Balogh , L. Ballerini , A. Bestetti , C. Brendel , S. Bosi , M. Carril , W. C. W. Chan , C. Chen , X. Chen , X. Chen , Z. Cheng , D. Cui , J. Du , C. Dullin , A. Escudero , N. Feliu , M. Gao , M. George , Y. Gogotsi , A. Grünweller , Z. Gu , N. J. Halas , N. Hampp , R. K. Hartmann , ACS Nano 2017, 11, 2313. 28290206 10.1021/acsnano.6b06040 PMC5371978

[5] P. Chellan , P. J. Sadler , Philos. Trans. R. Soc., A 2015, 373, 20140182. 10.1098/rsta.2014.0182 PMC4342972 25666066

[6] R. J. Needham , P. J. Sadler , in The Periodic Table II: Catalytic, Materials, Biological and Medical Applications (Ed.: D. M. P. Mingos ), Springer International Publishing, Cham, 2019, pp. 175–201.

[7] L. P. Wackett , A. G. Dodge , L. B. M. Ellis , Appl. Environ. Microbiol. 2004, 70, 647. 14766537 10.1128/AEM.70.2.647-655.2004 PMC348800

[8] N. P. E. Barry , P. J. Sadler , Chem. Commun. 2013, 49, 5106. 10.1039/c3cc41143e 23636600

[9] H. Huang , W. Feng , Y. Chen , Chem. Soc. Rev. 2021, 50, 11381. 34661206 10.1039/d0cs01138j

[10] N. Rohaizad , C. C. Mayorga‐Martinez , M. Fojtů , N. M. Latiff , M. Pumera , Chem. Soc. Rev. 2021, 50, 619. 33206730 10.1039/d0cs00150c

[11] Nat. Nanotechnol. 2021, 16, 1.

[12] J. Lu , X. Liu , Y.‐P. Liao , F. Salazar , B. Sun , W. Jiang , C. H. Chang , J. Jiang , X. Wang , A. M. Wu , H. Meng , A. E. Nel , Nat. Commun. 2017, 8, 1811. 29180759 10.1038/s41467-017-01651-9 PMC5703845

[13] J. A. Hubbell , S. N. Thomas , M. A. Swartz , Nature 2009, 462, 449. 19940915 10.1038/nature08604

[14] C. Zheng , Q. Wang , Y. Wang , X. Zhao , K. Gao , Q. Liu , Y. Zhao , Z. Zhang , Y. Zheng , J. Cao , H. Chen , L. Shi , C. Kang , Y. Liu , Y. Lu , Adv. Mater. 2019, 31, 1902542. 10.1002/adma.201902542 31183900

[15] R. I. Lynch , E. C. Lavelle , Biochem. Pharmacol. 2022, 197, 114890. 34990595 10.1016/j.bcp.2021.114890

[16] J. Deng , J. Wang , J. Shi , H. Li , M. Lu , Z. Fan , Z. Gu , H. Cheng , Adv. Drug Delivery Rev. 2022, 180, 114039. 10.1016/j.addr.2021.114039 34742825

[17] G. A. Hodge , A. D. Maynard , D. M. Bowman , Science and Public Policy 2014, 41, 1.

[18] H. F. Zainal Abidin , K. H. Hassan , Z. A. Zainol , Nanoethics 2020, 14, 155.

[19] G. A. Hodge , D. M. Bowman , A. D. Maynard , International Handbook on Regulating Nanotechnologies 2010.

[20] K. W. Abbott , D. J. Sylvester , G. E. Marchant , International Handbook on Regulating Nanotechnologies 2010.

[21] A. E. Kokotovich , J. Kuzma , C. L. Cummings , K. Grieger , Nanoethics 2021, 15, 229.

[22] C. Oksel Karakus , E. Bilgi , D. A. Winkler , Nanotoxicology 2021, 15, 331. 33337941 10.1080/17435390.2020.1860265

[23] J. Amorós , S. Ravi , Phys. Chem. Liq. 2011, 49, 9.

[24] T. Hu , X. Mei , Y. Wang , X. Weng , R. Liang , M. Wei , Sci. Bull. 2019, 64, 1707. 10.1016/j.scib.2019.09.021 36659785

[25] W. Tao , X. Ji , X. Xu , M. A. Islam , Z. Li , S. Chen , P. E. Saw , H. Zhang , Z. Bharwani , Z. Guo , J. Shi , O. C. Farokhzad , Angew. Chem. 2017, 129, 12058. 10.1002/anie.201703657 PMC5608550 28640986

[26] J. Zhang , Y. Qin , Y. Chen , X. Zhao , J. Wang , Z. Wang , J. Li , J. Zhao , S. Liu , Z. Guo , W. Wei , J. Zhao , X. Wang , ACS Nano 2024, 18, 4398. 38275273 10.1021/acsnano.3c10432

[27] W. Chen , J. Ouyang , H. Liu , M. Chen , K. Zeng , J. Sheng , Z. Liu , Y. Han , L. Wang , J. Li , L. Deng , Y.‐N. Liu , S. Guo , Adv. Mater. 2017, 29, 1603864. 10.1002/adma.201603864 27882622

[28] X. Ji , N. Kong , J. Wang , W. Li , Y. Xiao , S. T. Gan , Y. Zhang , Y. Li , X. Song , Q. Xiong , S. Shi , Z. Li , W. Tao , H. Zhang , L. Mei , J. Shi , Adv. Mater. 2018, 30, 1803031. 10.1002/adma.201803031 PMC6338531 30019786

[29] T. Yin , J. Liu , Z. Zhao , Y. Zhao , L. Dong , M. Yang , J. Zhou , M. Huo , Adv. Funct. Mater. 2017, 27, 1604620.

[30] R. Chen , J. Zhang , Y. Wang , X. Chen , J. A. Zapien , C.‐S. Lee , Nanoscale 2015, 7, 17299. 26287769 10.1039/c5nr04436g

[31] L. Peng , X. Mei , J. He , J. Xu , W. Zhang , R. Liang , M. Wei , D. G. Evans , X. Duan , Adv. Mater. 2018, 30, 1707389. 10.1002/adma.201707389 29537662

[32] W. Fan , W. Bu , B. Shen , Q. He , Z. Cui , Y. Liu , X. Zheng , K. Zhao , J. Shi , Adv. Mater. 2015, 27, 4155. 26058562 10.1002/adma.201405141

[33] P. Shah , T. N. Narayanan , C.‐Z. Li , S. Alwarappan , Nanotechnology 2015, 26, 315102. 26183754 10.1088/0957-4484/26/31/315102

[34] H. Lin , S. Gao , C. Dai , Y. Chen , J. Shi , J. Am. Chem. Soc. 2017, 139, 16235. 29063760 10.1021/jacs.7b07818

[35] J. Shao , J. Zhang , C. Jiang , J. Lin , P. Huang , Chem. Eng. J. 2020, 400, 126009.

[36] G. Guan , X. Wang , B. Li , W. Zhang , Z. Cui , X. Lu , R. Zou , J. Hu , Nanoscale 2018, 10, 17902. 30226246 10.1039/c8nr06507a

[37] D. Zhang , W. Zheng , X. Li , A. Li , N. Ye , L. Zhang , Y. Liu , X. Liu , R. Zhang , M. Wang , J. Cheng , H. Yang , M. Gong , Carbon 2021, 178, 810.

[38] M. Gu , Z. Dai , X. Yan , J. Ma , Y. Niu , W. Lan , X. Wang , Q. Xu , J. Appl. Toxicol. 2021, 41, 745. 33048420 10.1002/jat.4085

[39] J.‐H. Jang , E.‐J. Lee , Materials 2021, 14, 4453.34442976

[40] W. Wu , H. Ge , L. Zhang , X. Lei , Y. Yang , Y. Fu , H. Feng , Chem. Res. Toxicol. 2020, 33, 2953. 33253550 10.1021/acs.chemrestox.0c00232

[41] B. Scheibe , J. K. Wychowaniec , M. Scheibe , B. Peplińska , M. Jarek , G. Nowaczyk , Ł. Przysiecka , ACS Biomater. Sci. Eng. 2019, 5, 6557. 33417807 10.1021/acsbiomaterials.9b01476

[42] G. K. Nasrallah , M. Al‐Asmakh , K. Rasool , K. A. Mahmoud , Environ. Sci.: Nano 2018, 5, 1002.

[43] M. A. Unal , F. Bayrakdar , L. Fusco , O. Besbinar , C. E. Shuck , S. Yalcin , M. T. Erken , A. Ozkul , C. Gurcan , O. Panatli , G. Y. Summak , C. Gokce , M. Orecchioni , A. Gazzi , F. Vitale , J. Somers , E. Demir , S. S. Yildiz , H. Nazir , J.‐C. Grivel , D. Bedognetti , A. Crisanti , K. C. Akcali , Y. Gogotsi , L. G. Delogu , A. Yilmazer , Nano Today 2021, 38, 101136. 33753982 10.1016/j.nantod.2021.101136 PMC7969865

[44] W. Feng , X. Han , H. Hu , M. Chang , L. Ding , H. Xiang , Y. Chen , Y. Li , Nat. Commun. 2021, 12, 2203. 33850133 10.1038/s41467-021-22278-x PMC8044242

[45] G. R. Fries , M. J. Zamzow , G. D. Colpo , N. Monroy‐Jaramillo , J. Quevedo , J. G. Arnold , C. L. Bowden , C. Walss‐Bass , Journal of Psychiatric Research 2020, 128, 38. 32516629 10.1016/j.jpsychires.2020.05.022 PMC7484018

[46] E. S. Takeuchi , R. A. Leising , MRS Bull. 2002, 27, 624.

[47] M. Sun , N. Stolte , J. Wang , J. Wei , P. Chen , Z. Xu , W. Wang , D. Pan , X. Bai , Small 2021, 17, 2101641. 10.1002/smll.202101641 34212489

[48] G. A. Sotiriou , WIREs Nanomedicine and Nanobiotechnology 2013, 5, 19. 22887856 10.1002/wnan.1190

[49] D. Li , X. Xie , Z. Yang , C. Wang , Z. Wei , P. Kang , Biomater. Sci. 2018, 6, 519. 29369309 10.1039/c7bm00975e

[50] B. Li , Y. Lei , Q. Hu , D. Li , H. Zhao , P. Kang , Biomed. Mater. 2021, 16, 065012. 10.1088/1748-605X/ac246e 34492640

[51] R. Machado‐Vieira , H. K. Manji , C. A. Zarate Jr , Bipolar Disorders 2009, 11, 92. 19538689 10.1111/j.1399-5618.2009.00714.x PMC2800957

[52] W. Young , Cell Transplant. 2009, 18, 951. 19523343 10.3727/096368909X471251

[53] R. F. Stolk , T. van der Poll , D. C. Angus , J. G. van der Hoeven , P. Pickkers , M. Kox , Am J. Respir. Crit. Care Med. 2016, 194, 550. 27398737 10.1164/rccm.201604-0862CP

[54] O. A. Madkhali , S. Sivagurunathan Moni , M. H. Sultan , H. A. Bukhary , M. Ghazwani , N. A. Alhakamy , A. M. Meraya , S. Alshahrani , S. S. Alqahtani , M. A. Bakkari , M. I. Alam , M. E. Elmobark , Sci. Rep. 2021, 11, 9914. 33972626 10.1038/s41598-021-89330-0 PMC8110975

[55] O. Maxwell , O. F. Oghenerukevwe , O. Adewoyin Olusegun , E. S. Joel , O. Arinze Daniel , A. Oluwasegun , H. O. Jonathan , T. O. Samson , N. Adeleye , O. M. Michael , A. Omeje Uchechukwu , A. Akinwumi Oluwasayo , A. Akinpelu , M. L. Akinyemi , O. Oladokun , Heliyon 2021, 7, e08470. 34926849 10.1016/j.heliyon.2021.e08470 PMC8649735

[56] M. B. Shakoor , R. Nawaz , F. Hussain , M. Raza , S. Ali , M. Rizwan , S.‐E. Oh , S. Ahmad , Sci. Total Environ. 2017, 601–602, 756. 10.1016/j.scitotenv.2017.05.223 28577410

[57] S. Kumar , W. Ahlawat , G. Bhanjana , S. Heydarifard , M. M. Nazhad , N. Dilbaghi , J. Nanosci. Nanotechnol. 2014, 14, 1838. 24749460 10.1166/jnn.2014.9050

[58] M. S. Mauter , I. Zucker , F. Perreault , J. R. Werber , J.‐H. Kim , M. Elimelech , Nat. Sustain. 2018, 1, 166.

[59] S. You , M. Ye , J. Xiong , Z. Hu , Y. Zhang , Y. Yang , C. C. Li , Small 2021, 17, 2102400. 10.1002/smll.202102400 34310031

[60] E. Navarrete , E. Cisternas , F. Dietrich , E. Muñoz , C. Heyser , A. Ramirez , Meet. Abstr. 2021, MA2021‐02, 241.

[61] P. Vashishtha , T. J. N. Hooper , Y. Fang , D. Kathleen , D. Giovanni , M. Klein , T. Chien Sum , S. G. Mhaisalkar , N. Mathews , T. White , Nanoscale 2021, 13, 59. 33346310 10.1039/d0nr08093d

[62] Y. Liu , Y. Tan , J. Wu , Journal of Asian Ceramic Societies 2021, 9, 323.

[63] H. M. A. Javed , M. I. Ahmad , W. Que , A. A. Qureshi , M. Sarfaraz , S. Hussain , M. Z. Iqbal , M. Zubair Nisar , M. Shahid , T. S. AlGarni , Surfaces and Interfaces 2021, 23, 101033.

[64] E. A. Daza , S. K. Misra , A. S. Schwartz‐Duval , A. Ohoka , C. Miller , D. Pan , ACS Appl. Mater. Interfaces 2016, 8, 26600. 27662498 10.1021/acsami.6b09887

[65] J. Yan , Y. Lu , G. Chen , M. Yang , Z. Gu , Chem. Soc. Rev. 2018, 47, 2518. 29557433 10.1039/C7CS00309A

[66] A. Heidari , K. Schmitt , M. Henderson , E. Besana , Dent Oral Maxillofac Res 2020, 6, 1.

[67] V. Selvaraj , B. Morri , L. M. Nair , H. Krishnan , J. Therm. Anal. Calorim. 2019, 137, 1527.

[68] E. Pajuste , G. Kizane , L. Avotina , Materials Science 2015, 21, 215.

[69] J. I. Prado , J. P. Vallejo , L. Lugo , Powder Technol. 2022, 397, 117082.

[70] S. Abinaya , H. P. Kavitha , M. Prakash , A. Muthukrishnaraj , Sustainable Chemistry and Pharmacy 2021, 19, 100368.

[71] Y. He , M. Yao , J. Zhou , J. Xie , C. Liang , D. Yin , S. Huang , Y. Zhang , F. Peng , S. Cheng , Regenerative Biomaterials 2022, 9, rbac027. 35592137 10.1093/rb/rbac027 PMC9113411

[72] F. Wahid , X.‐J. Zhao , S.‐R. Jia , H. Bai , C. Zhong , Composites, Part B 2020, 200, 108208.

[73] R. K. Das , V. L. Pachapur , L. Lonappan , M. Naghdi , R. Pulicharla , S. Maiti , M. Cledon , L. M. A. Dalila , S. J. Sarma , S. K. Brar , Nanotechnol. Environ. Eng. 2017, 2, 18.

[74] E. N. Zare , P. Makvandi , A. Borzacchiello , F. R. Tay , B. Ashtari , V. V. T. Padil , Chem. Commun. 2019, 55, 14871. 10.1039/c9cc08207g 31776528

[75] S. Narendhran , M. Manikandan , P. B. Shakila , Bull. Mater. Sci. 2019, 42, 133.

[76] E. R. Hopper , T. M. R. Wayman , J. Asselin , B. Pinho , C. Boukouvala , L. Torrente‐Murciano , E. Ringe , J. Phys. Chem. C 2022, 126, 563. 10.1021/acs.jpcc.1c07544 PMC8762659 35059097

[77] A. Truskewycz , V. K. Truong , A. S. Ball , S. Houshyar , N. Nassar , H. Yin , B. J. Murdoch , I. Cole , ACS Appl. Mater. Interfaces 2021, 13, 27904. 34105937 10.1021/acsami.1c05908

[78] M. Laurenti , A. Al Subaie , M.‐N. Abdallah , A. R. G. Cortes , J. L. Ackerman , H. Vali , K. Basu , Y. L. Zhang , M. Murshed , S. Strandman , J. Zhu , N. Makhoul , J. E. Barralet , F. Tamimi , Nano Lett. 2016, 16, 4779. 27280476 10.1021/acs.nanolett.6b00636

[79] N. Demirkol , Advanced Nano‐Bio‐Materials and Devices – AdvNanoBioM&D 2019.

[80] M. Fan , Y. Wen , D. Ye , Z. Jin , P. Zhao , D. Chen , X. Lu , Q. He , Adv. Healthcare Mater. 2019, 8, 1900157. 10.1002/adhm.201900157 30968583

[81] N. Saglam , F. Korkusuz , R. Prasad , Eds., Nanotechnology Applications in Health and Environmental Sciences, Springer International Publishing, Cham, 2021.

[82] H. Mitwalli , R. AlSahafi , E. G. Albeshir , Q. Dai , J. Sun , T. W. Oates , M. A. S. Melo , H. H. K. Xu , M. D. Weir , Journal of Dentistry 2021, 113, 103789. 34455017 10.1016/j.jdent.2021.103789

[83] M. Naguib , M. Kurtoglu , V. Presser , J. Lu , J. Niu , M. Heon , L. Hultman , Y. Gogotsi , M. W. Barsoum , Adv. Mater. 2011, 23, 4248. 21861270 10.1002/adma.201102306

[84] K. R. G. Lim , M. Shekhirev , B. C. Wyatt , B. Anasori , Y. Gogotsi , Z. W. Seh , Nat. Synth 2022, 1, 601.

[85] A. Iqbal , F. Shahzad , K. Hantanasirisakul , M.‐K. Kim , J. Kwon , J. Hong , H. Kim , D. Kim , Y. Gogotsi , C. M. Koo , Science 2020, 369, 446. 32703878 10.1126/science.aba7977

[86] M. Soleymaniha , M.‐A. Shahbazi , A. R. Rafieerad , A. Maleki , A. Amiri , Adv. Healthcare Mater. 2019, 8, 1801137. 10.1002/adhm.201801137 30362268

[87] A. Maleki , M. Ghomi , N. Nikfarjam , M. Akbari , E. Sharifi , M.‐A. Shahbazi , M. Kermanian , M. Seyedhamzeh , E. Nazarzadeh Zare , M. Mehrali , O. Moradi , F. Sefat , V. Mattoli , P. Makvandi , Y. Chen , Adv. Funct. Mater. 2022, 32, 2203430.

[88] K. Huang , Z. Li , J. Lin , G. Han , P. Huang , Chem. Soc. Rev. 2018, 47, 5109. 29667670 10.1039/c7cs00838d

[89] B. Lu , Z. Zhu , B. Ma , W. Wang , R. Zhu , J. Zhang , Small 2021, 17, 2100946. 10.1002/smll.202100946 34323354

[90] H. Lin , Y. Chen , J. Shi , Adv. Sci. 2018, 5, 1800518. 10.1002/advs.201800518 PMC6193163 30356929

[91] A. Rafieerad , W. Yan , G. L. Sequiera , N. Sareen , E. Abu‐El‐Rub , M. Moudgil , S. Dhingra , Adv. Healthcare Mater. 2019, 8, 1970067. 10.1002/adhm.201900569 31265217

[92] S. R. K. C. Indukuri , C. Frydendahl , J. Bar‐David , N. Mazurski , U. Levy , ACS Appl. Nano Mater. 2020, 3, 10226.

[93] F. Wang , S. Li , M. A. Bissett , I. A. Kinloch , Z. Li , R. J. Young , 2D Mater. 2020, 7, 045022.

[94] W. Liu , X. Song , Q. Jiang , W. Guo , J. Liu , X. Chu , Z. Lei , Nanomaterials 2024, 14, 1064. 38998669 10.3390/nano14131064 PMC11243522

[95] M. Yang , J. Li , P. Gu , X. Fan , Bioactive Materials 2021, 6, 1973. 33426371 10.1016/j.bioactmat.2020.12.010 PMC7773537

[96] H.‐C. “Joe” Zhou , S. Kitagawa , Chem. Soc. Rev. 2014, 43, 5415. 25011480 10.1039/c4cs90059f

[97] P. D. Fernandes , F. D. Magalhães , R. F. Pereira , A. M. Pinto , Polymers 2023, 15, 1490.36987269

[98] Q. Li , Y. Liu , Y. Zhang , W. Jiang , J. Controlled Release 2022, 347, 183. 10.1016/j.jconrel.2022.05.003 35526612

[99] M. Haseli , L. Pinzon‐Herrera , X. Hao , S. R. Wickramasinghe , J. Almodovar , Langmuir 2023, 39, 16472. 37944116 10.1021/acs.langmuir.3c02355

[100] Z. Xu , K. Ni , J. Mao , T. Luo , W. Lin , Adv. Mater. 2021, 33, 2104249. 10.1002/adma.202104249 PMC8492529 34432917

[101] L. Cao , C. Wang , ACS Cent. Sci. 2020, 6, 2149. 33376778 10.1021/acscentsci.0c01150 PMC7760065

[102] K. Ni , G. Lan , C. Chan , X. Duan , N. Guo , S. S. Veroneau , R. R. Weichselbaum , W. Lin , Matter 2019, 1, 1331. 32832885 PMC7442115

[103] T. Hu , Z. Gu , G. R. Williams , M. Strimaite , J. Zha , Z. Zhou , X. Zhang , C. Tan , R. Liang , Chem. Soc. Rev. 2022, 51, 6126. 35792076 10.1039/d2cs00236a

[104] C. Zhu , J. Jiang , Y. Jia , Z. P. Xu , L. Zhang , Acc. Mater. Res. 2023, 4, 758.

[105] T. Hu , W. Shen , F. Meng , S. Yang , S. Yu , H. Li , Q. Zhang , L. Gu , C. Tan , R. Liang , Adv. Mater. 2023, 35, 2209692. 10.1002/adma.202209692 36780890

[106] R. Gao , X. Mei , D. Yan , R. Liang , M. Wei , Nat. Commun. 2018, 9, 2798. 30022060 10.1038/s41467-018-05223-3 PMC6052022

[107] R. Tian , D. Yan , M. Wei , in P L Materials (Eds.: D. Yan , M. Wei ), Springer International Publishing, Cham, 2015, pp. 1–68.

[108] S. Yu , G. Choi , J.‐H. Choy , Nanomaterials 2023, 13, 1102. 36985996 10.3390/nano13061102 PMC10058705

[109] W. W. Monafo , S. N. Tandon , V. H. Ayvazian , J. Tuchschmidt , A. M. Skinner , F. Deitz , Surgery 1976, 80, 465. 135364

[110] L. Monafo , Panminerva Med. 1983, 25, 151. 6669387

[111] C. L. Fox , W. W. Monafo , V. H. Ayvazian , A. M. Skinner , S. Modak , J. Stanford , C. Condict , Surg. Gynecol. Obstet. 1977, 144, 668. 850849

[112] B. G. Sparkes , Vaccine 1993, 11, 504. 8488699 10.1016/0264-410x(93)90218-m

[113] M. Naseri‐Nosar , S. Farzamfar , H. Sahrapeyma , S. Ghorbani , F. Bastami , A. Vaez , M. Salehi , Mater. Sci. Eng., C 2017, 81, 366. 10.1016/j.msec.2017.08.013 28887985

[114] L. Cao , G. Shao , F. Ren , M. Yang , Y. Nie , Q. Peng , P. Zhang , Drug Delivery 2021, 28, 390. 33594917 10.1080/10717544.2020.1858998 PMC7894430

[115] E. Barker , J. Shepherd , I. O. Asencio , Molecules 2022, 27, 2678. 35566026 10.3390/molecules27092678 PMC9104093

[116] X. Li , M. Qi , X. Sun , M. D. Weir , F. R. Tay , T. W. Oates , B. Dong , Y. Zhou , L. Wang , H. H. K. Xu , Acta Biomater. 2019, 94, 627. 31212111 10.1016/j.actbio.2019.06.023

[117] H. F. Hammouda , M. M. Farag , M. M. F. El Deftar , M. Abdel‐Gabbar , B. M. Mohamed , J. Genet. Eng. & Biotechnol. 2022, 20, 33. 35192077 10.1186/s43141-022-00302-x PMC8864049

[118] A. Asati , S. Santra , C. Kaittanis , S. Nath , J. M. Perez , Angew. Chem. 2009, 121, 2344. 10.1002/anie.200805279 PMC2923475 19130532

[119] C. Korsvik , S. Patil , S. Seal , W. T. Self , Chem. Commun. 2007, 1056. 10.1039/b615134e 17325804

[120] T. Pirmohamed , J. M. Dowding , S. Singh , B. Wasserman , E. Heckert , A. S. Karakoti , J. E. S. King , S. Seal , W. T. Self , Chem. Commun. 2010, 46, 2736. 10.1039/b922024k PMC3038687 20369166

[121] Z. Wang , X. Shen , X. Gao , Y. Zhao , Nanoscale 2019, 11, 13289. 31287483 10.1039/c9nr03473k

[122] A. Asati , S. Santra , C. Kaittanis , J. M. Perez , ACS Nano 2010, 4, 5321. 20690607 10.1021/nn100816s PMC2947560

[123] S. Das , S. Singh , J. M. Dowding , S. Oommen , A. Kumar , T. X. T. Sayle , S. Saraf , C. R. Patra , N. E. Vlahakis , D. C. Sayle , W. T. Self , S. Seal , Biomaterials 2012, 33, 7746. 22858004 10.1016/j.biomaterials.2012.07.019 PMC4590782

[124] M. S. Lord , B. Tsoi , C. Gunawan , W. Y. Teoh , R. Amal , J. M. Whitelock , Biomaterials 2013, 34, 8808. 23942211 10.1016/j.biomaterials.2013.07.083

[125] X. Pang , J. Li , Y. Zhao , D. Wu , Y. Zhang , B. Du , H. Ma , Q. Wei , ACS Appl. Mater. Interfaces 2015, 7, 19260. 26271682 10.1021/acsami.5b05185

[126] S. Yu , G. Zou , Q. Wei , Talanta 2016, 156–157, 11. 10.1016/j.talanta.2016.04.050 27260429

[127] N. Pachauri , K. Dave , A. Dinda , P. R. Solanki , J. Mater. Chem. B 2018, 6, 3000. 32254335 10.1039/c8tb00653a

[128] Y. Li , Y. Zhang , F. Li , J. Feng , M. Li , L. Chen , Y. Dong , Biosens. Bioelectron. 2017, 92, 33. 28182976 10.1016/j.bios.2017.01.065

[129] H. Xie , C. Zhang , Z. Gao , Anal. Chem. 2004, 76, 1611. 15018558 10.1021/ac0350965

[130] K.‐J. Feng , Y.‐H. Yang , Z.‐J. Wang , J.‐H. Jiang , G.‐L. Shen , R.‐Q. Yu , Talanta 2006, 70, 561. 18970808 10.1016/j.talanta.2006.01.009

[131] C. Shao , A. Shen , M. Zhang , X. Meng , C. Song , Y. Liu , X. Gao , P. Wang , W. Bu , ACS Nano 2018, 12, 12629. 30495921 10.1021/acsnano.8b07387

[132] L. Zeng , H. Zhao , Y. Zhu , S. Chen , Y. Zhang , D. Wei , J. Sun , H. Fan , J. Mater. Chem. B 2020, 8, 4093. 32249879 10.1039/d0tb00080a

[133] D. E. J. G. J. Dolmans , D. Fukumura , R. K. Jain , Nat. Rev. Cancer 2003, 3, 380. 12724736 10.1038/nrc1071

[134] C. Xu , Y. Lin , J. Wang , L. Wu , W. Wei , J. Ren , X. Qu , Adv. Healthcare Mater. 2013, 2, 1591. 10.1002/adhm.201200464 23630084

[135] M. S. Wason , H. Lu , L. Yu , S. K. Lahiri , D. Mukherjee , C. Shen , S. Das , S. Seal , J. Zhao , Cancers 2018, 10, 303. 30200491 10.3390/cancers10090303 PMC6162528

[136] F. Chen , X. H. Zhang , X. D. Hu , W. Zhang , Z. C. Lou , L. H. Xie , P. D. Liu , H. Q. Zhang , IJN 2015, 10, 4957. 26316742 10.2147/IJN.S82980 PMC4542556

[137] G. Goujon , V. Baldim , C. Roques , N. Bia , J. Seguin , B. Palmier , A. Graillot , C. Loubat , N. Mignet , I. Margaill , J.‐F. Berret , V. Beray‐Berthat , Adv. Healthcare Mater. 2021, 10, 2100059. 10.1002/adhm.202100059 33890419

[138] S. I. Han , S. Lee , M. G. Cho , J. M. Yoo , M. H. Oh , B. Jeong , D. Kim , O. K. Park , J. Kim , E. Namkoong , J. Jo , N. Lee , C. Lim , M. Soh , Y.‐E. Sung , J. Yoo , K. Park , T. Hyeon , Adv. Mater. 2020, 32, 2001566. 10.1002/adma.202001566 32520432

[139] A. Torres‐Romero , M. Cajero‐Juárez , R. E. Nuñez‐Anita , M. E. Contreras‐García , J. Nanosci. Nanotechnol. 2020, 20, 3971. 31968410 10.1166/jnn.2020.17206

[140] C. Tapeinos , M. Battaglini , M. Prato , G. La Rosa , A. Scarpellini , G. Ciofani , ACS Omega 2018, 3, 8952. 31459028 10.1021/acsomega.8b01060 PMC6644480

[141] C. Xu , Q. Feng , H. Yang , G. Wang , L. Huang , Q. Bai , C. Zhang , Y. Wang , Y. Chen , Q. Cheng , M. Chen , Y. Han , Z. Yu , M. S. Lesniak , Y. Cheng , Adv. Sci. 2018, 5, 1800382. 10.1002/advs.201800382 PMC6193170 30356957

[142] B. C. Wilson , R. A. Weersink , Photochem. Photobiol. 2020, 96, 219. 31769516 10.1111/php.13184

[143] Y. Wang , G. Wei , X. Zhang , X. Huang , J. Zhao , X. Guo , S. Zhou , Small 2018, 14, 1702994. 10.1002/smll.201702994 29205795

[144] X. Zhu , Y. Gong , Y. Liu , C. Yang , S. Wu , G. Yuan , X. Guo , J. Liu , X. Qin , Biomaterials 2020, 242, 119923. 32145506 10.1016/j.biomaterials.2020.119923

[145] N. Feng , Y. Liu , X. Dai , Y. Wang , Q. Guo , Q. Li , RSC Adv. 2022, 12, 1486. 35425183 10.1039/d1ra05407d PMC8979138

[146] D. Pozo , F. J. Quintana , Nanoparticle‐Mediated Signaling Rewiring and Reprogramming of Immune Responses, Frontiers Media, SA, 2022. 10.3389/fimmu.2022.927733 PMC9134789 35634330

[147] B. C. Schanen , S. Das , C. M. Reilly , W. L. Warren , W. T. Self , S. Seal , D. R. D. Iii , PLoS One 2013, 8, e62816. 23667525 10.1371/journal.pone.0062816 PMC3648566

[148] A. Patel , J. Kosanovich , S. Sansare , S. Balmuri , V. Sant , K. M. Empey , S. Sant , 2022. 10.1016/j.bioactmat.2022.12.005 PMC9791695 36606255

[149] W.‐C. Lien , X.‐R. Zhou , Y.‐J. Liang , C. T.‐S. Ching , C.‐Y. Wang , F.‐I. Lu , H.‐C. Chang , F.‐H. Lin , H.‐M. D. Wang , Bioeng. Transl. Med. 2023, 8, e10346. 36684074 10.1002/btm2.10346 PMC9842028

[150] Lett Appl NanoBioSci 2022, 12, 12.

[151] S. W. Choi , J. Kim , ACS Appl. Nano Mater 2020, 3, 1043.

[152] L. V. Kalia , A. E. Lang , Lancet 2015, 386, 896. 25904081 10.1016/S0140-6736(14)61393-3

[153] A. C. Kaushik , S. Bharadwaj , S. Kumar , D.‐Q. Wei , Sci. Rep. 2018, 8, 9169. 29907754 10.1038/s41598-018-27580-1 PMC6003935

[154] B. M, D. L, D. C.d, H. K.s, R. B.a, TechConnect Briefs 2011, 3, 451.

[155] V. Jackson‐Lewis , S. Przedborski , Nat. Protoc. 2007, 2, 141. 17401348 10.1038/nprot.2006.342

[156] M. A. E. Hegazy , H. M. Maklad , D. A. A. Elmonsif , F. Y. Elnozhy , M. A. Alqubiea , F. A. Alenezi , O. M. A. Abbas , M. M. A. Abbas , Alexandria J. Med. 2017, 53, 351.

[157] J. Machhi , P. Yeapuri , M. Markovic , M. Patel , W. Yan , Y. Lu , J. D. Cohen , M. Hasan , M. M. Abdelmoaty , Y. Zhou , H. Xiong , X. Wang , R. L. Mosley , H. E. Gendelman , B. D. Kevadiya , ACS Chem. Neurosci. 2022, 13, 1232. 35312284 10.1021/acschemneuro.1c00847 PMC9227977

[158] F. Zeng , Y. Wu , X. Li , X. Ge , Q. Guo , X. Lou , Z. Cao , B. Hu , N. J. Long , Y. Mao , C. Li , Angew. Chem. 2018, 130, 5910. 10.1002/anie.201802309 29575461

[159] S. A. Saeed , K. F. Shad , T. Saleem , F. Javed , M. U. Khan , Exp. Brain Res. 2007, 182, 1. 17665180 10.1007/s00221-007-1050-9

[160] A. Y. Estevez , S. Pritchard , K. Harper , J. W. Aston , A. Lynch , J. J. Lucky , J. S. Ludington , P. Chatani , W. P. Mosenthal , J. C. Leiter , S. Andreescu , J. S. Erlichman , Free Radic. Biol. Med. 2011, 51, 1155. 21704154 10.1016/j.freeradbiomed.2011.06.006

[161] J. M. Dowding , S. Seal , W. T. Self , Drug Deliv. and Transl. Res. 2013, 3, 375. 23936755 10.1007/s13346-013-0136-0 PMC3736600

[162] C. K. Kim , T. Kim , I.‐Y. Choi , M. Soh , D. Kim , Y.‐J. Kim , H. Jang , H.‐S. Yang , J. Y. Kim , H.‐K. Park , S. P. Park , S. Park , T. Yu , B.‐W. Yoon , S.‐H. Lee , T. Hyeon , Angew. Chem., Int. Ed. 2012, 51, 11039. 10.1002/anie.201203780 22968916

[163] T. Zhang , C. Li , J. Jia , J. Chi , D. Zhou , J. Li , X. Liu , J. Zhang , L. Yi , Curr. Med. Sci. 2018, 38, 144. 30074164 10.1007/s11596-018-1858-5

[164] A. M. Naidech , Am J. Respir. Crit. Care Med. 2011, 184, 998. 22167847 10.1164/rccm.201103-0475CI PMC3361326

[165] J. van Gijn , G. J. E. Rinkel , Brain 2001, 124, 249. 11157554 10.1093/brain/124.2.249

[166] R. F. Keep , Y. Hua , G. Xi , Lancet Neurol. 2012, 11, 720. 22698888 10.1016/S1474-4422(12)70104-7 PMC3884550

[167] D.‐W. Kang , C. K. Kim , H.‐G. Jeong , M. Soh , T. Kim , I.‐Y. Choi , S.‐K. Ki , D. Y. Kim , W. Yang , T. Hyeon , S.‐H. Lee , Nano Res. 2017, 10, 2743.

[168] R. E. Ayer , J. H. Zhang , in Cerebral Vasospasm (2008, Eds.: T. Kırış , J. H. Zhang ), Springer, Vienna, pp. 33.

[169] H.‐G. Jeong , B. G. Cha , D.‐W. Kang , D. Y. Kim , S. K. Ki , S. I. Kim , J. hee Han , W. Yang , C. K. Kim , J. Kim , S.‐H. Lee , Stroke 2018, 49, 3030. 30571409 10.1161/STROKEAHA.118.022631

[170] S. Parween , P. K. Gupta , V. S. Chauhan , Vaccine 2011, 29, 2451. 21288801 10.1016/j.vaccine.2011.01.014

[171] J. J. Ryan , H. R. Bateman , A. Stover , G. Gomez , S. K. Norton , W. Zhao , L. B. Schwartz , R. Lenk , C. L. Kepley , J. Immunol. 2007, 179, 665. 17579089 10.4049/jimmunol.179.1.665

[172] Q. Jiao , L. Li , Q. Mu , Q. Zhang , Biomed Res. Int. 2014, 2014, e426028. 10.1155/2014/426028 PMC4052466 24949448

[173] C. J. Wingard , D. M. Walters , B. L. Cathey , S. C. Hilderbrand , P. Katwa , S. Lin , P. C. Ke , R. Podila , A. Rao , R. M. Lust , J. M. Brown , Nanotoxicology 2011, 5, 531. 21043986 10.3109/17435390.2010.530004 PMC3208763

[174] J. Y. Ma , H. Zhao , R. R. Mercer , M. Barger , M. Rao , T. Meighan , D. Schwegler‐Berry , V. Castranova , J. K. Ma , Nanotoxicology 2011, 5, 312. 20925443 10.3109/17435390.2010.519835

[175] W.‐S. Cho , R. Duffin , C. A. Poland , S. E. M. Howie , W. MacNee , M. Bradley , I. L. Megson , K. Donaldson , Environ. Health Perspect. 2010, 118, 1699. 20729176 10.1289/ehp.1002201 PMC3002189

[176] A. Srinivas , P. J. Rao , G. Selvam , P. B. Murthy , P. N. Reddy , Toxicol. Lett. 2011, 205, 105. 21624445 10.1016/j.toxlet.2011.05.1027

[177] E.‐J. Park , W.‐S. Cho , J. Jeong , J. Yi , K. Choi , Y. Kim , K. Park , J. Health Sci. 2010, 56, 387.

[178] M. J. Akhtar , M. Ahamed , H. A. Alhadlaq , J. King Saud. Univ. Sci. 2022, 34, 102291.

[179] M. Auguste , T. Balbi , M. Montagna , R. Fabbri , M. Sendra , J. Blasco , L. Canesi , Comp. Biochem. Physiol. C Toxicol. Pharmacol. 2019, 219, 95. 30797983 10.1016/j.cbpc.2019.02.006

[180] C. Ciacci , B. Canonico , D. Bilaniĉovă , R. Fabbri , K. Cortese , G. Gallo , A. Marcomini , G. Pojana , L. Canesi , PLoS One 2012, 7, e36937. 22606310 10.1371/journal.pone.0036937 PMC3350491

[181] M. Naguib , M. W. Barsoum , Y. Gogotsi , Adv. Mater. 2021, 33, 2103393. 10.1002/adma.202103393 34396592

[182] Q. Xue , H. Zhang , M. Zhu , Z. Pei , H. Li , Z. Wang , Y. Huang , Y. Huang , Q. Deng , J. Zhou , S. Du , Q. Huang , C. Zhi , Adv. Mater. 2017, 29, 1604847. 10.1002/adma.201604847 28185336

[183] Y. Lei , W. Zhao , Y. Zhang , Q. Jiang , J.‐H. He , A. J. Baeumner , O. S. Wolfbeis , Z. L. Wang , K. N. Salama , H. N. Alshareef , Small 2019, 15, 1901190. 10.1002/smll.201901190 30957964

[184] A. Sinha , H. Z. Dhanjai , Y. Huang , X. Lu , J. Chen , R. Jain , TrAC, Trends Anal. Chem. 2018, 105, 424.

[185] J. L. Hart , K. Hantanasirisakul , A. C. Lang , B. Anasori , D. Pinto , Y. Pivak , J. T. van Omme , S. J. May , Y. Gogotsi , M. L. Taheri , Nat. Commun. 2019, 10, 522. 30705273 10.1038/s41467-018-08169-8 PMC6355901

[186] A. Rafieerad , A. Amiri , G. L. Sequiera , W. Yan , Y. Chen , A. A. Polycarpou , S. Dhingra , Adv. Funct. Mater. 2021, 31, 2100015. 35264918 10.1002/adfm.202100015 PMC8889894

[187] M. Ghidiu , S. Kota , J. Halim , A. W. Sherwood , N. Nedfors , J. Rosen , V. N. Mochalin , M. W. Barsoum , Chem. Mater. 2017, 29, 1099.

[188] H. Liu , X. Xing , Y. Tan , H. Dong , Nanophotonics 2022, 11, 4977. 39634292 10.1515/nanoph-2022-0550 PMC11501147

[189] X. Chen , J. Li , G. Pan , W. Xu , J. Zhu , D. Zhou , D. Li , C. Chen , G. Lu , H. Song , Sens. Actuators, B 2019, 289, 131.

[190] L. Zhou , F. Wu , J. Yu , Q. Deng , F. Zhang , G. Wang , Carbon 2017, 118, 50.

[191] H. Lin , X. Wang , L. Yu , Y. Chen , J. Shi , Nano Lett. 2017, 17, 384. 28026960 10.1021/acs.nanolett.6b04339

[192] C. Dai , H. Lin , G. Xu , Z. Liu , R. Wu , Y. Chen , Chem. Mater. 2017, 29, 8637.

[193] G. Liu , J. Zou , Q. Tang , X. Yang , Y. Zhang , Q. Zhang , W. Huang , P. Chen , J. Shao , X. Dong , ACS Appl. Mater. Interfaces 2017, 9, 40077. 29099168 10.1021/acsami.7b13421

[194] X.‐H. Zha , K. Luo , Q. Li , Q. Huang , J. He , X. Wen , S. Du , EPL 2015, 111, 26007.

[195] A. Szuplewska , D. Kulpińska , A. Dybko , A. M. Jastrzębska , T. Wojciechowski , A. Rozmysłowska , M. Chudy , I. Grabowska‐Jadach , W. Ziemkowska , Z. Brzózka , A. Olszyna , Mater. Sci. Eng., C 2019, 98, 874. 10.1016/j.msec.2019.01.021 30813093

[196] D.‐Y. Zhang , H. Liu , M. R. Younis , S. Lei , Y. Chen , P. Huang , J. Lin , J. Nanobiotechnol. 2022, 20, 1. 10.1186/s12951-022-01253-8 PMC8796495 35090484

[197] X. Han , J. Huang , H. Lin , Z. Wang , P. Li , Y. Chen , Adv. Healthcare Mater. 2018, 7, 1701394. 10.1002/adhm.201701394 29405649

[198] C. Xing , S. Chen , X. Liang , Q. Liu , M. Qu , Q. Zou , J. Li , H. Tan , L. Liu , D. Fan , H. Zhang , ACS Appl. Mater. Interfaces 2018, 10, 27631. 30058793 10.1021/acsami.8b08314

[199] A. Zamhuri , G. P. Lim , N. L. Ma , K. S. Tee , C. F. Soon , Biomed. Eng. Online 2021, 20, 33. 33794899 10.1186/s12938-021-00873-9 PMC8017618

[200] A. Rafieerad , W. Yan , G. L. Sequiera , N. Sareen , E. Abu‐El‐Rub , M. Moudgil , S. Dhingra , Adv. Healthcare Mater. 2019, 8, 1900569. 10.1002/adhm.201900569 31265217

[201] W. Yan , A. Rafieerad , K. N. Alagarsamy , L. R. Saleth , R. C. Arora , S. Dhingra , Nano Today 2023, 48, 101706. 10.1016/j.nantod.2022.101706 PMC10181944 37187503

[202] H. Ding , A. Ahmed , K. Shen , L. Sun , Aggregate 2022, 3, e174.

[203] T. He , B. Ni , S. Zhang , Y. Gong , H. Wang , L. Gu , J. Zhuang , W. Hu , X. Wang , Small 2018, 14, 1703929. 10.1002/smll.201703929 29532997

[204] S. Arun Kumar , B. Balasubramaniam , S. Bhunia , M. K. Jaiswal , K. Verma , A. K. Prateek , R. K. Gupta , A. K. Gaharwar , Wires Nanomed. Nanobi. 2021, 13, e1674. 10.1002/wnan.1674 PMC13050496 33137846

[205] H. B. Halima , N. Zine , J. Gallardo‐González , A. E. Aissari , M. Sigaud , A. Alcacer , J. Bausells , A. Errachid , in 2019 20th International Conference on Solid‐State Sensors, Actuators and Microsystems & Eurosensors XXXIII (TRANSDUCERS & EUROSENSORS XXXIII) , 2019, pp. 1067–1070.

[206] L. Cao , Z. Lin , W. Shi , Z. Wang , C. Zhang , X. Hu , C. Wang , W. Lin , J. Am. Chem. Soc. 2017, 139, 7020. 28467852 10.1021/jacs.7b02470

[207] M. Xu , S.‐S. Yang , Z.‐Y. Gu , Chemistry – A European Journal 2018, 24, 15131. 30063265 10.1002/chem.201800556

[208] F. Ahmad , T. Muhmood , A. Mahmood , Nano Ex 2020, 1, 030006.

[209] J. A. Field , A. Luna‐Velasco , S. A. Boitano , F. Shadman , B. D. Ratner , C. Barnes , R. Sierra‐Alvarez , Chemosphere 2011, 84, 1401. 21605889 10.1016/j.chemosphere.2011.04.067

[210] L. Maggiorella , G. Barouch , C. Devaux , A. Pottier , E. Deutsch , J. Bourhis , E. Borghi , L. Levy , Future Oncol. 2012, 8, 1167. 23030491 10.2217/fon.12.96

[211] J. Li , W. Li , L. Xie , W. Sang , G. Wang , Z. Zhang , B. Li , H. Tian , J. Yan , Y. Tian , Z. Li , Q. Fan , L. Yu , Y. Dai , Chem. Commun. 2021, 57, 11473. 10.1039/d1cc04628d 34652356

[212] W. Sang , L. Xie , G. Wang , J. Li , Z. Zhang , B. Li , S. Guo , C.‐X. Deng , Y. Dai , Adv. Sci. 2021, 8, 2003338. 10.1002/advs.202003338 PMC7887592 33643804

[213] T. De Baere , M. Pracht , Y. Rolland , J. Durand‐Labrunie , N. Jaksic , F. Nguyen , J.‐P. Bronowicki , V. Vendrely , V. Croisé‐Laurent , E. Rio , S. Le Sourd , P. Said , P. Gustin , C. Perret , D. Peiffert , E. Deutsch , E. Chajon , JCO 2021, 39, 319.

[214] M. Pracht , E. Chajon , Y. Rolland , T. de Baere , F. Nguyen , J.‐P. Bronowicki , V. Vendrely , A. Sa Cunha , A.‐S. Baumann , V. Croisé‐Laurent , E. Rio , P. Said , S. Le Sourd , P. Gustin , C. Perret , D. Peiffert , E. Deutsch , Ann. Oncol. 2019, 30, v291.

[215] L. Maggiorella , G. Barouch , C. Devaux , A. Pottier , L. Levy , E. Deutsch , J. Bourhis , E. Borghi , Eur. J. Cancer 2011, 47, S189. 23030491 10.2217/fon.12.96

[216] Y. Liu , P. Zhang , F. Li , X. Jin , J. Li , W. Chen , Q. Li , Theranostics 2018, 8, 1824. 29556359 10.7150/thno.22172 PMC5858503

[217] A. Darmon , P. Zhang , J. Marill , N. Mohamed Anesary , J. Da silva , S. Paris , Cancer Cell Int. 2022, 22, 208. 35659676 10.1186/s12935-022-02615-w PMC9164428

[218] J. Galon , M. Laé , Z. Papai , P. Rochaix , L. C. Mangel , B. Mlecnik , F. Hermitte , Z. Sapi , M. Delannes , T. Tornoczky , A. Vincent‐Salomon , S. Bonvalot , JCO 2017, 35, e14615.

[219] S. Bonvalot , P. L. Rutkowski , J. Thariat , S. Carrère , A. Ducassou , M.‐P. Sunyach , P. Agoston , A. Hong , A. Mervoyer , M. Rastrelli , V. Moreno , R. K. Li , B. Tiangco , A. C. Herraez , A. Gronchi , L. Mangel , T. Sy‐Ortin , P. Hohenberger , T. de Baère , A. Le Cesne , S. Helfre , E. Saada‐Bouzid , A. Borkowska , R. Anghel , A. Co , M. Gebhart , G. Kantor , A. Montero , H. H. Loong , R. Vergés , Lancet Oncol. 2019, 20, 1148. 31296491 10.1016/S1470-2045(19)30326-2

[220] R. Li , Y. Fan , L. Liu , H. Ma , D. Gong , Z. Miao , H. Wang , Z. Zha , ACS Nano 2022, 16, 15026. 36037406 10.1021/acsnano.2c06151

[221] Y. Su , S. Liu , Y. Guan , Z. Xie , M. Zheng , X. Jing , Biomaterials 2020, 255, 120110. 32540755 10.1016/j.biomaterials.2020.120110

[222] A. Seweryn , M. Alicka , A. Fal , K. Kornicka‐Garbowska , K. Lawniczak‐Jablonska , M. Ozga , P. Kuzmiuk , M. Godlewski , K. Marycz , J. Nanobiotechnol. 2020, 18, 132. 10.1186/s12951-020-00692-5 PMC7493872 32933533

[223] E. Alphandéry , Magnetic Nanoparticles Sequentially Irradiated by Laser Radiation for Medical or Chemical or Biological or Cosmetic Applications 2019, US20190350869A1.

[224] A. Heidari , K. Schmitt , M. Henderson , E. Besana , Int. Res. J. Applied Sci. 2019, 1, 1.

[225] K. H. Thompson , J. Lichter , C. LeBel , M. C. Scaife , J. H. McNeill , C. Orvig , J. Inorg. Biochem. 2009, 103, 554. 19162329 10.1016/j.jinorgbio.2008.12.003

[226] D. Hu , D. Li , X. Liu , Z. Zhou , J. Tang , Y. Shen , Biomed. Mater. 2020, 16, 014101. 33355313 10.1088/1748-605X/abb523

[227] E. Kioseoglou , S. Petanidis , C. Gabriel , A. Salifoglou , Coord. Chem. Rev. 2015, 301–302, 87.

[228] J. M. Wörle‐Knirsch , K. Kern , C. Schleh , C. Adelhelm , C. Feldmann , H. F. Krug , Environ. Sci. Technol. 2007, 41, 331. 17265967 10.1021/es061140x

[229] M. Farahmandjou , JNMR 2017, 5, 10.15406/jnmr.2017.05.00103.

[230] P. R. Suma , R. A. Padmanabhan , S. R. Telukutla , R. Ravindran , A. K. G. Velikkakath , C. D. Dekiwadia , W. Paul , M. Laloraya , S. M. Srinivasula , S. V. Bhosale , R. S. Jayasree , Free Radic. Biol. Med. 2020, 161, 198. 33065180 10.1016/j.freeradbiomed.2020.10.008

[231] M. Naguib , J. Halim , J. Lu , K. M. Cook , L. Hultman , Y. Gogotsi , M. W. Barsoum , J. Am. Chem. Soc. 2013, 135, 15966. 24144164 10.1021/ja405735d

[232] A. VahidMohammadi , W. Liang , M. Mojtabavi , M. Wanunu , M. Beidaghi , Energy Storage Mater. 2021, 41, 554.

[233] R. Thakur , M. Hoffman , A. VahidMohammadi , J. Smith , M. Chi , B. Tatarchuk , M. Beidaghi , C. A. Carrero , ChemCatChem 2020, 12, 3639.

[234] A. Kulkarni , G. S. Kumar , J. Kaur , K. Tikoo , Inhalation Toxicol. 2014, 26, 772. 10.3109/08958378.2014.960106 25296879

[235] A. M. Jastrzębska , B. Scheibe , A. Szuplewska , A. Rozmysłowska‐Wojciechowska , M. Chudy , C. Aparicio , M. Scheibe , I. Janica , A. Ciesielski , M. Otyepka , M. W. Barsoum , Mater. Sci. Eng., C 2021, 119, 111431. 10.1016/j.msec.2020.111431 33321581

[236] S. Zada , W. Dai , Z. Kai , H. Lu , X. Meng , Y. Zhang , Y. Cheng , F. Yan , P. Fu , X. Zhang , H. Dong , Angew. Chem., Int. Ed. 2020, 59, 6601. 10.1002/anie.201916748 31994305

[237] H. Hu , H. Huang , L. Xia , X. Qian , W. Feng , Y. Chen , Y. Li , Chem. Eng. J. 2022, 440, 135810.

[238] H. Wang , X. Liu , X. Yan , J. Fan , D. Li , J. Ren , X. Qu , Chem. Sci. 2022, 13, 6704. 35756527 10.1039/d1sc07073h PMC9172572

[239] X. Han , X. Jing , D. Yang , H. Lin , Z. Wang , H. Ran , P. Li , Y. Chen , Theranostics 2018, 8, 4491. 30214634 10.7150/thno.26291 PMC6134934

[240] Y. Lu , X. Zhang , X. Hou , M. Feng , Z. Cao , J. Liu , Nanoscale 2021, 13, 17822. 34668898 10.1039/d1nr05126a

[241] C. He , L. Yu , H. Yao , Y. Chen , Y. Hao , Adv. Funct. Mater. 2021, 31, 2006214.

[242] C. Yang , Y. Luo , H. Lin , M. Ge , J. Shi , X. Zhang , ACS Nano 2021, 15, 1086. 33372766 10.1021/acsnano.0c08045

[243] W. Xiong , Y. Wu , K.‐Y. Sun , X. S. Wu , Y.‐H. Zhou , H.‐L. Cai , X. Zou , Mater. Des. 2022, 213, 110351.

[244] Z. Liu , H. Lin , M. Zhao , C. Dai , S. Zhang , W. Peng , Y. Chen , Theranostics 2018, 8, 1648. 29556347 10.7150/thno.23369 PMC5858173

[245] C. Dai , Y. Chen , X. Jing , L. Xiang , D. Yang , H. Lin , Z. Liu , X. Han , R. Wu , ACS Nano 2017, 11, 12696. 29156126 10.1021/acsnano.7b07241

[246] A. Rafieerad , A. Amiri , G. L. Sequiera , W. Yan , Y. Chen , A. A. Polycarpou , S. Dhingra , Adv. Funct. Mater. 2021, 31, 2170219. 10.1002/adfm.202100015 PMC8889894 35264918

[247] A. Rafieerad , W. Yan , K. N. Alagarsamy , A. Srivastava , N. Sareen , R. C. Arora , S. Dhingra , Adv. Funct. Mater. 2021, 31, 2106786. 35153642 10.1002/adfm.202106786 PMC8820728

[248] P. J. Havel , The Diabetes Educator 2004, Suppl, 2. 15208835

[249] S. Fakharzadeh , S. Kalanaky , H. Argani , S. Dadashzadeh , P. M. Torbati , M. H. Nazaran , A. Basiri , Drug Dev. Res. 2021, 82, 393. 33230842 10.1002/ddr.21759

[250] A. T. Hung , B. J. Leury , M. A. Sabin , F. Fahri , K. DiGiacomo , T.‐F. Lien , F. R. Dunshea , Animals 2020, 10, 1685. 32961883 10.3390/ani10091685 PMC7552722

[251] I. Papageorgiou , C. Brown , R. Schins , S. Singh , R. Newson , S. Davis , J. Fisher , E. Ingham , C. P. Case , Biomaterials 2007, 28, 2946. 17379299 10.1016/j.biomaterials.2007.02.034

[252] Q. Liu , Z. Sun , Y. Duo , L. Yin , K. Lv , L. Yue , Q.‐F. Meng , D. Nie , J. Chen , D. Luo , L.‐P. Liu , L. Rao , ACS Materials Lett. 2023, 5, 1738.

[253] J. Liu , Q. Sun , Y. Kawazoe , P. Jena , Phys. Chem. Chem. Phys. 2016, 18, 8777. 26452203 10.1039/c5cp04835d

[254] Y. Zhang , F. Li , J. Magn. Magn. Mater. 2017, 433, 222.

[255] X. Zou , H. Liu , H. Xu , X. Wu , X. Han , J. Kang , K. M. Reddy , Mater. Today Energy 2021, 20, 100668.

[256] J. Halim , S. Kota , M. R. Lukatskaya , M. Naguib , M.‐Q. Zhao , E. J. Moon , J. Pitock , J. Nanda , S. J. May , Y. Gogotsi , M. W. Barsoum , Adv. Funct. Mater. 2016, 26, 3118.

[257] Z. W. Seh , K. D. Fredrickson , B. Anasori , J. Kibsgaard , A. L. Strickler , M. R. Lukatskaya , Y. Gogotsi , T. F. Jaramillo , A. Vojvodic , ACS Energy Lett. 2016, 1, 589.

[258] W. Feng , R. Wang , Y. Zhou , L. Ding , X. Gao , B. Zhou , P. Hu , Y. Chen , Adv. Funct. Mater. 2019, 29, 1901942.

[259] S. Roy , K. A. Deo , K. A. Singh , H. P. Lee , A. Jaiswal , A. K. Gaharwar , Adv. Drug Delivery Rev. 2022, 187, 114361. 10.1016/j.addr.2022.114361 PMC12861116 35636569

[260] D. Sahoo , S. P. Behera , J. Shakya , B. Kaviraj , PLoS One 2022, 17, e0260955. 35041665 10.1371/journal.pone.0260955 PMC8765608

[261] J. Lu , M. Chen , L. Dong , L. Cai , M. Zhao , Q. Wang , J. Li , Colloids Surf., B 2020, 194, 111162. 10.1016/j.colsurfb.2020.111162 32512311

[262] T. Liu , C. Wang , X. Gu , H. Gong , L. Cheng , X. Shi , L. Feng , B. Sun , Z. Liu , Adv. Mater. 2014, 26, 3433. 24677423 10.1002/adma.201305256

[263] J. Hao , G. Song , T. Liu , X. Yi , K. Yang , L. Cheng , Z. Liu , Adv. Sci. 2017, 4, 1600160. 10.1002/advs.201600160 PMC5238746 28105392

[264] R. Kurapati , L. Muzi , A. P. R. de Garibay , J. Russier , D. Voiry , I. A. Vacchi , M. Chhowalla , A. Bianco , Adv. Funct. Mater. 2017, 27, 1605176.

[265] J. Yu , W. Yin , X. Zheng , G. Tian , X. Zhang , T. Bao , X. Dong , Z. Wang , Z. Gu , X. Ma , Y. Zhao , Theranostics 2015, 5, 931. 26155310 10.7150/thno.11802 PMC4493532

[266] B. Liu , C. Li , G. Chen , B. Liu , X. Deng , Y. Wei , J. Xia , B. Xing , P. Ma , J. Lin , Adv. Sci. 2017, 4, 1600540. 10.1002/advs.201600540 PMC5566229 28852616

[267] X. Ren , D. Wu , R. Ge , X. Sun , H. Ma , T. Yan , Y. Zhang , B. Du , Q. Wei , L. Chen , Nano Res. 2018, 11, 2024.

[268] S. Wang , X. Li , Y. Chen , X. Cai , H. Yao , W. Gao , Y. Zheng , X. An , J. Shi , H. Chen , Adv. Mater. 2015, 27, 2775. 25821185 10.1002/adma.201500870

[269] C. Wu , J. Zhao , F. Hu , Y. Zheng , H. Yang , S. Pan , S. Shi , X. Chen , S. Wang , Carbohydr. Polym. 2018, 180, 112. 29103486 10.1016/j.carbpol.2017.10.024

[270] T. Liu , Y. Chao , M. Gao , C. Liang , Q. Chen , G. Song , L. Cheng , Z. Liu , Nano Res. 2016, 9, 3003.

[271] J. Wang , X. Tan , X. Pang , L. Liu , F. Tan , N. Li , ACS Appl. Mater. Interfaces 2016, 8, 24331. 27595856 10.1021/acsami.6b08391

[272] W. Yin , L. Yan , J. Yu , G. Tian , L. Zhou , X. Zheng , X. Zhang , Y. Yong , J. Li , Z. Gu , Y. Zhao , ACS Nano 2014, 8, 6922. 24905027 10.1021/nn501647j

[273] S. K. Maji , S. Yu , K. Chung , M. Sekkarapatti Ramasamy , J. W. Lim , J. Wang , H. Lee , D. H. Kim , ACS Appl. Mater. Interfaces 2018, 10, 42068. 30462488 10.1021/acsami.8b15443

[274] C. Moore , A. Harvey , J. N. Coleman , H. J. Byrne , J. McIntyre , 2D Mater. 2020, 7, 025003.

[275] Z. Gu , S. H. Chen , Z. Ding , W. Song , W. Wei , S. Liu , G. Ma , R. Zhou , Nanoscale 2019, 11, 22293. 31746904 10.1039/c9nr04358f

[276] G. Sun , S. Yang , H. Cai , Y. Shu , Q. Han , B. Wang , Z. Li , L. Zhou , Q. Gao , Z. Yin , J. Colloid Interface Sci. 2019, 549, 50. 31015056 10.1016/j.jcis.2019.04.047

[277] C. Song , Z. Li , Y. Chen , C. Zheng , N. Hu , C. Guo , New J. Chem. 2019, 43, 1838.

[278] L. Deng , X. Pan , Y. Zhang , S. Sun , L. Lv , L. Gao , P. Ma , H. Ai , Q. Zhou , X. Wang , L. Zhan , Int. J. Nanomedicine 2020, 15, 2971. 32431496 10.2147/IJN.S243537 PMC7197944

[279] Q. Han , X. Wang , X. Jia , S. Cai , W. Liang , Y. Qin , R. Yang , C. Wang , Nanoscale 2017, 9, 5927. 28436514 10.1039/c7nr01460k

[280] D. Baimanov , J. Wu , R. Chu , R. Cai , B. Wang , M. Cao , Y. Tao , J. Liu , M. Guo , J. Wang , X. Yuan , C. Ji , Y. Zhao , W. Feng , L. Wang , C. Chen , ACS Nano 2020, 14, 5529. 32283010 10.1021/acsnano.9b09744

[281] F. Jiang , B. Ding , S. Liang , Y. Zhao , Z. Cheng , B. Xing , P. Ma , J. Lin , Biomaterials 2021, 268, 120545. 33253965 10.1016/j.biomaterials.2020.120545

[282] T. Zheng , W. Sang , Z. He , Q. Wei , B. Chen , H. Li , C. Cao , R. Huang , X. Yan , B. Pan , S. Zhou , J. Zeng , Nano Lett. 2017, 17, 7968. 29178807 10.1021/acs.nanolett.7b04430

[283] H. Liang , H. Xi , S. Liu , X. Zhang , H. Liu , Nanoscale 2019, 11, 18183. 31556902 10.1039/c9nr06222j

[284] L. Zhang , S. Zhao , J. Ouyang , L. Deng , Y.‐N. Liu , Chem. Eng. J. 2022, 431, 133273.

[285] B. Zheng , Y. Bai , H. Chen , H. Pan , W. Ji , X. Gong , X. Wu , H. Wang , J. Chang , Biomater. Sci. 2018, 6, 1379. 29652059 10.1039/c8bm00218e

[286] Z. Liu , X. Liu , X. Ran , E. Ju , J. Ren , X. Qu , Biomaterials 2015, 69, 56. 26280950 10.1016/j.biomaterials.2015.08.008

[287] J. Wang , C. Zhang , W. Zhang , W. Liu , Y. Guo , P. Dang , L. Wei , H. Zhao , X. Deng , S. Guo , L. Chen , Adv. Funct. Materials 2024, 34, 2306319.

[288] A. Sharma , A. K. Saini , N. Kumar , N. Tejwan , T.h. A. Singh , V. K. Thakur , J. Das , J. Surf. Interfac. 2022, 28, 101641.

[289] Q. Xiang , C. Yang , Y. Luo , F. Liu , J. Zheng , W. Liu , H. Ran , Y. Sun , J. Ren , Z. Wang , Small 2022, 18, 2107809. 10.1002/smll.202107809 35143709

[290] X. Wang , R. Xu , X. Sun , Y. Wang , X. Ren , B. Du , D. Wu , Q. Wei , Biosens. Bioelectron. 2017, 96, 239. 28500948 10.1016/j.bios.2017.04.052

[291] A. M. Yassin , M. Elnouby , N. M. El‐Deeb , E. E. Hafez , Appl. Biochem. Biotechnol. 2016, 180, 623. 27193257 10.1007/s12010-016-2120-x

[292] D. Sandil , S. Srivastava , R. Khatri , K. Sharma , N. K. Puri , Sens. Bio‐Sens. Res. 2021, 32, 100423.

[293] Z. Zhou , T. Wang , T. Hu , H. Xu , L. Cui , B. Xue , X. Zhao , X. Pan , S. Yu , H. Li , Y. Qin , J. Zhang , L. Ma , R. Liang , C. Tan , Adv. Mater. 2024, 36, 2311002. 10.1002/adma.202311002 38408758

[294] W. Ortiz , C. Malca , D. Barrionuevo , A. Aldalbahi , E. Pacheco , N. Oli , P. Feng , Vacuum 2022, 201, 111092.

[295] M. Shorie , V. Kumar , H. Kaur , K. Singh , V. K. Tomer , P. Sabherwal , Microchim. Acta 2018, 185, 158. 10.1007/s00604-018-2705-x 29594650

[296] S. Joh , H.‐K. Na , J. G. Son , A. Y. Lee , C.‐H. Ahn , D.‐J. Ji , J.‐S. Wi , M. S. Jeong , S.‐G. Lee , T. G. Lee , ACS Nano 2021, 15, 10141. 34097394 10.1021/acsnano.1c02016

[297] K.‐J. Huang , Y.‐J. Liu , J.‐T. Cao , H.‐B. Wang , RSC Adv. 2014, 4, 36742.

[298] T. Yu , Y. Wang , K. Yuan , Q. Guo , J. Ge , Sens. Actuators, B 2022, 367, 132056.

[299] S. Niazi , I. M. Khan , Y. Yu , I. Pasha , M. Shoaib , A. Mohsin , B. S. Mushtaq , W. Akhtar , Z. Wang , Microchim. Acta 2019, 186, 575. 10.1007/s00604-019-3570-y 31342182

[300] X. Liu , G. Duan , W. Li , Z. Zhou , R. Zhou , RSC Adv. 2017, 7, 37873.

[301] Y. Yong , X. Cheng , T. Bao , M. Zu , L. Yan , W. Yin , C. Ge , D. Wang , Z. Gu , Y. Zhao , ACS Nano 2015, 9, 12451. 26495962 10.1021/acsnano.5b05825

[302] E. B. Goldman , A. Zak , R. Tenne , E. Kartvelishvily , S. Levin‐Zaidman , Y. Neumann , R. Stiubea‐Cohen , A. Palmon , A.‐H. Hovav , D. J. Aframian , Tissue Eng., Part A 2015, 21, 1013. 25366879 10.1089/ten.tea.2014.0163 PMC4356479

[303] Y.‐X. Chen , W.‐J. Zhang , K.‐J. Huang , M. Zheng , Y.‐C. Mao , Analyst 2017, 142, 4843. 29160869 10.1039/c7an01244f

[304] Y. Huang , Y. Zhao , Y. Liu , R. Ye , L. Chen , G. Bai , S. Xu , Chem. Eng. J. 2021, 411, 128610.

[305] L. Hou , C. Tian , Y. Yan , L. Zhang , H. Zhang , Z. Zhang , ACS Nano 2020, 14, 3927. 32298077 10.1021/acsnano.9b06111

[306] M. Lv , M. Chen , R. Zhang , W. Zhang , C. Wang , Y. Zhang , X. Wei , Y. Guan , J. Liu , K. Feng , M. Jing , X. Wang , Y.‐C. Liu , Q. Mei , W. Han , Z. Jiang , Cell Res. 2020, 30, 966. 32839553 10.1038/s41422-020-00395-4 PMC7785004

[307] S. Kim , S. M. Ahn , J.‐S. Lee , T. S. Kim , D.‐H. Min , 2D Mater. 2017, 4, 025069.

[308] Y. Hao , L. Wang , B. Zhang , D. Li , D. Meng , J. Shi , H. Zhang , Z. Zhang , Y. Zhang , Int. J. Nanomedicine 2016, 11, 1759. 27199556 10.2147/IJN.S98832 PMC4857809

[309] S.‐J. Zheng , M. Yang , J.‐Q. Luo , R. Liu , J. Song , Y. Chen , J.‐Z. Du , ACS Nano 2023, 17, 15905. 37565626 10.1021/acsnano.3c03962

[310] Z. Cui , T. Hu , S. Yang , Y. Yang , X. Liu , T. Wang , H. Chen , C. Zeng , R. Liang , Y. Zhou , Chem. Eng. J. 2024, 480, 147463.

[311] Z.‐H. Miao , L.‐X. Lv , K. Li , P.‐Y. Liu , Z. Li , H. Yang , Q. Zhao , M. Chang , L. Zhen , C.‐Y. Xu , Small 2018, 14, 1703789. 10.1002/smll.201703789 29468828

[312] Y. Yong , L. Zhou , Z. Gu , L. Yan , G. Tian , X. Zheng , X. Liu , X. Zhang , J. Shi , W. Cong , W. Yin , Y. Zhao , Nanoscale 2014, 6, 10394. 25047651 10.1039/c4nr02453b

[313] Y. Chen , L. Cheng , Z. Dong , Y. Chao , H. Lei , H. Zhao , J. Wang , Z. Liu , Angew. Chem. 2017, 129, 13171. 10.1002/anie.201707128 28815905

[314] A. Zaheer , S. Afsheen Zahra , M. Z. Iqbal , A. Mahmood , S. Ayaz Khan , S. Rizwan , RSC Adv. 2022, 12, 4624. 35425492 10.1039/d2ra00014h PMC8981252

[315] P. Collery , D. Desmaele , V. Vijaykumar , Curr. Pharm. Des. 2019, 25, 3306. 31475892 10.2174/1381612825666190902161400

[316] T. Wang , Q. Bai , Z. Zhu , H. Xiao , F. Jiang , F. Du , W. W. Yu , M. Liu , N. Sui , Chem. Eng. J. 2021, 413, 127537.

[317] Y. Gao , S. Liu , Y. Huang , F. Li , Y. Zhang , Front. Immunol. 2024, 15, 1379365. 38915413 10.3389/fimmu.2024.1379365 PMC11194341

[318] W. Fang , Z. Yu , P. Hu , J. Shi , Adv. Funct. Materials 2024, 2405483.

[319] M. Adeel , S. Parisi , M. Mauceri , K. Asif , M. Bartoletti , F. Puglisi , I. Caligiuri , M.d. M. Rahman , V. Canzonieri , F. Rizzolio , ACS Omega 2021, 6, 28611. 34746556 10.1021/acsomega.1c03010 PMC8567285

[320] L. Wang , K. Kang , H. Hou , Y. Ma , K. Yu , F. Qu , H. Lin , J. Colloid Interface Sci. 2022, 625, 145. 35716610 10.1016/j.jcis.2022.06.031

[321] W. Guo , H. Li , M. Li , W. Dai , Z. Shao , X. Wu , B. Yang , Carbon 2014, 79, 636.

[322] S. Tang , M. Chen , N. Zheng , Small 2014, 10, 3139. 24729448 10.1002/smll.201303631

[323] Y.‐W. Jiang , G. Gao , P. Hu , J.‐B. Liu , Y. Guo , X. Zhang , X.‐W. Yu , F.‐G. Wu , X. Lu , Nanoscale 2020, 12, 210. 31815993 10.1039/c9nr08454a

[324] Z. Wang , A. von dem Bussche , P. K. Kabadi , A. B. Kane , R. H. Hurt , ACS Nano 2013, 7, 8715. 24032665 10.1021/nn403080y PMC3894052

[325] L. Banci , I. Bertini , F. Cantini , S. Ciofi‐Baffoni , Cell. Mol. Life Sci. 2010, 67, 2563. 20333435 10.1007/s00018-010-0330-x PMC11115773

[326] G. Zhu , M. Wang , L. Qiao , Y. Xie , J. Wang , L. Li , Q. Sun , P. Zheng , C. Li , Adv. Funct. Materials 2024, 34, 2400496.

[327] J. Liu , L. Li , R. Zhang , Z. P. Xu , Nanoscale Horiz. 2023, 8, 279. 36606452 10.1039/d2nh00478j

[328] W. Tang , J. Wu , L. Wang , K. Wei , Z. Pei , F. Gong , L. Chen , Z. Han , Y. Yang , Y. Dai , X. Cui , L. Cheng , ACS Nano 2024, 18, 10495. 38556991 10.1021/acsnano.3c11818

[329] Y. Yang , T. Hu , Y. Bian , F. Meng , S. Yu , H. Li , Q. Zhang , L. Gu , X. Weng , C. Tan , R. Liang , Adv. Mater. 2023, 35, 2211205. 10.1002/adma.202211205 36913539

[330] S. Li , Q. Zhu , Y. Sun , L. Wang , J. Lu , Q. Nie , Y. Ma , W. Jing , Ind. Eng. Chem. Res. 2020, 59, 7797.

[331] H. A. Ghramh , E. H. Ibrahim , M. Kilany , Food Sci. Nutr. 2020, 8, 445. 31993170 10.1002/fsn3.1328 PMC6977415

[332] D. Morris , M. Ansar , J. Speshock , T. Ivanciuc , Y. Qu , A. Casola , R. P. Garofalo , Viruses 2019, 11, 732. 31398832 10.3390/v11080732 PMC6723559

[333] J. Bae , M. Ha , H. Perumalsamy , Y. Lee , J. Song , T.‐H. Yoon , Pharmaceutics 2022, 14, 630. 35336005 10.3390/pharmaceutics14030630 PMC8954471

[334] R.‐J. Chen , C.‐C. Huang , R. Pranata , Y.‐H. Lee , Y.‐Y. Chen , Y.‐H. Wu , Y.‐J. Wang , Int. J. Mol. Sci. 2021, 22, 2536.33802568

[335] X. Zhao , H. Tang , X. Jiang , ACS Nano 2022, 16, 10066. 35776694 10.1021/acsnano.2c02269

[336] M. S. Shakil , M. S. Niloy , K. M. Mahmud , M. A. Kamal , M. A. Islam , Cancers 2022, 14, 3047. 35804818 10.3390/cancers14133047 PMC9264814

[337] Z. Zhou , D. Li , X. Fan , Y. Yuan , H. Wang , D. Wang , X. Mei , Regen. Biomater. 2022, 9, rbab072. 35558096 10.1093/rb/rbab072 PMC9089162

[338] Ž. Krpetić , F. Porta , E. Caneva , V. Dal Santo , G. Scarì , Langmuir 2010, 26, 14799. 20795674 10.1021/la102758f

[339] S. Tardito , G. Martinelli , S. Soldano , S. Paolino , G. Pacini , M. Patane , E. Alessandri , V. Smith , M. Cutolo , Autoimmun Rev. 2019, 18, 102397. 31520798 10.1016/j.autrev.2019.102397

[340] M. A. Khan , M. J. Khan , Artif. Cells, Nanomed., Biotechnol. 2018, 46, 1149. 29553845 10.1080/21691401.2018.1446968

[341] T. Beduk , D. Beduk , J. I. de Oliveira Filho , F. Zihnioglu , C. Cicek , R. Sertoz , B. Arda , T. Goksel , K. Turhan , K. N. Salama , S. Timur , Anal. Chem. 2021, 93, 8585. 34081452 10.1021/acs.analchem.1c01444

[342] W. Zhu , Y. Yang , Q. Jin , Y. Chao , L. Tian , J. Liu , Z. Dong , Z. Liu , Nano Res. 2019, 12, 1307.

[343] A. Moorthy , M. Raj Subramaniam , T. G. Manivasagam , D. Kumaresan , Dalton Trans. 2018, 47, 8683. 29901672 10.1039/c8dt00638e

[344] B. Jin , T. Zhai , Chem. Res. Chin. Univ. 2020, 36, 493.

[345] P. Patra , R. Kumar , P. K. Mahato , C. Bhakat , C. Kumar , Mater. Today: Proc. 2022, 56, 811.

[346] Y. Su , F. Peng , Z. Jiang , Y. Zhong , Y. Lu , X. Jiang , Q. Huang , C. Fan , S.‐T. Lee , Y. He , Biomaterials 2011, 32, 5855. 21601920 10.1016/j.biomaterials.2011.04.063

[347] X. Li , H. Wei , Y. Hu , Y. Lv , L. Weng , Z. Teng , L. Yuwen , L. Wang , J. Appl. Toxicol. 2022, 42, 1757. 35618442 10.1002/jat.4352

[348] K. Golovine , P. Makhov , R. G. Uzzo , A. Kutikov , D. J. Kaplan , E. Fox , V. M. Kolenko , Mol. Cancer 2010, 9, 183. 20618956 10.1186/1476-4598-9-183 PMC3044330

[349] J. M. S. Al‐Murshdy , H. H. J. Al‐Deen , S. R. Hussein , J. Bio. Tribo. Corros. 2021, 7, 148.

[350] D. Singh , S. K. Gupta , I. Lukačević , Y. Sonvane , RSC Adv. 2016, 6, 8006.

[351] C. Grazianetti , C. Martella , Materials 2021, 14, 4170. 34361369 10.3390/ma14154170 PMC8347995

[352] S. Yadav , M. Abubakar Sadique , A. Kaushik , P. Ranjan , R. Khan , A. K. Srivastava , J. Mater. Chem. B 2022, 10, 1146. 36197135 10.1039/d2tb01409b

[353] M. Han , L. Zhu , J. Mo , W. Wei , B. Yuan , J. Zhao , C. Cao , ACS Appl. Bio Mater. 2020, 3, 4220. 10.1021/acsabm.0c00306 35025423

[354] E. Shakerzadeh , J. Mol. Liq. 2017, 240, 682.

[355] P. Marrack , A. S. McKee , M. W. Munks , Nat. Rev. Immunol. 2009, 9, 287. 19247370 10.1038/nri2510 PMC3147301

[356] L. C. Becker , I. Boyer , W. F. Bergfeld , D. V. Belsito , R. A. Hill , C. D. Klaassen , D. C. Liebler , J. G. Marks , R. C. Shank , T. J. Slaga , P. W. Snyder , F. A. Andersen , Int. J. Toxicol. 2016, 35, 16S. 27913785 10.1177/1091581816677948

[357] A. S. Lozhkomoev , G. Mikhaylov , V. Turk , B. Turk , O. Vasiljeva , *Multiscale Biomechanics and Tribology of Inorganic and Organic Systems* *. in* Memory of Professor SergeyPsakhie (Eds.: G.‐P. Ostermeyer , V. L. Popov , E. V. Shilko , O. S. Vasiljeva ), Springer International Publishing, Cham, 2021, pp. 211–223.

[358] H. Vrieling , S. Kooijman , J. W. De Ridder , D. M. E. Thies‐Weesie , P. C. Soema , W. Jiskoot , E. Van Riet , A. J. R. Heck , A. P. Philipse , G. F. A. Kersten , H. D. Meiring , J. L. Pennings , B. Metz , J. Pharm. Sci. 2020, 109, 750. 31449816 10.1016/j.xphs.2019.08.014

[359] C. Seidl , S. Simonato , E. Zittel , U. Schepers , C. Feldmann , Zeitschrift anorg allge chemie 2019, 645, 1372.

[360] M. I. Lerner , G. Mikhaylov , A. A. Tsukanov , A. S. Lozhkomoev , E. Gutmanas , I. Gotman , A. Bratovs , V. Turk , B. Turk , S. G. Psakhye , O. Vasiljeva , Nano Lett. 2018, 18, 5401. 30070485 10.1021/acs.nanolett.8b01592

[361] V. Kochat , A. Samanta , Y. Zhang , S. Bhowmick , P. Manimunda , S. A. S. Asif , A. S. Stender , R. Vajtai , A. K. Singh , C. S. Tiwary , P. M. Ajayan , Sci. Adv. 2018, 4, e1701373. 29536039 10.1126/sciadv.1701373 PMC5844710

[362] W. Tao , N. Kong , X. Ji , Y. Zhang , A. Sharma , J. Ouyang , B. Qi , J. Wang , N. Xie , C. Kang , H. Zhang , O. C. Farokhzad , J. S. Kim , Chem. Soc. Rev. 2019, 48, 2891. 31120049 10.1039/c8cs00823j

[363] Y. Yang , M. Li , B. Zhou , X. Jiang , D. Zhang , H. Luo , Bioactive Materials 2023, 25, 594. 37056253 10.1016/j.bioactmat.2022.07.015 PMC10087081

[364] S. Cheeseman , S. J. Bryant , L. Z. Y. Huang , E. L. H. Mayes , R. J. Crawford , T. Daeneke , J. Chapman , V. K. Truong , A. Elbourne , ACS Appl. Nano Mater. 2022, 5, 16584.

[365] C.‐C. Qu , Y.‐T. Liang , X.‐Q. Wang , S. Gao , Z.‐Z. He , X.‐Y. Sun , Bioengineering 2022, 9, 416.36134962

[366] N. Yang , N. Shi , Z. Yao , H. Liu , W. Guo , Front. Bioeng. Biotechnol. 2023, 11. 10.3389/fbioe.2023.1124944 PMC9908762 36777248

[367] M. Kurtjak , M. Vukomanović , L. Kramer , D. Suvorov , J. Mater. Sci.: Mater. Med. 2016, 27, 170. 27704374 10.1007/s10856-016-5777-3

[368] “Sub 150 nm Nanoscale Gallium Based Metal–Organic Frameworks Armored Antibiotics as Super Penetrating Bombs for Eradicating Persistent Bacteria – Huang –2022 – Advanced Functional Materials – Wiley Online Library,” can be found under https://onlinelibrary‐wiley‐com.uml.idm.oclc.org/doi/full/10.1002/adfm.202204906, n.d.

[369] C. Zhang , B. Yang , J. M. Biazik , R. F. Webster , W. Xie , J. Tang , F.‐M. Allioux , R. Abbasi , M. Mousavi , E. M. Goldys , K. A. Kilian , R. Chandrawati , D. Esrafilzadeh , K. Kalantar‐Zadeh , ACS Nano 2022, 16, 8891. 35613428 10.1021/acsnano.1c10981

[370] T. Teerasarntipan , R. Chaiteerakij , P. Prueksapanich , D. Werawatganon , BMC Gastroenterol. 2020, 20, 263. 32770948 10.1186/s12876-020-01386-w PMC7414709

[371] N. N. Dongre , A. N. Suryakar , A. J. Patil , D. B. Rathi , 2010, 3.

[372] “New Breast Cancer Study Findings Reported from Cardiff University (Structural modifications on CORM‐3 lead to enhanced anti‐angiogenic properties against triple‐negative breast cancer cells). – Document – Gale OneFile: Health and Medicine,” can be found under https://go-gale-com.uml.idm.oclc.org/ps/i.do?p=HRCA&u=univmanitoba&id=GALE|A608737004&v=2.1&it=r, n.d. 10.2174/1573406415666191206102452 31808392

[373] Z. Chen , Q. Lu , X. Cao , K. Wang , Y. Wang , Y. Wu , Z. Yang , Ecotoxicol. Environ. Saf. 2022, 247, 114204. 36274319 10.1016/j.ecoenv.2022.114204

[374] P. Rivero , J.‐A. Yan , V. M. García‐Suárez , J. Ferrer , S. Barraza‐Lopez , Phys. Rev. B 2014, 90, 241408.

[375] X. Cui , S. Xu , X. Wang , C. Chen , Carbon 2018, 138, 436.

[376] Y. Tu , M. Lv , P. Xiu , T. Huynh , M. Zhang , M. Castelli , Z. Liu , Q. Huang , C. Fan , H. Fang , R. Zhou , Nature Nanotech. 2013, 8, 594. 10.1038/nnano.2013.125 23832191

[377] Z. Du , C. Wang , R. Zhang , X. Wang , X. Li , Int. J. Nanomedicine 2020, 15, 7523. 33116486 10.2147/IJN.S271917 PMC7547809

[378] D. Xue , E. Chen , H. Zhong , W. Zhang , S. Wang , M. U. Joomun , T. Yao , Y. Tan , S. Lin , Q. Zheng , Z. Pan , Int. J. Nanomedicine 2018, 13, 5799. 30310282 10.2147/IJN.S170305 PMC6165768

[379] V. Bordoni , G. Reina , M. Orecchioni , G. Furesi , S. Thiele , C. Gardin , B. Zavan , G. Cuniberti , A. Bianco , M. Rauner , L. G. Delogu , Nanoscale 2019, 11, 19408. 31386739 10.1039/c9nr03975a

[380] S. F. Oliveira , G. Bisker , N. A. Bakh , S. L. Gibbs , M. P. Landry , M. S. Strano , Carbon 2015, 95, 767.

[381] S. Malanagahalli , D. Murera , C. Martín , H. Lin , N. Wadier , H. Dumortier , E. Vázquez , A. Bianco , Nanomaterials 2020, 10, 228. 32013038 10.3390/nano10020228 PMC7074970

[382] T. Fulop , A. Larbi , G. Dupuis , A. Le Page , E. H. Frost , A. A. Cohen , J. M. Witkowski , C. Franceschi , Frontiers in Immunology 2018, 8. 10.3389/fimmu.2017.01960 PMC5767595 29375577

[383] R. Huq , E. L. G. Samuel , W. K. A. Sikkema , L. G. Nilewski , T. Lee , M. R. Tanner , F. S. Khan , P. C. Porter , R. B. Tajhya , R. S. Patel , T. Inoue , R. G. Pautler , D. B. Corry , J. M. Tour , C. Beeton , Sci. Rep. 2016, 6, 33808. 27654170 10.1038/srep33808 PMC5031970

[384] F. Ayaz , M. O. Alas , R. Genc , Inflammation 2020, 43, 777. 31873835 10.1007/s10753-019-01165-0

[385] M. Orecchioni , D. Bedognetti , F. Sgarrella , F. M. Marincola , A. Bianco , L. G. Delogu , J. Transl. Med. 2014, 12, 138. 24885781 10.1186/1479-5876-12-138 PMC4067374

[386] B. Luan , T. Huynh , L. Zhao , R. Zhou , ACS Nano 2015, 9, 663. 25494677 10.1021/nn506011j

[387] B. Wang , X. Su , J. Liang , L. Yang , Q. Hu , X. Shan , J. Wan , Z. Hu , Mater. Sci. Eng., C 2018, 90, 514. 10.1016/j.msec.2018.04.096 29853120

[388] G. Choe , S.‐W. Kim , J. Park , J. Park , S. Kim , Y. S. Kim , Y. Ahn , D.‐W. Jung , D. R. Williams , J. Y. Lee , Biomaterials 2019, 225, 119513. 31569016 10.1016/j.biomaterials.2019.119513

[389] K. Pondman , C. Savador‐Morales , B. Paudyal , R. Sim , U. Kishore , Nanoscale Horiz. 2017, *Available on‐line*. 10.1039/c6nh00227g 32260639

[390] S. Zhou , Y. Hashida , S. Kawakami , J. Mihara , T. Umeyama , H. Imahori , T. Murakami , F. Yamashita , M. Hashida , Int. J. Pharm. 2014, 471, 214. 24861942 10.1016/j.ijpharm.2014.05.037

[391] X. Cao , M. Zhen , L. Li , Z. Wu , C. Zhou , J. Huo , S. Su , Y. Xu , W. Jia , X. Liao , Z. Sun , H. Li , C. Wang , J. Mater. Chem. B 2022, 10, 9457. 36346268 10.1039/d2tb01518h

[392] C. Fei , L. Liu , H. Qi , Y. Peng , J. Han , C. Wang , X. Li , ACS Appl. Mater. Interfaces 2024, 16, 5536. 38267397 10.1021/acsami.3c16168 PMC10860698

[393] M. Jaroniec , Nature Chem. 2009, 1, 166. 21378833 10.1038/nchem.173

[394] B. Hidding , Science 2018, 360, 489. 29724940 10.1126/science.aap8005

[395] Y. Huang , P. Li , R. Zhao , L. Zhao , J. Liu , S. Peng , X. Fu , X. Wang , R. Luo , R. Wang , Z. Zhang , Biomed. Pharmacother. 2022, 151, 113053. 35594717 10.1016/j.biopha.2022.113053

[396] T.‐H. T. Nguyen , N.‐T. Trinh , H. N. Tran , H. T. Tran , P. Q. Le , D.‐N. Ngo , H. Tran‐Van , T. Van Vo , L. B. Vong , Y. Nagasaki , J. Controlled Release 2021, 331, 515. 10.1016/j.jconrel.2020.10.042 33616078

[397] C. J. Tsao , L. Pandolfi , X. Wang , S. Minardi , C. Lupo , M. Evangelopoulos , T. Hendrickson , A. Shi , G. Storci , F. Taraballi , E. Tasciotti , ACS Appl. Mater. Interfaces 2018, 10, 44344. 30511828 10.1021/acsami.8b19975

[398] Y. Yuan , Q. Guo , X. Zhang , W. Jiang , C. Ye , X. Zhou , J. Mater. Chem. B 2020, 8, 5014. 32301463 10.1039/d0tb00484g

[399] F. L. Portilho , E. Helal‐Neto , S. S. Cabezas , S. R. Pinto , S. N. dos Santos , L. Pozzo , F. Sancenón , R. Martínez‐Máñez , R. Santos‐Oliveira , Artif. Cells, Nanomed., Biotechnol. 2018, 46, 1080. 29482360 10.1080/21691401.2018.1443941

[400] F. Chen , M. Ma , J. Wang , F. Wang , S.‐X. Chern , E. R. Zhao , A. Jhunjhunwala , S. Darmadi , H. Chen , J. V. Jokerst , Nanoscale 2016, 9, 402. 27924340 10.1039/c6nr08177k PMC5179289

[401] H. Lin , W. Qiu , J. Liu , L. Yu , S. Gao , H. Yao , Y. Chen , J. Shi , Adv. Mater. 2019, 31, 1903013. 10.1002/adma.201903013 31347215

[402] Y. You , C. Yang , X. Zhang , H. Lin , J. Shi , Materials Today Nano 2021, 16, 100132.

[403] N. Ni , X. Zhang , Y. Ma , J. Yuan , D. Wang , G. Ma , J. Dong , X. Sun , Coord. Chem. Rev. 2022, 458, 214415.

[404] R. Hu , Z. Chen , C. Dai , X. Guo , W. Feng , Z. Liu , H. Lin , Y. Chen , R. Wu , Biomaterials 2021, 269, 120455. 33162174 10.1016/j.biomaterials.2020.120455

[405] F. Wang , H. Duan , R. Zhang , H. Guo , H. Lin , Y. Chen , Nanoscale 2020, 12, 17931. 32845945 10.1039/d0nr05214k

[406] B. Ding , X. Wang , J. Yu , Electrospinning: Nanofabrication and Applications, William Andrew, Norwich, New York, 2018.

[407] D. Xu , H. Lin , W. Qiu , M. Ge , Z. Chen , C. Wu , Y. You , X. Lu , C. Wei , J. Liu , X. Guo , J. Shi , Biomaterials 2021, 278, 121172. 34653935 10.1016/j.biomaterials.2021.121172

[408] C. He , L. Yu , L. Ding , H. Yao , Y. Chen , Y. Hao , Biomaterials 2020, 255, 120181. 32569864 10.1016/j.biomaterials.2020.120181

[409] Y. You , Y.‐X. Zhu , J. Jiang , M. Wang , Z. Chen , C. Wu , J. Wang , W. Qiu , D. Xu , H. Lin , J. Shi , J. Am. Chem. Soc. 2022, 144, 14195. 35830228 10.1021/jacs.2c04412

[410] Z. Lin , Z. Chen , Y. Chen , N. Yang , J. Shi , Z. Tang , C. Zhang , H. Lin , J. Yin , Exploration 2023, 3, 20220149. 37933236 10.1002/EXP.20220149 PMC10624372

[411] C. D. Seaborn , F. H. Nielsen , Biol. Trace Elem. Res. 1994, 42, 151. 7981005 10.1007/BF02785386

[412] H. M. Dhingra , T. Umsawasdi , D. F. Chiuten , W. K. Murphy , P. Y. Holoye , G. Spitzer , M. Valdivieso , Cancer Treat Rep. 1986, 70, 673. 3708617

[413] B. Kamen , n.d.

[414] M. Jung , M.‐K. Shin , S.‐B. Cha , S. W. Shin , A. Yoo , W.‐J. Lee , H.‐T. Park , J.‐H. Park , B. Kim , Y.‐K. Jung , H. S. Yoo , BMC Vet. Res. 2014, 10, 179. 25255918 10.1186/s12917-014-0179-6 PMC4236827

[415] D. Dobrzyński , A. Boguszewska‐Czubara , K. Sugimori , Environ. Geochem. Health 2018, 40, 1355. 29299858 10.1007/s10653-017-0061-0 PMC6061135

[416] L. Li , G. Xu , H. Shao , Z.‐H. Zhang , X.‐F. Pan , J.‐Y. Li , Int. J. Environ. Res. Public Health 2017, 14, 227. 28245579 10.3390/ijerph14030227 PMC5369063

[417] J. M. Cho , J. Chae , S. R. Jeong , M. J. Moon , D. Y. Shin , J. H. Lee , PLoS One 2020, 15, e0240358. 33075061 10.1371/journal.pone.0240358 PMC7572073

[418] Y. Wang , G. Teng , H. Zhou , C. Dong , Biol. Trace Elem. Res. 2020, 198, 617. 32144718 10.1007/s12011-020-02106-x

[419] K. Marino , R. Lee , P. Lee , Orthop. J. Sports Med. 2019, 7, 2325967119879124. 31696136 10.1177/2325967119879124 PMC6820190

[420] S. Balendhran , S. Walia , H. Nili , S. Sriram , M. Bhaskaran , Small 2015, 11, 640. 25380184 10.1002/smll.201402041

[421] M. Ge , M. Zong , D. Xu , Z. Chen , J. Yang , H. Yao , C. Wei , Y. Chen , H. Lin , J. Shi , Mater. Today Nano 2021, 15, 100119.

[422] M. Fojtů , J. Balvan , M. Raudenská , T. Vičar , J. Šturala , Z. Sofer , J. Luxa , J. Plutnar , M. Masařík , M. Pumera , Appl. Mater. Today 2020, 20, 100697.

[423] Z. Chen , F. Qi , W. Qiu , C. Wu , M. Zong , M. Ge , D. Xu , Y. You , Y.‐X. Zhu , Z. Zhang , H. Lin , J. Shi , Adv. Sci. 2022, 9, 2202933. 10.1002/advs.202202933 PMC9685437 36202760

[424] A. Ahmeda , B. Mahdavi , F. Zaker , S. Kaviani , S. Hosseini , M. M. Zangeneh , A. Zangeneh , S. Paydarfar , R. Moradi , Appl. Organomet. Chem. 2020, 34, e5433.

[425] R. Sukanya , S. Ramki , S.‐M. Chen , R. Karthik , Anal. Chim. Acta 2020, 1096, 76. 31883594 10.1016/j.aca.2019.10.059

[426] Z. Tian , A. Chinnathambi , T. Awad Alahmadi , S. Krishna Mohan , V. Priya Veeraraghavan , S. Kumar Jaganathan , Arabian J. Chem. 2021, 14, 103293.

[427] A. Naji , B. A. Muzembo , K. Yagyu , N. Baba , F. Deschaseaux , L. Sensebé , N. Suganuma , Sci. Rep. 2016, 6, 26162. 27194621 10.1038/srep26162 PMC4872131

[428] N. Liu , Y. Guan , C. Zhou , Y. Wang , Z. Ma , S. Yao , Int. J. Nanomed. 2022, 17, 713. 10.2147/IJN.S338955 PMC8860399 35210771

[429] N. Liu , G. Li , Y. Guan , R. Wang , Z. Ma , L. Zhao , S. Yao , Ecotoxicol. Environ. Saf. 2022, 241, 113812. 36068741 10.1016/j.ecoenv.2022.113812

[430] F. Zhu , W. Chen , Y. Xu , C. Gao , D. Guan , C. Liu , D. Qian , S.‐C. Zhang , J. Jia , Nature Mater 2015, 14, 1020. 26237127 10.1038/nmat4384

[431] W. Chen , C. Liu , X. Ji , J. Joseph , Z. Tang , J. Ouyang , Y. Xiao , N. Kong , N. Joshi , O. C. Farokhzad , W. Tao , T. Xie , Angew. Chem., Int. Ed. 2021, 60, 7155. 10.1002/anie.202016330 33434327

[432] N. Tao , Z. Zeng , Y. Deng , L. Chen , J. Li , L. Deng , Y.‐N. Liu , Chem. Eng. J. 2023, 456, 141109.

[433] J. Ouyang , L. Zhang , L. Li , W. Chen , Z. Tang , X. Ji , C. Feng , N. Tao , N. Kong , T. Chen , Y.‐N. Liu , W. Tao , Nano‐Micro Lett. 2021, 13, 90. 10.1007/s40820-021-00619-1 PMC8006518 34138343

[434] W. Chen , Y. Li , C. Liu , Y. Kang , D. Qin , S. Chen , J. Zhou , H.‐J. Liu , B. E. Ferdows , D. N. Patel , X. Huang , S. Koo , N. Kong , X. Ji , Y. Cao , W. Tao , T. Xie , Angew. Chem., Int. Ed. 2023, 62, e202308413. 10.1002/anie.202308413 37380606

[435] Z. Tang , N. Kong , J. Ouyang , C. Feng , N. Y. Kim , X. Ji , C. Wang , O. C. Farokhzad , H. Zhang , W. Tao , Matter 2020, 2, 297.

[436] C. Martin , R. Schulz , J. Rose , G. Heusch , Cardiovasc. Res. 1998, 39, 318. 9798517 10.1016/s0008-6363(98)00086-8

[437] G. S. Di Marco , M. Hausberg , U. Hillebrand , P. Rustemeyer , W. Wittkowski , D. Lang , H. Pavenstädt , Am. J. Physiol. Renal. Physiol. 2008, 294, F1381. 18385273 10.1152/ajprenal.00003.2008

[438] W. Chen , J. Ouyang , X. Yi , Y. Xu , C. Niu , W. Zhang , L. Wang , J. Sheng , L. Deng , Y.‐N. Liu , S. Guo , Adv. Mater. 2018, 30, 1703458. 10.1002/adma.201703458 29194780

[439] C. Xing , S. Chen , M. Qiu , X. Liang , Q. Liu , Q. Zou , Z. Li , Z. Xie , D. Wang , B. Dong , L. Liu , D. Fan , H. Zhang , Adv. Healthcare Mater. 2018, 7, 1701510. 10.1002/adhm.201701510 29508554

[440] Z. Chen , Z. Yue , R. Wang , K. Yang , S. Li , Front. Immunol. 2022, 13. 10.3389/fimmu.2022.979469 PMC9441741 36072591

[441] D. Xu , J. Liu , Y. Wang , Y. Jian , W. Wu , R. Lv , ACS Biomater. Sci. Eng. 2020, 6, 4940. 33455288 10.1021/acsbiomaterials.0c00984

[442] B. Geng , W. Shen , P. Li , F. Fang , H. Qin , X. K. Li , D. Pan , L. Shen , ACS Appl. Mater. Interfaces 2019, 11, 44949. 31714729 10.1021/acsami.9b15569

[443] A. Carvalho , M. Wang , X. Zhu , A. S. Rodin , H. Su , A. H. Castro Neto , Nat. Rev. Mater. 2016, 1, 16061.

[444] H. Hashemzadeh , H. Raissi , J. Mol. Liq. 2019, 291, 111346.

[445] M. Qiu , W. Xiu Ren , T. Jeong , M. Won , G. Young Park , D. Kipkemoi Sang , L.‐P. Liu , H. Zhang , J. Seung Kim , Chem. Soc. Rev. 2018, 47, 5588. 29882569 10.1039/c8cs00342d

[446] X. Jing , Z. Zhi , L. Jin , F. Wang , Y. Wu , D. Wang , K. Yan , Y. Shao , L. Meng , Nanoscale 2019, 11, 9457. 31042245 10.1039/c9nr01194c

[447] M. Hayder , M. Poupot , M. Baron , D. Nigon , C.‐O. Turrin , A.‐M. Caminade , J.‐P. Majoral , R. A. Eisenberg , J.‐J. Fournié , A. Cantagrel , R. Poupot , J.‐L. Davignon , Sci. Transl. Med. 2011, 3, 81ra35. 10.1126/scitranslmed.3002212 21543721

[448] F. Zhang , S. Zhang , S. F. Pollack , R. Li , A. M. Gonzalez , J. Fan , J. Zou , S. E. Leininger , A. Pavía‐Sanders , R. Johnson , L. D. Nelson , J. E. Raymond , M. Elsabahy , D. M. P. Hughes , M. W. Lenox , T. P. Gustafson , K. L. Wooley , J. Am. Chem. Soc. 2015, 137, 2056. 25629952 10.1021/ja512616s

[449] S. Hou , S. Chen , Y. Dong , S. Gao , B. Zhu , Q. Lu , ACS Appl. Mater. Interfaces 2018, 10, 25983. 30014692 10.1021/acsami.8b06114

[450] T. Jensen , J. Baas , A. Dolathshahi‐Pirouz , T. Jacobsen , G. Singh , J. V. Nygaard , M. Foss , J. Bechtold , C. Bünger , F. Besenbacher , K. Søballe , J. Biomed. Mater. Res., Part A 2011, 99A, 94. 10.1002/jbm.a.33166 PMC4495906 21800419

[451] M. Zhang , K. Kataoka , Nano Today 2009, 4, 508.

[452] J. Yu , R. Hao , F. Sheng , L. Xu , G. Li , Y. Hou , Nano Res. 2012, 5, 679.

[453] Z. Li , Y. Xin , W. Wu , B. Fu , Z. Zhang , Anal. Chem. 2016, 88, 7724. 27377605 10.1021/acs.analchem.6b01637

[454] N. Bellamri , C. Morzadec , O. Fardel , L. Vernhet , Curr. Opin. Toxicol. 2018, 10, 60.

[455] M.‐M. Wu , H.‐Y. Chiou , I.‐C. Ho , C.‐J. Chen , T.‐C. Lee , Environ. Health Perspect. 2003, 111, 1429. 12928151 10.1289/ehp.6396 PMC1241636

[456] P. Bobé , D. Bonardelle , K. Benihoud , P. Opolon , M. K. Chelbi‐Alix , Blood 2006, 108, 3967. 16926289 10.1182/blood-2006-04-020610

[457] N. Kavian , W. Marut , A. Servettaz , H. Laude , C. Nicco , C. Chéreau , B. Weill , F. Batteux , J. Immunol. 2012, 188, 5142. 22491256 10.4049/jimmunol.1103538

[458] C. Li , T. Guan , C. Gao , Y. Lin , G. Yan , M. Zhu , C. Lv , J. Xia , Z. Qi , Transplant Immunology 2015, 33, 30. 26044521 10.1016/j.trim.2015.05.004

[459] G. Yan , Y. Xi , S. Xu , Y. Lin , J. Chen , H. Dai , J. Xia , C. Li , Q. Li , Z. Li , Z. Qi , Immunol. Invest. 2013, 42, 438. 23802174 10.3109/08820139.2013.801986

[460] E. Bourdonnay , C. Morzadec , O. Fardel , L. Vernhet , J. Cell. Biochem. 2009, 107, 537. 19350554 10.1002/jcb.22155

[461] M. Macoch , C. Morzadec , R. Génard , M. Pallardy , S. Kerdine‐Römer , O. Fardel , L. Vernhet , Free Radic. Biol. Med. 2015, 88, 381. 25680285 10.1016/j.freeradbiomed.2015.02.003

[462] N. K. Maier , D. Crown , J. Liu , S. H. Leppla , M. Moayeri , J. Immunol. 2014, 192, 763. 24337744 10.4049/jimmunol.1301434 PMC3884817

[463] Y. Hu , J. Liang , Y. Xia , C. Zhao , M. Jiang , J. Ma , Z. Tie , Z. Jin , Small 2022, 18, 2104556. 10.1002/smll.202104556 34846791

[464] X. Wang , Y. Hu , J. Mo , J. Zhang , Z. Wang , W. Wei , H. Li , Y. Xu , J. Ma , J. Zhao , Z. Jin , Z. Guo , Angew. Chem. 2020, 132, 5189.

[465] C. Liu , S. Sun , Q. Feng , G. Wu , Y. Wu , N. Kong , Z. Yu , J. Yao , X. Zhang , W. Chen , Z. Tang , Y. Xiao , X. Huang , A. Lv , C. Yao , H. Cheng , A. Wu , T. Xie , W. Tao , Adv. Mater. 2021, 33, 2102054. 10.1002/adma.202102054 34309925

[466] N. Kong , H. Zhang , C. Feng , C. Liu , Y. Xiao , X. Zhang , L. Mei , J. S. Kim , W. Tao , X. Ji , Nat. Commun. 2021, 12, 4777. 34362904 10.1038/s41467-021-24961-5 PMC8346549

[467] N. F. Rosli , C. C. Mayorga‐Martinez , A. C. Fisher , O. Alduhaish , R. D. Webster , M. Pumera , Appl. Mater. Today 2020, 21, 100819.

[468] C. Liu , J. Shin , S. Son , Y. Choe , N. Farokhzad , Z. Tang , Y. Xiao , N. Kong , T. Xie , J. Seung Kim , W. Tao , Chem. Soc. Rev. 2021, 50, 2260. 33367452 10.1039/d0cs01175d

[469] F. Gendrisch , B. Haarhaus , C. M. Schempp , U. Wölfle , Molecules 2021, 26, 5814. 34641358 10.3390/molecules26195814 PMC8510055

[470] L. Bregoli , F. Chiarini , A. Gambarelli , G. Sighinolfi , A. M. Gatti , P. Santi , A. M. Martelli , L. Cocco , Toxicology 2009, 262, 121. 19482055 10.1016/j.tox.2009.05.017

[471] J. Zia , P. M. Rashad , U. Riaz , J. Mater. Res. Technol. 2019, 8, 4079.

[472] Z. Miao , L. Fan , X. Xie , Y. Ma , J. Xue , T. He , Z. Zha , ACS Appl. Mater. Interfaces 2019, 11, 26664. 31287947 10.1021/acsami.9b08320

[473] Y. Duo , Y. Huang , W. Liang , R. Yuan , Y. Li , T. Chen , H. Zhang , Adv. Funct. Mater. 2020, 30, 1906010.

[474] W. Tao , X. Ji , X. Zhu , L. Li , J. Wang , Y. Zhang , P. E. Saw , W. Li , N. Kong , M. A. Islam , T. Gan , X. Zeng , H. Zhang , M. Mahmoudi , G. J. Tearney , O. C. Farokhzad , Adv. Mater. 2018, 30, 1802061. 10.1002/adma.201802061 PMC7028391 30043416

[475] Y. Kang , Z. Li , Y. Yang , Z. Su , X. Ji , S. Zhang , Adv. Healthcare Mater. 2021, 10, 2001835. 10.1002/adhm.202001835 33200585

[476] Y. Yang , R. Ouyang , L. Xu , N. Guo , W. Li , K. Feng , L. Ouyang , Z. Yang , S. Zhou , Y. Miao , J. Coord. Chem. 2015, 68, 379.

[477] R. Ge , H. Sun , Acc. Chem. Res. 2007, 40, 267. 17330963 10.1021/ar600001b

[478] D. M. Keogan , D. M. Griffith , Molecules 2014, 19, 15258. 25251194 10.3390/molecules190915258 PMC6271281

[479] R. Zhou , Q. Zhou , G. Ling , P. Zhang , Colloids Surf. A 2023, 660, 130832.

[480] R. Huang , Z. Zhou , X. Lan , F. K. Tang , T. Cheng , H. Sun , K. Cham‐Fai Leung , X. Li , L. Jin , Materials Today Bio. 2023, 18, 100507. 10.1016/j.mtbio.2022.100507 PMC9730226 36504541

[481] I. E. Mba , E. I. Nweze , World J. Microbiol. Biotechnol. 2021, 37, 108. 34046779 10.1007/s11274-021-03070-x PMC8159659

[482] K. Szostak , P. Ostaszewski , J. Pulit‐Prociak , M. Banach , Pharm. Chem. J. 2019, 53, 48.

[483] R. Chai , L. Yu , C. Dong , Y. Yin , S. Wang , Y. Chen , Q. Zhang , Bioact. Mater. 2022, 17, 276. 35386463 10.1016/j.bioactmat.2022.01.014 PMC8965086

[484] H. Song , J. Wang , B. Xiong , J. Hu , P. Zeng , X. Liu , H. Liang , Angew. Chem. 2022, 134, e202117679. 10.1002/anie.202117679 35257450

[485] M. Guo , X. Zhang , J. Liu , F. Gao , X. Zhang , X. Hu , B. Li , X. Zhang , H. Zhou , R. Bai , Y. Wang , J. Li , Y. Liu , Z. Gu , C. Chen , ACS Nano 2020, 14, 15700. 33155807 10.1021/acsnano.0c06656

[486] Y. Zhu , Y. Wu , S. Li , X. Yuan , J. Shen , S. Luo , Z. Wang , R. Gao , J. Wu , L. Ge , Chem. Eng. J. 2022, 446, 137321.

[487] C. Zhao , X. Cai , X. Liu , J. Wang , W. Chen , L. Zhang , Y. Zhang , Z. Zhu , C. Liu , C. Niu , Y. Jia , Phys. Chem. Chem. Phys. 2022, 24, 7512. 35289820 10.1039/d2cp00070a

[488] I. Muneta , T. Shirokura , P. N. Hai , K. Kakushima , K. Tsutsui , H. Wakabayashi , Sci. Rep. 2022, 12, 17199. 36229486 10.1038/s41598-022-22113-3 PMC9562137

[489] N. Sp , D. Y. Kang , H. D. Kim , A. Rugamba , E. S. Jo , J.‐C. Park , S. W. Bae , J.‐M. Lee , K.‐J. Jang , Life 2021, 11, 427. 34068523 10.3390/life11050427 PMC8151259

[490] N. M. Hashem , A. E.‐D. M. S. Hosny , A. A. Abdelrahman , S. Zakeer , N. M. Hashem , A. E.‐D. M. S. Hosny , A. A. Abdelrahman , S. Zakeer , AIMSMICRO 2021, 7, 481.

[491] H. Baloch , A. Siddiqua , A. Nawaz , M. S. Latif , S. Q. Zahra , S. Y. Alomar , N. Ahmad , T. M. Elsayed , Gels 2023, 9, 284. 37102896 10.3390/gels9040284 PMC10137662

[492] J. Cao , Y. Zhang , Y. Yang , J. Xie , Z. Su , F. Li , J. Li , B. Zhang , Z. Wang , P. Zhang , Z. Li , L. He , H. Liu , W. Zheng , S. Zhang , A. Hong , X. Chen , J. Nanobiotechnol. 2023, 21, 57. 10.1186/s12951-023-01796-4 PMC9942369 36803772

[493] K. Samrat , M. N. Chandraprabha , R. Hari Krishna , R. Sharath , B. Vadappi , Mater. Technol. 2022, 37, 3025.

[494] X. Li , S. Zhao , K. Yang , B. Li , B. Wang , J. Yi , X. Song , M. Lan , Chem. Commun. 2022, 58, 6251. 10.1039/d2cc01165d 35510707

[495] Z. Bai , L. Shen , J. Wei , Y. Li , A. Abbas , Y. Li , M. Qu , D. Zhang , C. Zhang , ACS Appl. Nano Mater. 2020, 3, 10749.

[496] “Selenium as a ‘nutraceutical’: how to conciliate physiological and supra‐nutritional effects for an essential trace element,” can be found under https://oce‐ovid‐com.uml.idm.oclc.org/article/00075197‐200211000‐00008/HTML, n.d. 10.1097/00075197-200211000-00008 12394641

[497] M. P. Rayman , Lancet 2000, 356, 233. 10963212 10.1016/S0140-6736(00)02490-9

[498] Institute of Medicine, Food and Nutrition Board , Dietary Reference Intakes : Applications in Dietary Assessment, National Academies Press, Washington, D.C., 2000.

[499] P. Sonkusre , S. S. Cameotra , J. Nanobiotechnol. 2017, 15, 10.1186/s12951-017-0276-3. PMC5463494 28592284

[500] X. Wang , K. Sun , Y. Tan , S. Wu , J. Zhang , Free Radic. Biol. Med. 2014, 72, 1. 24727439 10.1016/j.freeradbiomed.2014.04.003

[501] Y. Feng , J. Su , Z. Zhao , W. Zheng , H. Wu , Y. Zhang , T. Chen , Dalton Trans. 2014, 43, 1854. 24257441 10.1039/c3dt52468j

[502] S. Zheng , X. Li , Y. Zhang , Q. Xie , Y.‐S. Wong , W. Zheng , T. Chen , IJN 2012, 7, 3939. 22915845 10.2147/IJN.S30940 PMC3418171

[503] Y. Ren , T. Zhao , G. Mao , M. Zhang , F. Li , Y. Zou , L. Yang , X. Wu , Int. J. Biol. Macromol. 2013, 57, 57. 23500433 10.1016/j.ijbiomac.2013.03.014

[504] J. C. Avery , P. R. Hoffmann , Nutrients 2018, 10, 1203.30200430

[505] R. Brigelius‐Flohé , M. Maiorino , Biochim. Biophys. Acta, Gen. Subj. 2013, 1830, 3289. 10.1016/j.bbagen.2012.11.020 23201771

[506] J. P. Thomas , M. Maiorino , F. Ursini , A. W. Girotti , J. Biol. Chem. 1990, 265, 454. 2294113

[507] D. Daily , A. Vlamis‐Gardikas , D. Offen , L. Mittelman , E. Melamed , A. Holmgren , A. Barzilai , J. Biol. Chem. 2001, 276, 1335. 11035035 10.1074/jbc.M008121200

[508] E.‐M. Hanschmann , J. R. Godoy , C. Berndt , C. Hudemann , C. H. Lillig , Antioxid. Redox Signaling 2013, 19, 1539. 10.1089/ars.2012.4599 PMC3797455 23397885

[509] I. Ahrens , C. Ellwanger , B. K. Smith , N. Bassler , Y. C. Chen , I. Neudorfer , A. Ludwig , C. Bode , K. Peter , J. Leukocyte Biol. 2008, 83, 1388. 18305178 10.1189/jlb.0707497

[510] M. H. Yazdi , M. Mahdavi , N. Setayesh , M. Esfandyar , A. R. Shahverdi , DARU J. Pharm. Sci. 2013, 21, 10.1186/2008-2231-21-33. PMC3658950 23631392

[511] K. Spyridopoulou , G. Aindelis , A. Pappa , K. Chlichlia , Cancers 2021, 13, 5335. 34771499 10.3390/cancers13215335 PMC8582357

[512] K. Spyridopoulou , E. Tryfonopoulou , G. Aindelis , P. Ypsilantis , C. Sarafidis , O. Kalogirou , K. Chlichlia , Nanoscale Adv 2021, 3, 2516. 36134160 10.1039/d0na00984a PMC9417964

[513] G. Liao , J. Tang , D. Wang , H. Zuo , Q. Zhang , Y. Liu , H. Xiong , World J. Surg. Oncol. 2020, 18, 10.1186/s12957-020-01850-7. PMC7195723 32357938

[514] E. M. M. Ebrahem , G. H. Sayed , G. N. A. Gad , K. E. Anwer , A. A. Selim , Cancer Nanotechnol. 2022, 13, 14.

[515] A. A. Mohamed , R. A. Zaghloul , A. M. Abdelghany , A. M. El Gayar , J. Biochem. Mol. Toxicol. 2022, 36, e22989. 35179263 10.1002/jbt.22989

[516] M. A. El‐Ghazaly , N. Fadel , E. Rashed , A. El‐Batal , S. A. Kenawy , Can. J. Physiol. Pharmacol. 2017, 95, 101. 27936913 10.1139/cjpp-2016-0183

[517] S. Malhotra , M. N. Welling , S. B. Mantri , K. Desai , J. Biomed. Mater. Res., Part B 2016, 104, 993. 10.1002/jbm.b.33448 25994972

[518] X. Zhang , H. Yan , L. Ma , H. Zhang , D.‐F. Ren , J. Food Biochem. 2020, 44, e13363. 32648615 10.1111/jfbc.13363

[519] G. S. Kumar , A. Kulkarni , A. Khurana , J. Kaur , K. Tikoo , Chem.‐Biol. Interact. 2014, 223, 125. 25301743 10.1016/j.cbi.2014.09.017

[520] L. Guo , J. Xiao , H. Liu , H. Liu , Metallomics 2020, 12, 204. 31793592 10.1039/c9mt00215d

[521] X. Liu , Y. Mao , S. Huang , W. Li , W. Zhang , J. An , Y. Jin , J. Guan , L. Wu , P. Zhou , Regen. Biomater. 2022, 9, rbac042. 35855111 10.1093/rb/rbac042 PMC9290869

[522] X. Yuan , Z. Fu , P. Ji , L. Guo , A. O. Al‐Ghamdy , A. Alkandiri , O. A. Habotta , A. E. Abdel Moneim , R. B. Kassab , IJN 2020, 15, 6339. 32922005 10.2147/IJN.S259134 PMC7455605

[523] A. Albrakati , K. F. Alsharif , N. E. A. Omairi , W. F. Alsanie , A. S. A. Almalki , Z. Y. A. Elmageed , G. E. Elshopakey , M. S. Lokman , A. A. Bauomy , A. E. A. Moneim , R. B. Kassab , Int. J. Nanomed. 2021, 15, 8447. 10.2147/IJN.S323436 PMC8722537 35002238

[524] L. Xian , A. P. Paz , E. Bianco , P. M. Ajayan , A. Rubio , 2D Mater. 2017, 4, 041003.

[525] J. Qin , G. Qiu , J. Jian , H. Zhou , L. Yang , A. Charnas , D. Y. Zemlyanov , C.‐Y. Xu , X. Xu , W. Wu , H. Wang , P. D. Ye , ACS Nano 2017, 11, 10222. 28949510 10.1021/acsnano.7b04786

[526] C. Xing , Z. Xie , Z. Liang , W. Liang , T. Fan , J. S. Ponraj , S. C. Dhanabalan , D. Fan , H. Zhang , Adv. Opt. Mater. 2017, 5, 1700884.

[527] Z. Xie , C. Xing , W. Huang , T. Fan , Z. Li , J. Zhao , Y. Xiang , Z. Guo , J. Li , Z. Yang , B. Dong , J. Qu , D. Fan , H. Zhang , Adv. Funct. Mater. 2018, 28, 1705833.

[528] W. Pan , C. Liu , Y. Li , Y. Yang , W. Li , C. Feng , L. Li , Bioact. Mater. 2022, 13, 96. 35224294 10.1016/j.bioactmat.2021.11.010 PMC8843971

[529] Y. Lin , Y. Wu , R. Wang , G. Tao , P.‐F. Luo , X. Lin , G. Huang , J. Li , H.‐H. Yang , Chem. Commun. 2018, 54, 8579. 10.1039/c8cc04653k 30019046

[530] P. Pishva , M. Yüce , Emergent Mater. 2021, 4, 211. 33615139 10.1007/s42247-021-00184-8 PMC7880038

[531] X. Peng , Y. Zhou , K. Nie , F. Zhou , Y. Yuan , J. Song , J. Qu , New J. Phys. 2020, 22, 103046.

[532] H. Vahidi , F. Kobarfard , A. Alizadeh , M. Saravanan , H. Barabadi , Inorg. Chem. Commun. 2021, 124, 108385.

[533] E. R. T. Tiekink , Dalton Trans. 2012, 41, 6390. 22252404 10.1039/c2dt12225a

[534] E. Domínguez‐Álvarez , B. Rácz , M. A. Marć , M. J. Nasim , N. Szemerédi , J. Viktorová , C. Jacob , G. Spengler , Drug Resist. Updates 2022, 63, 100844. 10.1016/j.drup.2022.100844 35533630

[535] S. Chen , C. Xing , D. Huang , C. Zhou , B. Ding , Z. Guo , Z. Peng , D. Wang , X. Zhu , S. Liu , Z. Cai , J. Wu , J. Zhao , Z. Wu , Y. Zhang , C. Wei , Q. Yan , H. Wang , D. Fan , L. Liu , H. Zhang , Y. Cao , Sci. Adv. 2020, 6, eaay6825. 32284997 10.1126/sciadv.aay6825 PMC7141822

[536] M. Gross , E. Stanciu , D. Kenigsbuch‐Sredni , B. Sredni , A. Pinhasov , Behav. Pharmacol. 2017, 28, 458. 28590303 10.1097/FBP.0000000000000319

[537] P. Du , N. E. B. Saidu , J. Intemann , C. Jacob , M. Montenarh , Biochim. Biophys. Acta (BBA) – Gen. Subjects 2014, 1840, 1808. 10.1016/j.bbagen.2014.02.003 24530428

[538] M. Doostmohammadi , H. Forootanfar , M. Shakibaie , M. Torkzadeh‐Mahani , H.‐R. Rahimi , E. Jafari , A. Ameri , A. Ameri , IET Nanobiotechnology 2021, 15, 277. 34694673 10.1049/nbt2.12020 PMC8675828

[539] Y. Wu , T. Guo , Y. Qiu , Y. Lin , Y. Yao , W. Lian , L. Lin , J. Song , H. Yang , Chem. Sci. 2019, 10, 7068. 31588274 10.1039/c9sc01070j PMC6676468

[540] J.‐W. Liu , J.‐H. Zhu , C.‐L. Zhang , H.‐W. Liang , S.‐H. Yu , J. Am. Chem. Soc. 2010, 132, 8945. 20545345 10.1021/ja910871s

[541] W. Huang , H. Wu , X. Li , T. Chen , ChemistryAsian J. 2016, 11, 2301. 10.1002/asia.201600757 27325381

